# Multiple Classes of Antigen Contribute to the Antigenic Landscape of Mesothelioma

**DOI:** 10.1016/j.mcpro.2025.100925

**Published:** 2025-02-05

**Authors:** Kirti Pandey, Pouya Faridi, Rochelle Ayala, Y.C. Gary Lee, Ebony Rouse, Sanjay S.G. Krishna, Ian Dick, Alec Redwood, Bruce Robinson, Jenette Creaney, Anthony W. Purcell

**Affiliations:** 1 Department of Biochemistry and Molecular Biology and Infection and Immunity Program, Biomedicine Discovery Institute, Monash University, Melbourne, Victoria, Australia; 2 Department of Medicine, School of Clinical Sciences, Faculty of Medicine, Nursing & Health Sciences, Monash University, Melbourne, Australia; 3 National Centre for Asbestos Related Diseases, University of Western Australia, Perth, Australia; 4 Department of Respiratory Medicine, Sir Charles Gairdner Hospital, Perth, Australia

**Keywords:** mesothelioma, asbestos, cancer, HLA class I, immunopeptidomics, mass spectrometry, ERV, LINE, LTR

## Abstract

Mesothelioma is an incurable, asbestos-exposure-related cancer that typically affects the lining or pleura of the lungs. Symptoms typically develop many decades after initial asbestos exposure, leaving an enduring legacy of disease. The current disease burden is peaking worldwide and thus there is a massive unmet clinical need for curative therapies. Recently, immune checkpoint blockade-based therapy has been adopted as a first-line of treatment for mesothelioma. Vaccine-induced augmentation of immune responses unleashed during checkpoint blockade may provide further clinical benefit in mesothelioma. In this study, we explore the human leukocyte antigen class I landscape (or immunopeptidome) of mesothelioma in patient-derived cell lines and clinical material (pleural effusion samples). We identify a range of peptide antigens derived from targets including cancer testis antigens, endogenous retroviruses as well as novel post-translational modification of peptides. This information will facilitate the characterization of the immune response to these antigens to determine which class of antigen is most immunogenic and has the potential to be tested in future vaccine studies.

## Highlights

• Comprehensive investigation of the mesothelioma HLA class I landscape from both cell lines and pleural effusions.
• Over 2000 peptides of interest identified including those derived from known tumor antigens HERVs, CTAs, and TAAs.
• A unique cysteinylation signature was seen in PTM peptides isolated from mesothelioma samples.

## Graphical Abstract

**Figure:**
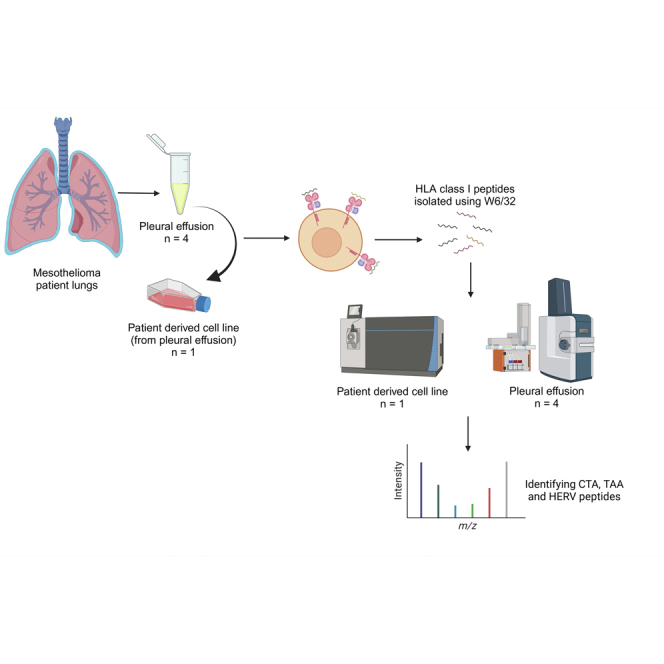

## In Breif

The HLA class I landscape of mesothelioma has largely remained unexplored. Here we apply state-of-the-art immunopeptidomics to explore potential targets of T cell-mediated antitumor immunity using a patient-derived mesothelioma cell line and pleural effusion samples from multiple donors. Using this unbiased approach, we explore several classes of peptide antigens and compare the prevalence of mutational, post-translational and tumor-associated antigen-derived peptide ligands.

Mesothelioma is an incurable, asbestos-exposure-related cancer. In 2020, there were an estimated 30,870 mesothelioma cases reported globally, with the highest age incidence rates found in Northern Europe, Australia, and New Zealand. Moreover, incidences are on the rise across the globe with a prevalence of 0.30 per 100,000 persons (1, 2). Mesothelioma arises in the mesothelial lining of pleural, peritoneal, and pericardial cavities, and pleural mesothelioma accounts for 65 to 80% of all cases (3). Symptoms typically develop many decades after initial asbestos exposure. Australia banned the use of asbestos in 2004, but before this, it was widely used in the construction and transport industries. As such, large amounts of asbestos remain in buildings and other infrastructure, leaving an enduring legacy of disease.

The life expectancy for most patients with malignant mesothelioma (MM) is only 12 months post-diagnosis. Current forms of treatment can include surgery, immunotherapy, chemotherapy or radiation, which may improve this prognosis but does not cure the disease. Current disease burden is peaking in countries like Australia (4) and thus there is a massive unmet clinical need. Results from checkpoint blockade studies suggest that vaccine-induced augmentation of immune responses may provide additional clinical benefit in mesothelioma, however, there are only a few known T cell epitopes and vaccine targets (5). In this study, we explored the antigenic landscape of mesothelioma using a combination of genomics and immunopeptidomics (i.e. the analysis and identification of the peptides presented by HLA molecules for recognition by T cells) to facilitate the characterization of the immune response to these antigens and determine the most appropriate classes of antigen for future vaccine studies.

The tumor mutation burden (TMB) in mesothelioma is very low compared to other cancers such as melanoma and colorectal cancer (6). Therefore, it becomes essential to explore different classes of peptide antigens that may be relevant to mesothelioma. This can include not only mutational neoantigens but also post-translationally modified (PTM) peptide antigens, cancer-testis antigens (CTA), and other tumor-associated antigens (TAA). In addition, we also searched for peptides derived from endogenous retroviruses (ERVs). These are genetic elements derived from ancient retroviral infections that have become integrated into the genome of the host organism [6] and comprise approximately 8% of the human genome (7, 8). Mesothelioma is one of the cancer types with the highest number of expressed cancer-specific long terminal repeats (LTR) retroelements (9). Recent studies in mice (10) and patients (11) have elucidated that exposure to asbestos leads to increased expression of human ERVs in mesothelioma, particularly LTRs and long-interspersed nuclear elements (LINEs).

## Experimental Procedures

### Experimental Design and Statistical Rationale

The mesothelioma cell line MMCL_09567 was derived from a pre-treatment pleural effusion (PE) of a 70-year-old male with epithelioid mesothelioma. As previously reported (12) following whole exome sequencing 41 tumor-specific nonsynonymous mutations were identified for this cell line and its haplotype (Table 1), and 67 neoantigens were predicted to bind the HLA using NetMHCpan (v 2.8) with a half-maximal inhibitory concentration (IC50) value of <500 nM.

**Table 1:** HLA type of cell line and PE samples included in the study

Patient ID	Sample type	MPM subtype	Number of missense mutations identified in sample	HLA a	HLA B	HLA C
BR_P6215	PE	Epithelioid	31	01:01, 32:01	08:01, 40:01	03:04, 07:01
RK_P4724	PE	Epithelioid	35	25:01, 68:01	39:01, 44:03	07:01, 12:03
GC_7089	PE	Epithelioid	37	01:01, 24:01	08:01, 55:01	03:01, 07:01
BM_P7462	PE	Epithelioid	28	23:01	07:02, 44:03	04:01, 07:02
MMCL_09567	Cell line	Epithelioid	41	02:05, 11:01	44:03, 49:01	07:01, 16:01

PE samples were collected from patients (n = 4) as previously described (13) as part of the NCARD biobanking program. Briefly, pleural effusions were drained as clinically indicated, centrifuged and the total cell fraction resuspended in RPMI-1640 (Sigma-Aldrich) supplemented with 30% fetal calf serum (Invitrogen) and 10% dimethylsulfoxide (Sigma-Aldrich) then transferred to −80 °C for storage until later analysis. All four patients were diagnosed with epithelioid MM and carried missense (ranging from 28-37 in number) mutations (Table 1). The HLA typing of the PE samples is mentioned in Table 1. The study was approved by Sir Charles Gairdner and Osborne Park Hospitals Human Research Ethics Committee, number 2005-038 (RGS0000001516) and all participants provided written informed consent and conformed with the Code of Ethics of the World Medical Association (Declaration of Helsinki).

#### Immunoprecipitation of Peptide-HLA Complexes for Cell Line

An immunoaffinity matrix was generated wherein 10 mg/ml of W6/32 pan-HLA antibody was crosslinked to 1 ml of Protein A Sepharose (PAS, CaptivA) resin as previously described (14) and used for the immunoaffinity capture of solubilized native peptide-HLA (pHLA) complexes. Briefly, frozen cell pellets for patient-derived mesothelioma cell line MMCL_9567 (1 × 10^9^) were pulverized by cryogenic milling (Retsch Mixer Mill MM 400), reconstituted in Lysis Buffer [0.5% IGEPAL (Sigma-Aldrich), 50 mM Tris pH 8, 150 mM NaCl and protease inhibitors (Complete Protease Inhibitor Cocktail Tablet, one tablet per 50 ml solution; Roche Molecular Biochemicals, Switzerland)] and proteins solubilized by incubation for 1 h at 4 °C with rotation. The supernatant was passed through a PAS pre-column (500 μl) to remove non-specific binding material, followed by affinity capture of class I peptides (W6/32 column) for the cell line. Bound peptide-HLA complexes were eluted from the column in 10% acetic acid. The eluate was fractionated by RP-HPLC into 48 fractions which were concatenated, vacuum concentrated, into 10 fractions, and reconstituted in 0.1% formic acid (FA).

#### Small-Scale Immunopeptidomics Analysis of PE Samples

Small scale immunoaffinity purifications were performed on PE samples (2 × 10^6^) as previously described (15). Briefly, cell pellets were lysed with 300 μl of lysis buffer (0.5% IGEPAL, 50 mM Tris [pH 8.0], 150 mM NaCl and 1X protease inhibitor tablet [cOmpleteTM Protease Inhibitor Cocktail Tablet; Roche Molecular Biochemicals]), mixed gently and incubated on a roller at 4 °C for 1 h. PE samples were lysed at 4 °C for 45 min and samples were centrifuged at 3724*g*, 10 min. HLA class I peptides were pulled down using W6/32 (pan HLA class I) antibody bound to PAS resin. To remove contaminants such as detergent, salt, and nonspecific binders, all columns were washed with five column volumes of 1X PBS. Bound peptide-HLA (pHLA) complexes were eluted using 300 μl of 10% acetic acid. To separate the proteinaceous material from peptides, the eluate was passed through a 5 kDa molecular weight cut off filter (Amicon, Sigma-Aldrich) and centrifuged at 16,060*g* for 30 min at room temperature (RT).

#### Analysis of HLA Class I Bound Peptides Derived from a Mesothelioma Cell Line Using an Orbitrap Fusion LC-MS System

All 10 pooled peptide-containing fractions were analyzed by Orbitrap Fusion Tribrid (Thermo Scientific) using liquid chromatography-tandem mass spectrometry (LC-MS/MS) as described previously (16). Peptides were loaded onto a PepMap Acclaim 100 C18 trap column (5 μm particle size, 100 μm × 2 cm and 100 Å (Thermo Scientific)) at 15 μl/min using an Ultimate 3000 RSLC nano-HPLC (Thermo Scientific). The column was equilibrated with 2% acetonitrile (ACN) and 0.1% FA. Peptides were eluted and separated at a flow rate of 250 nl/min on an in-line analytical column (PepMap RSLC C18, 2 μm particle size, 75 μm × 50 cm and 100 Å, Thermo Scientific) using a 125 min linear gradient of 2.5 to 99% of buffer B (80% ACN, 0.1%FA). The gradient started from 2.5% buffer B and increased to 7.5% buffer B in a minute followed by a linear gradient to 37.5% buffer B for 90 min followed by an increase to 99% buffer B in 10 min. Peptides were introduced using nano-electrospray ionization (nano-ESI) method at a source temperature of 275 °C.

All MS spectra (MS1) profiles were recorded from full ion scan mode 375 to 1800 m/z, in the Orbitrap at 120,000 resolution with automatic gain control (AGC) target of 400,000 and dynamic exclusion of 15 s. The top 12 precursor ions were selected for MS/MS using top speed mode at a cycle time of 2 s. For MS/MS a decision tree was made to aid in selecting peptides of charge state one and 2 to 6 separately. For singly charged analytes only ions falling within the range of m/z 800 to 1800 were selected, whereas for +2 to +6 m/z no such parameter was set. The C-trap was loaded with a target of 200,000 ions with an accumulation time of 120 ms and an isolation width of 1.2 amu. The normalized collision energy was set to 32 (high energy collisional dissociation [HCD]) and MS/MS fragment ions were analyzed in the Orbitrap at 30,000 resolution.

#### Analysis of HLA Class I Bound Peptides in PE Samples Using an Evosep One - Bruker Tims TOF Pro 2 LC-MS System

A mixture of 11 indexed retention time (iRT) peptides were spiked into the 4 PE samples and 10 peptide-containing fractions derived from the eluted peptides from the cell line to aid retention time alignment (17). The PE samples were loaded onto Puretips (Evosep Biosystems) as per the manufacturer’s instructions. Evosep one liquid chromatography (LC) system was used to acquire the samples on the Bruker Tims TOF Pro2 instrument. Briefly, samples were eluted and separated on an Aurora Elite column (IonOpticks, 15 cm × 75um × 1.7 um, 120 A pore size) using Whisper 20 SPD (flow rate of 100 nl/min and an hour-long gradient) at 50 °C. Mobile phases A and B consisted of 0.1% FA in 2% ACN and 0.1% FA in ACN, respectively. The peptides eluted from the column were analyzed using a hybrid trapped ion mobility-quadrupole time of flight mass spectrometer (Bruker timsTOF Pro 2, Bruker Daltonics). The data-dependent acquisition was performed with the following settings: m/z range: 100 to 1700mz, capillary voltage:1600V, Target intensity of 30,000, TIMS ramp of 0.60 to 1.60 Vs/cm two for 166 ms.

### MS Data Search Parameters

The raw data files obtained from the mass spectrometer were analyzed using Peaks Online software (ver 11, Bioinformatics Solutions Inc) and searched against the human proteome (Uniprot 25/06/2022; 20,483 entries). The mutations identified in the cell line and PE samples using genomics along with iRT peptide sequences were appended to the Uniprot database to allow for the identification of any neoantigen-derived peptides. The HERV database was added as a contamination database.

The following search parameters were used for the cell line (data acquired on Thermo Tribrid Fusion): error tolerance of 10 ppm using monoisotopic mass for precursor ions and 0.02 Da tolerance for fragment ions; enzyme used was set to none with the following variable modifications: oxidation at Met (M), deamidation at Asp (D) and Gln (Q). To identify other prevalent PTM peptides in the cell line a Peaks PTM search (which includes 312 in-built PTMs) was performed. These settings were also used for researching the healthy lung data obtained from the HLA ligand atlas (18).

The PE samples were acquired on Bruker TimsTOF Pro2. The following search parameters were used for the PE samples: error tolerance of 15 ppm using monoisotopic mass for precursor ions and 0.05 Da tolerance for fragment ions. Enzyme used was set to none with following variable modifications: oxidation at Met (M), deamidation at Asp (D) and Gln (Q), Phosphorylation at Ser (S), Thr (T) and Tyr (Y) and cysteinylation of Cys (C). The false discovery rate (FDR) was estimated using a decoy fusion method (19) for both cell line and PE samples and all datasets were analyzed using a 5% FDR cut off.

For data filtering first, iRT peptides were removed. Second, only peptides 8 to 12 amino acid in length (with and without PTMs) were retained. To examine the presentation of HERV peptides by the cell line and pleural effusion samples we used the Genome-based Endogenous Viral Element Database (gEVE database (20), http://geve.med.u-tokai.ac.jp/). For HERV peptides a few more steps were followed. First, peptides with duplicate scan numbers (due to I/L ambiguity) were counted as one. Second, any HERV peptide that had a scan number (alternative explanation) in the main human peptide CSV was also removed.

### HERV Peptide Validation

HERV peptides identified in the PE samples were validated using synthetic peptides (SP) that were synthesized using standard Fmoc chemistry (Mimotopes). Peptides were reconstituted at 10uM stock concentration using DMSO. Peptides were pooled and MS/MS spectra were acquired using the original data acquisition method. MS fragmentation information was obtained from mgf files based on scan number and mirror plots were generated for experimental vs synthetic peptides using Universal Spectrum Explorer (USE) (21). Pearson’s Correlation Coefficient (PCC) and Spectral Angle (SA) scores which show the extent of spectral matching were calculated (21). For HERV peptides identified in cell line spectra MS/MS prediction by PROSIT (HCD 2020 model) was used to validate the peptides.

Retention time (RT) prediction was performed using the DeepLC prediction algorithm (22). Eighty percent of canonical peptides were used for training the model which was in turn used for predicting the RT of the HERV peptides. The models for PE and cell line samples were trained separately. Prediction performance was measured using three commonly used metrics: mean absolute error (MAE), PCC, and R^2^.

### MS Data Analysis

The data was plotted in GraphPad Prism (Version 8.4). The binding affinity of the peptides was predicted using NetMHC 4.1 (23) and MHCflurry 2.0 (24). For NetMHC, peptides with a rank threshold of less than 0.5 were considered as strong binders (SB), whilst those between 0.5 to 2.0 were considered as weak binders (WB). For MHCflurry, a commonly used threshold of 500 nM in the MHCflurry affinity column was considered to identify binders. Consensus binding motifs were plotted using Icelogo (25). To identify the cellular compartments from which source proteins were originating, gene ontology (GO) term mapper (https://go.princeton.edu/cgi-bin/GOTermMapper) was used. To identify CTA and TAA-derived peptides we used CTdatabase (http://www.cta.lncc.br/) and Tumor T-cell antigen database (TANTIGEN, http://cvc.dfci.harvard.edu/tantigen), respectively.

### Conflicts of Interest

AWP is a scientific advisor for Bioinformatics Solutions Inc and Grey Wolf Therapeutics, a shareholder and scientific advisor for Evaxion Biotech (Denmark), and a co-founder of Resseptor Therapeutics. The funders had no role in the design of the study; in the collection, analyses, or interpretation of data; in the writing of the manuscript; or in the decision to publish the results.

## Results

### Mapping Endogenously Presented HLA Class I Peptide Repertoire in Human Mesothelioma Samples

**Fig. 1:**
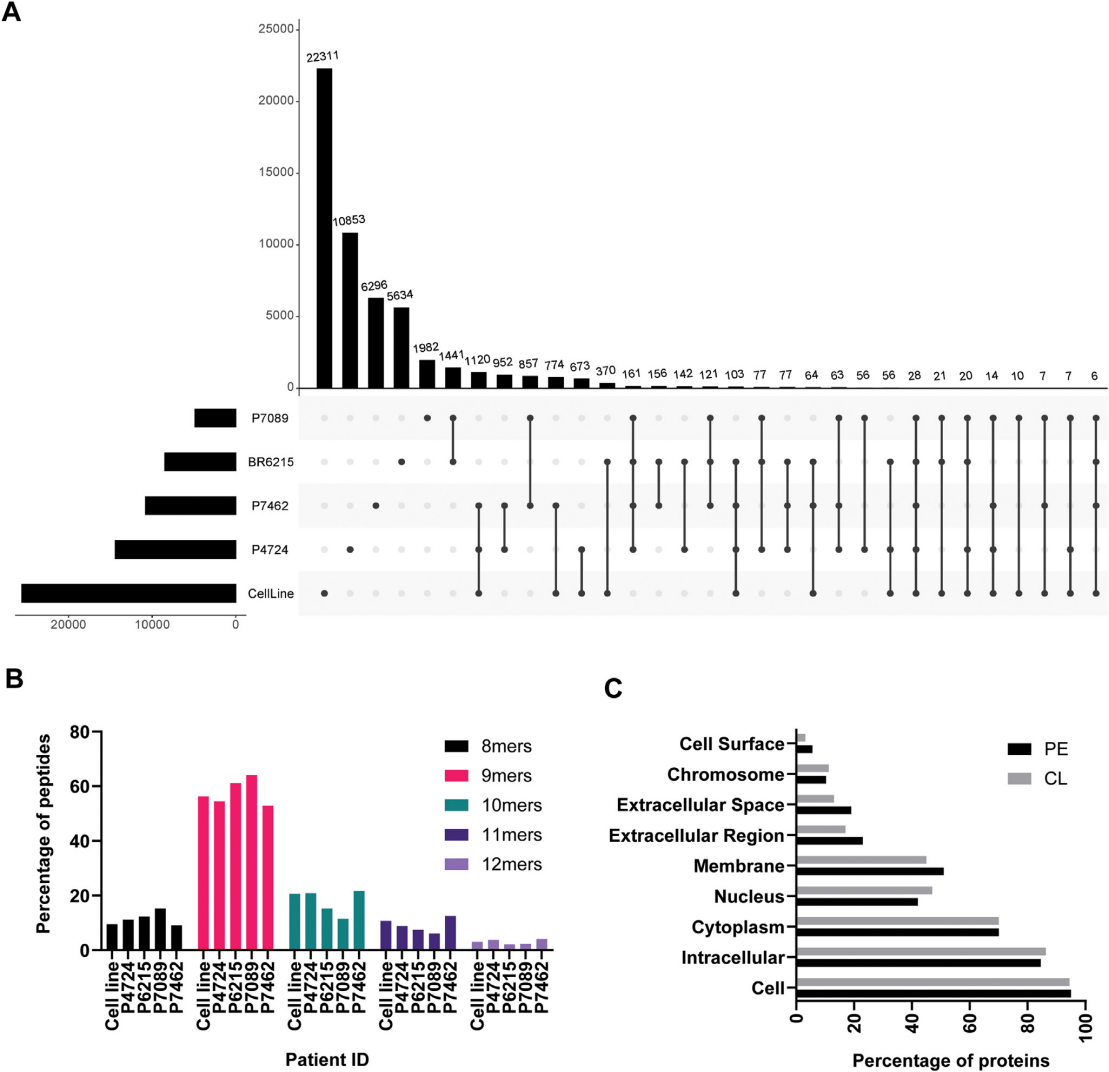
**The immunopeptidomic landscape of mesothelioma.***A*, upset plot depicting the overlap between HLA class I bound peptides (8mers – 12mers only) identified in patient-derived mesothelioma cell line (n = 1) and PE samples (n = 4). *B*, peptide length distribution of peptides identified across the sample subtypes following purification of pHLA using pan class I monoclonal antibody W6/32. *C*, gene ontology analysis of the source proteins identified in cell line and PE samples.

A total of 54,279 non-redundant HLA class I bound peptides (8–12 amino acids long) were identified from mesothelioma cell line (n = 1) and pleural effusion samples (n = 4) using a pan class I immunoprecipitation and LC-MS/MS analysis (Supplemental data file S1). The peptides identified in the study were restricted to 29 different HLA class I alleles across eight HLA-A, seven HLA-B and seven HLA-C alleles with HLA-B∗44:03 (n = 3), -C∗07:01 (n = 4) and -A∗01:01 (n = 2) being the most commonly represented alleles (Table 1). Despite the number of shared alleles there was an overlap of only 6% (3275) between the peptides identified in the cell line and across the four PE samples (Fig. 1*A*). As evident from the 9mer motif of the shared peptides, they belonged to the two HLA allotypes (HLA - B∗44:03 and -C∗07:01) shared between the cell line and four PE samples (Supplemental Fig. S1*A*). The peptides identified in the study followed a canonical class I length distribution with the majority of the peptides (52% to 64%) being nonamers (Fig. 1*B*). Based on the NetMHC binding prediction for the nonamer peptides identified from the PE samples (except P4724), we observed that there was a high percentage of peptides (31% to 39%) predicted to be restricted to HLA C allotypes (Supplemental Fig. S1*B*). This was confirmed using another prediction algorithm MHCflurry 2.0 (also ∼35%) (Supplemental Fig. S1*B*). In contrast, for the cell line there was a predominance of peptides restricted to HLA-A and -B allotypes (Supplemental Fig. S1*B*). The binding predictions for the PE and cell line samples are part of Supplemental data file S2.

The peptides identified in the study originated from 10,023 non-redundant source proteins. At the source protein level, there was a much higher overlap of 52% between all samples (Supplemental Fig. S1*C*), indicating sampling of the same proteins but presentation of different peptides within each sample. Next, we allocated the source proteins from each sample dataset into broad categories based on cellular compartments. The majority of source proteins in both the cell line and PE samples localized to the cytoplasm, followed by membrane and nuclear compartments (Fig. 1*C*).

### Investigating the Cancer-Associated Antigen Landscape in Mesothelioma

**Fig. 2:**
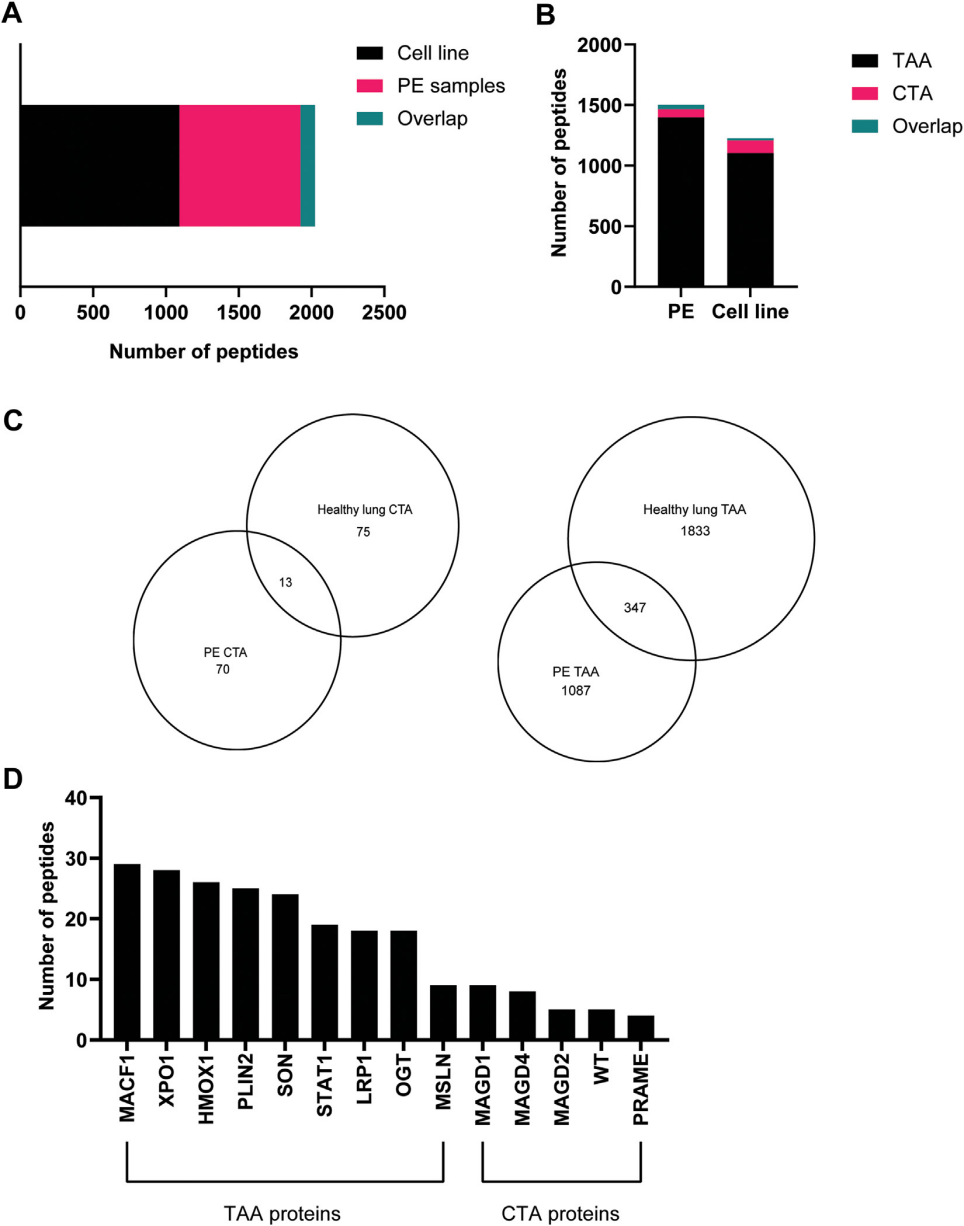
**An overview of the cancer-associated antigen-derived peptides identified in mesothelioma.***A*, Bar chart representing the total number of unique and overlapping cancer-associated peptides identified in patient-derived cell lines (*black*) and PE samples (*pink*). *B*, stacked bar chart representing the distribution of TAA and CTA peptides identified in PE samples (n = 4) and cell line (n = 1). *C*, Venn diagram showing mesothelioma-specific CTA and TAA peptides identified in PE samples after subtracting CTA and TAA peptides identified from healthy lungs. *D*, the total number of mesothelioma-specific peptides identified in PE samples from selected oncogenic proteins using the TANTIGEN database and CTDatabase.

The cell line was searched for mutational neopeptides identified using genomic analysis, however, none were detected in the immunopeptidome. In contrast, we identified a total of 2024 peptides from TAA and CTA proteins across the cell line (n = 1093) and four PE samples (n = 831) with 100 overlapping peptides identified between the cell line and PE samples (Fig. 2*A*, Supplemental data file S3). Briefly, TAA-derived peptides originating from oncogenes constituted the most prevalent antigen source in both the cell line and PE samples (Fig. 2*B*) followed by CTA peptides, which had similar numbers in both the sample subtypes (Fig. 2*B*).

To identify mesothelioma specific TAA and CTA peptides, we compared our data with healthy lung data from the HLA ligand atlas (also known as benign tissue atlas) (18). The HLA-class I data for 15 donors was searched using Peaks Online. We identified a total of 56,000 HLA class I peptides (including only 8–12mers). A total of 2268 TAA and CTA peptides were identified in the benign lung atlas data. When compared with PE sample, an overlap of 13 CTA peptides and 347 TAA peptides was found between the two datasets (Fig. 2*C*). For the cell line data, the overlap between CTA-derived peptides was smaller (12 common peptides) compared to shared TAA peptides (259 common peptides) (Supplemental Fig. S2, *A* and *B*).

The proteins that contributed to the highest number of TAA peptides were Microtubule-actin cross-linking factor 1 (MACF1) and DNA/RNA binding protein (SON) and Beta-catenin 1 (CTNB1) in both PE samples (Fig. 2*D*) and cell line (Supplemental Fig. S2*C*). MAGE D family of proteins contributed the highest numbers of CTA-derived peptides in both PE samples (Fig. 2*D*) and cell line (Supplemental Fig. S2*C*) along with Wilms Tumor 1 (WT1) protein. PE samples also had peptides coming from PRAME protein which was not found in healthy lung samples.

We were interested in identifying peptides coming from known biomarkers of mesothelioma. Across both the sample subtypes, we identified peptides coming from mesothelin (MSLN) and fibulin-3 (FBLN3) proteins. Especially in the patient-derived cell line, six peptides were identified from MSLN protein (Fig. 2*B*). This was of particular interest since previously published work by Sneddon *et al.* (12) showed high MSLN protein levels in the serum of the donor from whom the cell line was derived.

### Cysteinylated Peptides Dominate the PTM Peptide Repertoire in Mesothelioma Samples

Peptides carrying post-translational modifications are known to be loaded onto HLA molecules, thereby further diversifying the immunopeptidome. Such PTMs can change the charge, stereochemistry, and other properties of the bound peptide, potentially modulating their immunogenicity (26). Therefore, as a part of this study, we explored the PTM peptide repertoire of the mesothelioma cell line and PE samples. Around 27% and 6% of peptides identified in cell line and PE samples were modified respectively (Supplemental data file S4, Supplemental Fig. S3*A*).

**Fig. 3:**
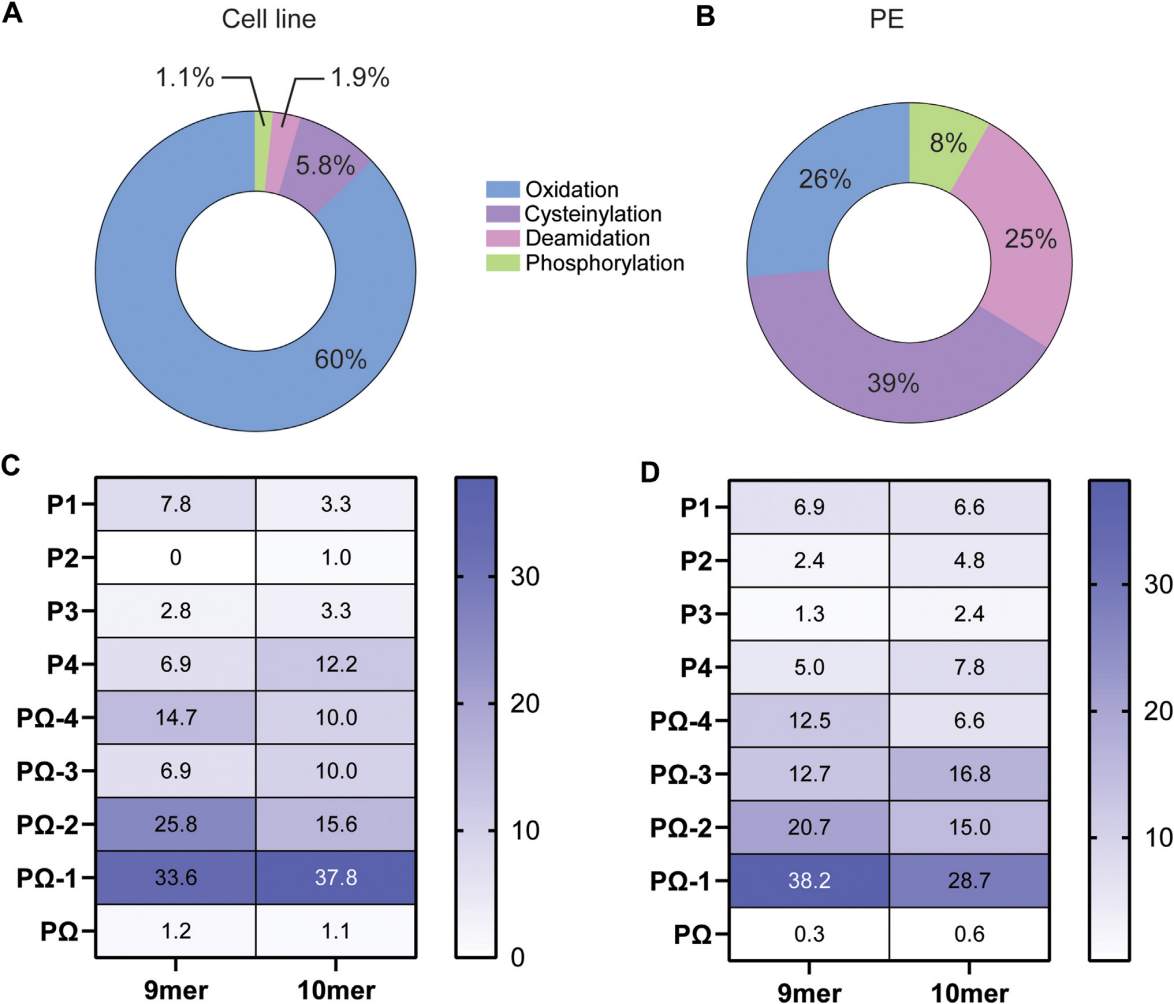
**Cysteinylated peptides are prevalent in the immunopeptidome of mesothelioma.** Pie chart representation of percentage contribution of different PTM peptides in (*A*) patient-derived cell line and (*B*) PE samples. Amino acid frequency analysis represented as a heat map to identify the preferred site for cysteinylation to occur in 9mer and 10mer peptides in (*C*) cell line and (*D*) PE samples. The gradient bar on the right represents the range of percentage/frequency of Cys at those positions. *Dark blue* represents higher frequency whilst *white* or *light blue* represents low frequency of Cys. PΩ stands for the last amino acid (AA) of the peptide (ninth AA for 9mer and 10th AA for 10mer peptide).

Further analysis of the cell line data revealed that the majority of these PTM peptides (60%) were oxidized at Met (M) which most likely arose during sample preparation (Fig. 3*A*). The most abundant modification after oxidized methionine was the cysteinylation of cysteine residues. Of note, cysteinylated peptides were the most predominant subset of PTM peptide ligands in PE samples with a lower proportion of peptides bearing oxidized methionine in these samples (Fig. 3*B*).

Similar to the unmodified peptide repertoire, the majority of cysteinylated peptides in both sample subsets were 9mers (Supplemental Fig. S3*B*). Further analysis of these peptides (including both 9mers and 10mers) showed that cysteinylation occurred preferentially at the solvent accessible residues of PΩ-1, PΩ-2 and PΩ-4 in both the cell line (Fig. 3*C*) and PE sample (Fig. 3*D*) wherein Ω refers to the amino acid at the C terminus. Based on binding affinity prediction of the native version of the peptides, it was found that in the cell line the majority of the cysteinylated peptides (66–70%) were likely presented by HLA B allotypes (Supplemental Fig. S3*C*). However, in PE samples there was no such consensus with different HLA-A, -B, and -C allotypes predicted to bind to the cysteinylated peptides (Supplemental Fig. S3*D*). Intrestingly, when compared to healthy lung data, only 2% of peptides were found to be cysteinylated. This highlights the mesothelioma specific nature of this PTM.

Additional PTMs of note in both cell line and PE samples included deamidation of Asp (N) and Glu (Q) residues and phosphorylation at Ser (S) and Thr (T) (Fig. 3, *A* and *B*). Approximately 54% and 37% of the deamidated Asp (N) containing peptides contained a consensus N-glycan site of NX(S/T) in PE and cell line samples respectively. These peptides are most likely derived from formerly glycosylated antigens (27). Interestingly, for the phosphopeptide repertoire, more than half of peptides were phosphorylated at Ser [(S) 78% in cell line and ∼72% in PE samples], with most of these Ser phosphorylation present at P4 residue (67% in cell line and 53% in PE samples) and containing the XpSPX consensus sequence (Supplemental Fig. S3*E*).

### Identification of Human Endogenous Retrovirus-Derived Peptide Antigens

**Fig. 4:**
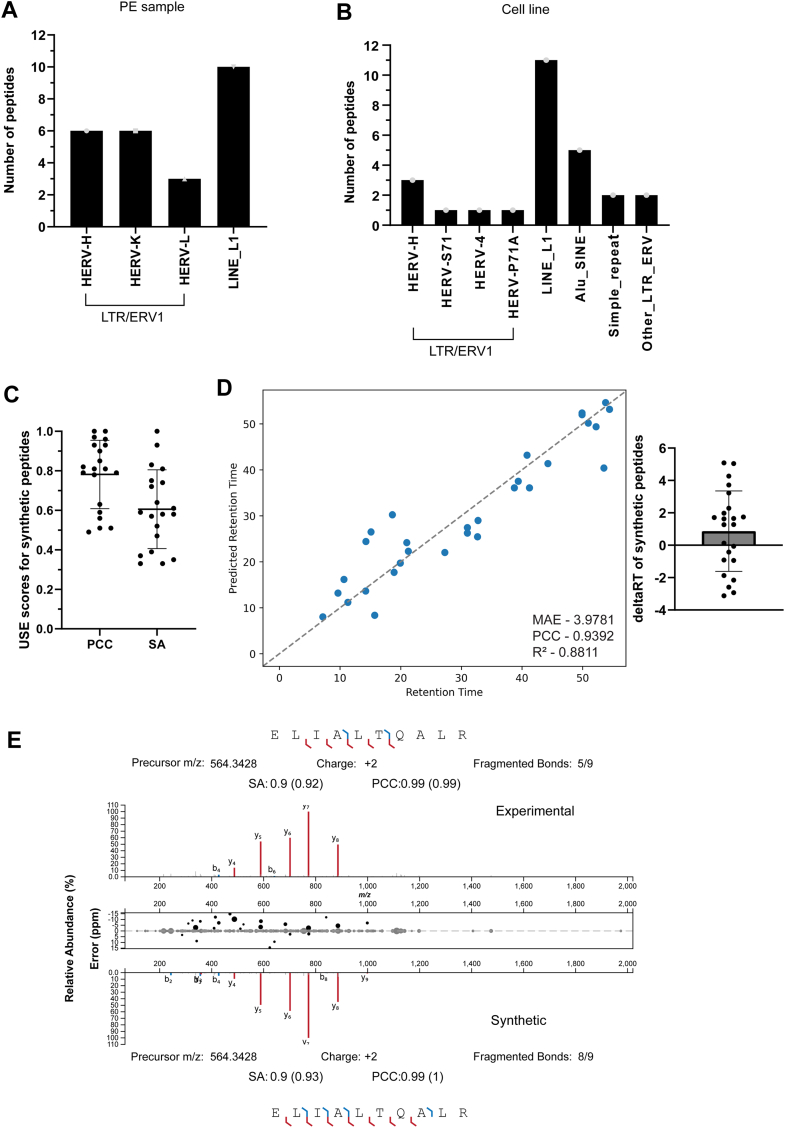
**Identification and validation of HERV peptides identified in PE samples.** Source proteins for the HERV peptides identified in the (*A*) PE samples and (*B*) cell line. The majority of the peptides in both samples come from the LTR/HERV1 and LINE L1 family of proteins. The similarity of 25 HERV peptides was assessed by USE. The MS/MS of experimental and synthetic peptide spectra was compared and the similarity between the two was calculated in the form of PCC and SA scores plotted in (*C*) a bar graph depicting the mean ± SD of PCC and SA scores for 20/24 peptides validated using this approach. *D*, scatter plot depicting linear correlation model of predicted vs observed RT for the HERV peptides. RT prediction model was trained on canonical peptides. The MAE, PCC, and R^2^ values that show how the correlation is mentioned at the *bottom right*. The RT values of the synthetic peptides are represented in the bar chart with mean ± SD. Twenty-one peptides were validated using a synthetic peptide approach with an average RT difference of 0.87 min ± 2.47 min. *E*, an example of a HERV peptide with high correlation between experimental (on *top*) and synthetic (on *bottom*) peptide.

There is strong evidence that different classes of HERVs can be reactivated in cancer cells, leading to the expression of viral-like proteins that may act as potential antigens for anti-tumor immune responses (28). Using the gEVE database a total of 54 unique HERV-derived peptides were identified across the samples with 29 and 25 peptides identified in cell line and PE samples respectively (Supplemental data file S5). On further investigation of the PE sample, we found that around half of the peptides originated from the LTR/ERV1 family and the other half from the LINE L1 family (Fig. 4*A*). Within the LTR family, six peptides were derived from both HERV-H and -K families. In the cell line, 30% of the peptides were derived from the LTR/ERV1 family, 42% were derived from the LINE L1 family, and the remaining were derived from the ALU/SINE family of HERVs (Fig. 4*B*).

To confirm the identification of the HERV peptides identified in PE samples we compared the MS/MS of experimental and synthetic peptide spectra. The similarity between the two was assessed using both PCC and SA scores. Based on these criteria, 20/24 HERV peptides were validated with an average PCC score of 0.78 ± 0.173 and an average SA score of 0.61 ± 0.199 (Fig. 4*C*). Furthermore, we performed RT prediction for the HERV peptides identified in PE samples using DeepLC. Canonical peptides were used to train the model which in turn was used to predict the RT of the HERV peptides. There was a good correlation between predicted and observed RT, as evident from MAE of 3.9781 and PCC score of 0.9392. Using DeepLC, 20/25 peptides were validated (Fig. 4*D*). At the same time, we also verified RT using synthetic peptides. In total, 21/25 peptides were validated with an average RT difference of 0.87 min ± 2.47 min (total gradient length of 58 min). An example of a validated HERV peptide (ELIALTQALR) is shown in Figure 4*E* (made with USE) with a PCC score of 1 and SA score of 0.93 (considering bottom spectra contained in top spectra). The validated spectra of HERV peptides identified in PE samples can be found in Supplemental Fig. S5.

The HERV peptides identified in the cell line were validated using spectra predicted by PROSIT. Using PCC (average score of 0.89 ± 0.09) and SA score (average score of 0.77 ± 0.12) 25/28 peptides were validated (Supplemental Fig. S4*A*). DeepLC was used in this instance as well to predict the RT of the peptides. There was a good correlation between predicted and observed RT, as evident from the MAE of 8.3129 PCC score of 0.9145, and R^2^ of 0.8091. 19/29 peptides were validated using this approach (Supplemental Fig. S4*B*). Although the scores are lower compared to the HERV peptides identified in PE samples, they are still within acceptable limits. An example of one of the validated spectra for HERV peptide SSLQPLPPR is shown in Supplemental Fig. S4*C*. The validated spectra of HERV peptides identified in cell line can be found in Supplemental Fig. S6.

Furthermore, to identify mesothelioma-specific HERV peptides we compared our data to HERV peptides identified in lung samples of benign tissue atlas. A total of 66 peptides were found. The HERV peptides identified in healthy lungs came from a much more diverse set of source proteins wherein ∼48% of peptides belonged to the LINE L1 family. Peptides were interspersed across different LTR/HERV1 families such as HERV-E, -H, -K AND -L (Supplemental Fig. S4*D*). There was an overlap of only three and two peptides between healthy lung HERVs with cell line and PE samples respectively (Supplemental Fig. S4*E*). The overlapping HERVs came from LINE L1 family of protein and SINE/Alu family of HERVs.

## Discussion

Mesothelioma is a rare but aggressive cancer, the incidence of which is rising worldwide (5). Most studies in the field have focused on genomics and transcriptomics to understand the onset of the disease and its pathogenesis (29, 30, 31, 32) with fewer studies focusing on proteomics of either plasma or the secretome of mesothelioma cells (33, 34). Hence further research aimed at understanding the biology of the disease is necessary since it may help in the development of novel therapeutic approaches. A promising modality of treatment would combine first line treatment with immunotherapy, particularly T cell based or targeted therapies. However, there is a lack of well characterized T cell targets in mesothelioma with only a handful reported in the last few years (35). Notably, it has been demonstrated that infiltration of the tumor by T cells is associated with better prognosis (36). It has been demonstrated, that direct analysis of the immunopeptidome of either the primary tumor or patient-derived cell lines can aid in identification of not only naturally processed and presented peptides but also of highly relevant tumor-associated antigen-derived peptides and neoepitopes (37, 38). Specifically, it has been shown in mesothelioma that dendritic cells fed with apoptotic mesothelioma cells can activate both CD8+ and CD4+ T cells resulting in tumor clearance (39, 40), highlighting the existence of potent tumor antigens. Moreover, CTL responses have also been demonstrated against peptides from known mesothelioma biomarker proteins such as mesothelin (41) and Wilms’ Tumor oncoprotein (42). Hence utilizing an immunopeptidomics approach to unravel the HLA class I peptide repertoire of patient derived mesothelioma cell line and pleural effusions is an effective tool to identify potential peptide antigens of great benefit. The direct identification of such pHLA isolated from the surface of the tumor circumvents issues surrounding the prediction and imputation of presentation of tumor-associated peptide antigens based on genomic and transcriptomic analysis (37, 38).

In this study, we identified a total of 54,279 non-redundant HLA class I bound peptides (8mers to 12mers only) from a patient-derived cell line and four unrelated PE samples. Of note, a predominance of HLA-B and -C binding peptides was observed in PE samples by both prediction algorithms used in the study. This is distinct to what has been observed in other tumors wherein peptide presentation by HLA-B and -C alleles typically predominate after exposure to IFNγ (43). As reported previously, mesothelioma is one of the few tumor subtypes which have a prevalent interferon-stimulated gene (ISG) signature (44) where cells have been found to maintain activation of IFN type 1 signaling (11, 45). HLA-B allotypes are typically the most inducible of the HLA loci since they have two interferon response elements upstream instead of one found upstream of HLA-A and -C (46). Therefore, increased ISG signaling may predispose mesothelioma towards increased HLA-B and -C expression, consistent with our immunopeptidomics analysis.

We compared our data with two previous studies which looked at Mesothelioma and lung adenocarcinoma. First, the recently published data from Chiaro *et al.* (47) including mesothelioma cell lines and patient samples. We found an overlap of only 3041 peptides (4.5%) between the two datasets (47). Of these, 173 peptides overlapped with peptides identified using TANTIGEN and CTdatabase. Second, a comparison with a study by Khazan-Kost *et al* (48) that explored the immunopeptidome of lung adenocarcinoma in both pleural effusion and primary cell lines. At the immunopeptidome level, there was an overlap of 5% (1649) reflecting this disparate HLA allotypes expressed by the different samples, but at the source protein level, the overlap was ∼16% (1874) between the samples. Most of these common source proteins were involved in RNA metabolism, cell cycle, DNA damage response, and cellular response to stress. However, we identified 53 shared TAAs between the two datasets.

Mesothelioma has low TMB, the samples included in this study only had 28 to 41 mutations. No mutational neopeptides were found in our immunopeptidomic datasets. To identify peptides that were unique to mesothelioma, we compared our data to the HLA class I peptides identified in benign lung atlas (from 15 healthy donors). A total of 170 CTA and 1951 TAA peptides unique to cell line and PE samples were identified. The proteins that were unique to mesothelioma samples (cell line and PE samples) were MAGED4, PRAME, WT1, ACRBP, EPHA2 and SOX11. Of particular interest were peptides derived from mesothelioma biomarkers including mesothelin (49). A total of 13 MSLN-derived peptides were identified, with one of the peptides (LLGPHVEGL) likely to be restricted to HLA A∗02:05 based on NETMHC predictions and the HLA genotype of the patient. Of note, this peptide is a shorter version of peptide KLLGPHVEGL from mesothelin which was previously shown to be a target of tumoricidal T cells restricted to HLA A2 (41). Other peptides of interest include the two peptides derived from WT tumor antigen (QRNMTKLQL and ASSGQARMF) which has previously been reported to elicit T cell response respectively in healthy A2 donors (42).

Post-translational modifications (PTMs) diversify the immunopeptidome and in some circumstances these peptides can be considered as neoepitopes, particularly in cancers where perturbations in signaling or changes in microenvironment can result in an increase or aberrant formation of PTMs (50, 51, 52). In this study, there were several interesting findings. First, we detected a higher percentage of cysteinylated peptides in PE samples (25%) compared to the mesothelioma cell line (5.8%). This was quite surprising considering PE samples were collected directly from patients and analyzed *ex vivo*. Second, cysteinylation is not a frequently observed PTM in other cancer immunopeptidomes. Investigation of other published datasets (43) demonstrated that cysteinylated peptides were only detected in cell lines treated with IFNγ and even in this state of stimulation the percentage was low (0.7% - 0.8% of all PTMs). Therefore, it’s highly unlikely that these modified peptides are an artefact of free cysteine present in tissue culture medium. Furthermore, when compared to healthy lung data, only 2% of peptides were found to be cysteinylated. This unusually high number of cysteinylated peptides may be attributed to production of reactive oxygen species (ROS) resulting from prolonged exposure to deposited asbestos (53) and although the role of cysteinylated peptides needs to be explored in the context of mesothelioma, previous studies have shown that cysteinylation does not have a negative impact on pMHC binding and immunogenicity (54, 55).

While most retroviral elements are considered "junk DNA" and have lost their ability to produce infectious viruses, there is strong evidence that they may play a role in human diseases, in particular cancer (9). Retroviral elements can be reactivated in cancer cells, leading to the expression of viral-like proteins that may act as potential antigens, triggering an immune response in the host against the cancer cells (28, 56). Sun *et al.* (11) reported an upregulation of both LTRs and LINEs in mesothelioma patients. In this study, we also see a predominance of peptides derived from these two families of HERVs in both PE and cell line samples. Within the LTR family of HERVs, Sun *et al.* have reported upregulation of ERV1 and ERVH subtypes. Specifically, in ERV1 they saw upregulation of LTR48B, LTR74, and LTR6 HERVs. We did not identify any peptides from these HERVs. However, we identified peptides derived from the ERV-H family in both cell line and PE samples. Similar to Sun *et al.*, we saw ERV-H-derived peptides coming from a Chromosome three region alongside other regions of interest including Chromosome 2, 7, 12, and 16. These chromosomes are of special interest because they code for proteins that play a crucial role in mesothelioma including ISG genes such as RSAD2, IFI44, and OAS2 (Chr2, Chr1, and Chr 12 respectively) or DNA damage-related genes CHEK2 and MSH2 (Chr 22 and Chr 2) and MSLN on Chr 16.

## Conclusion

As a part of this study, we have identified a variety of peptides belonging to different classes of cancer-specific antigens that have potential in mesothelioma immunotherapy. These included peptides derived from known immunogenic cancer testis and tumor-associated antigens, a striking proportion of cysteinylated peptide antigens, and several peptides derived from the transactivation of HERVs including peptides derived from LTRs and LINEs. Our data suggest that refocusing immunotherapy efforts on such antigens may be of great value in the treatment of mesothelioma and related tumors.

## Data Availability

The four pleural effusion immunopeptidomics data was deposited to data to the ProteomeXchange Consortium via the PRIDE partner repository. The PE samples were submitted with the dataset identifier PXD054498 (Project DOI: 10.6019/PXD054498) as.d files, mzIdentML and mgf files exported by PEAKS 12. The HERV database used for the search was uploaded with the raw files.

The mesothelioma cell line data was submitted with the dataset identifier PXD037845 (Project DOI: 10.6019/PXD037845) as.raw files, mzIdentML and mgf files exported by PEAKS X Pro.

Genomics data is available in the NCBI SRA repository, [https://www.ncbi.nlm.nih.gov/bioproject/PRJNA419420].

## Supporting information

This article contains supporting information.

## Conflict of Interests

The authors declare that they have no conflicts of interest with the contents of this article.

## Supplementary Data

Supplementary datafile 1

Supplementary datafile 2

Supplementary datafile 3

Supplementary datafile 4

Supplementary datafile 5
