# Supplementary material for: Multiple Classes of Antigen Contribute to the Antigenic Landscape of Mesothelioma
Source: Mol Cell Proteomics. 2025 Feb 5;24(3):100925. doi: 10.1016/j.mcpro.2025.100925 (PMC11929013; doi:10.1016/j.mcpro.2025.100925)

**Multiple classes of antigen contribute to the antigenic landscape of mesothelioma**

**Kirti Pandey^1^, Pouya Faridi^2^, Rochelle Ayala^1^, Y.C. Gary Lee^3^, Ebony Rouse^3^, Sanjay S.G. Krishna^1^, Ian Dick^3^, Alec Redwood^3^, Bruce Robinson^3^, Jenette Creaney^3^ * Anthony W Purcell^1^***

**Supplementary Materials:** The following supporting information can be downloaded at: www.mdpi.com, Figure S1: Mesothelioma landscape is dominated by nonameric peptides restricted by HLA B and C alleles.; Figure S2: Shared peptide repertoire between PE and cell line samples, Figure S3: Cysteinylated peptide repertoire of mesothelioma cell line, Figure S4: Identification and validation of HERV peptides identified in cell line

**Figure S1**

**
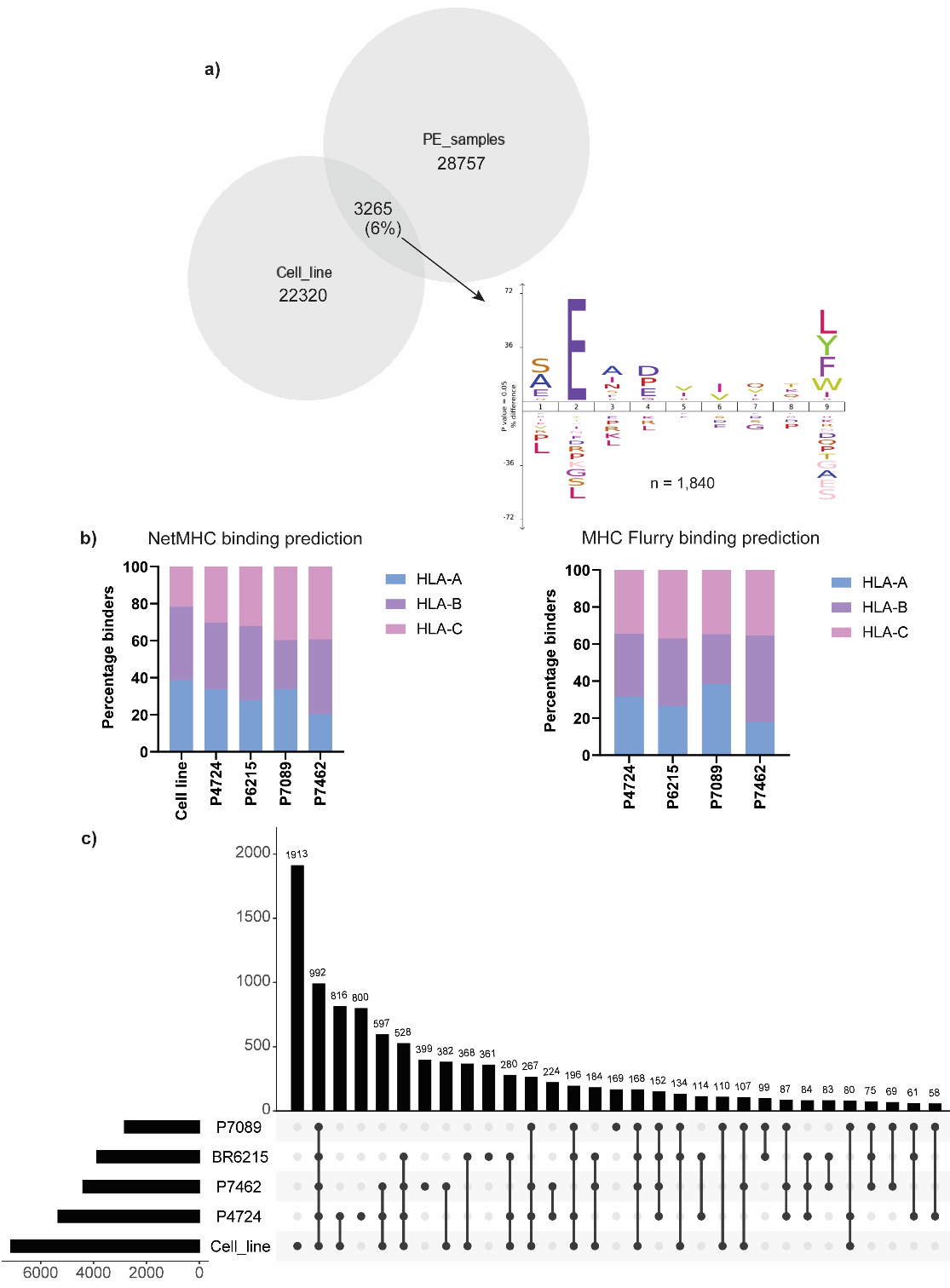
**

**Supplementary Figure 1 –** **Mesothelioma landscape is dominated by nonameric peptides restricted by HLA B and C alleles. a)** Venn diagram showing overlapping peptides between PE and cell line samples. Majority of the overlapping peptides were restricted to 2 shared HLA allotypes (HLA - B*44:03 and -C*07:01). **b)** The binding affinity of the peptides was predicted using NetMHC 4.0 and MHCFlurry 2.0. Bar chart representing predicted binding affinity of 9mers restricted to HLA A, B and C alleles. c**)** Upset plot depicting unique and overlapping source proteins between cell line and PE samples.

**Figure S2**

**
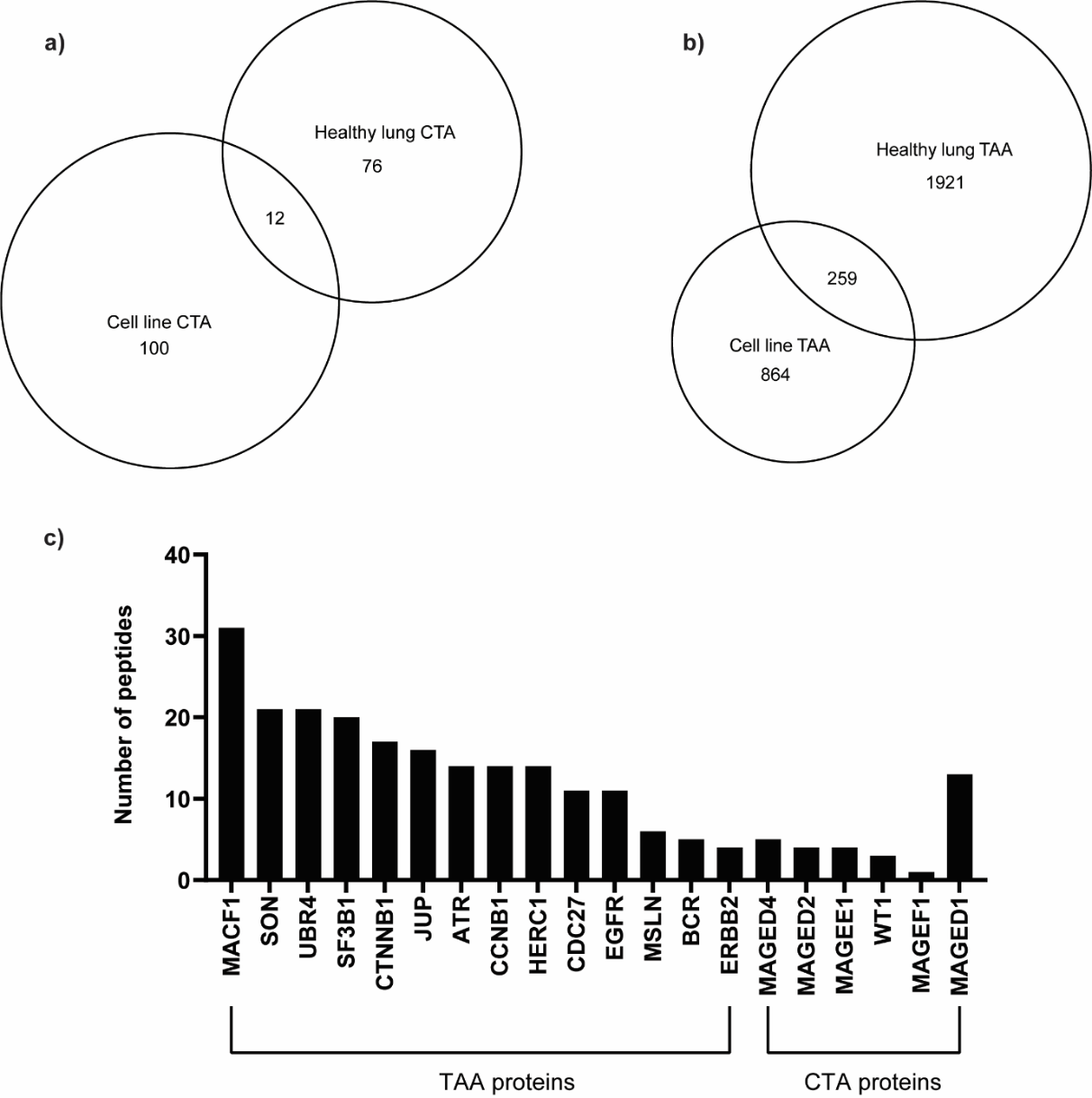
**

**Supplementary Figure 2 –** **Shared CTA and TAA repertoire between cell line and healthy lung samples**

Venn diagram depicting shared and unique peptides identified between cell line and healthy lung **a)** Cancer testis antigen (CTA)-derived peptides and **b)** Tumour associated antigen (TAA)-derived peptides. **c)** Total number of mesothelioma specific peptides identified in cell line sample from selected oncogenic proteins using the TANTIGEN database and CTDatabase.

**Figure S3**

**
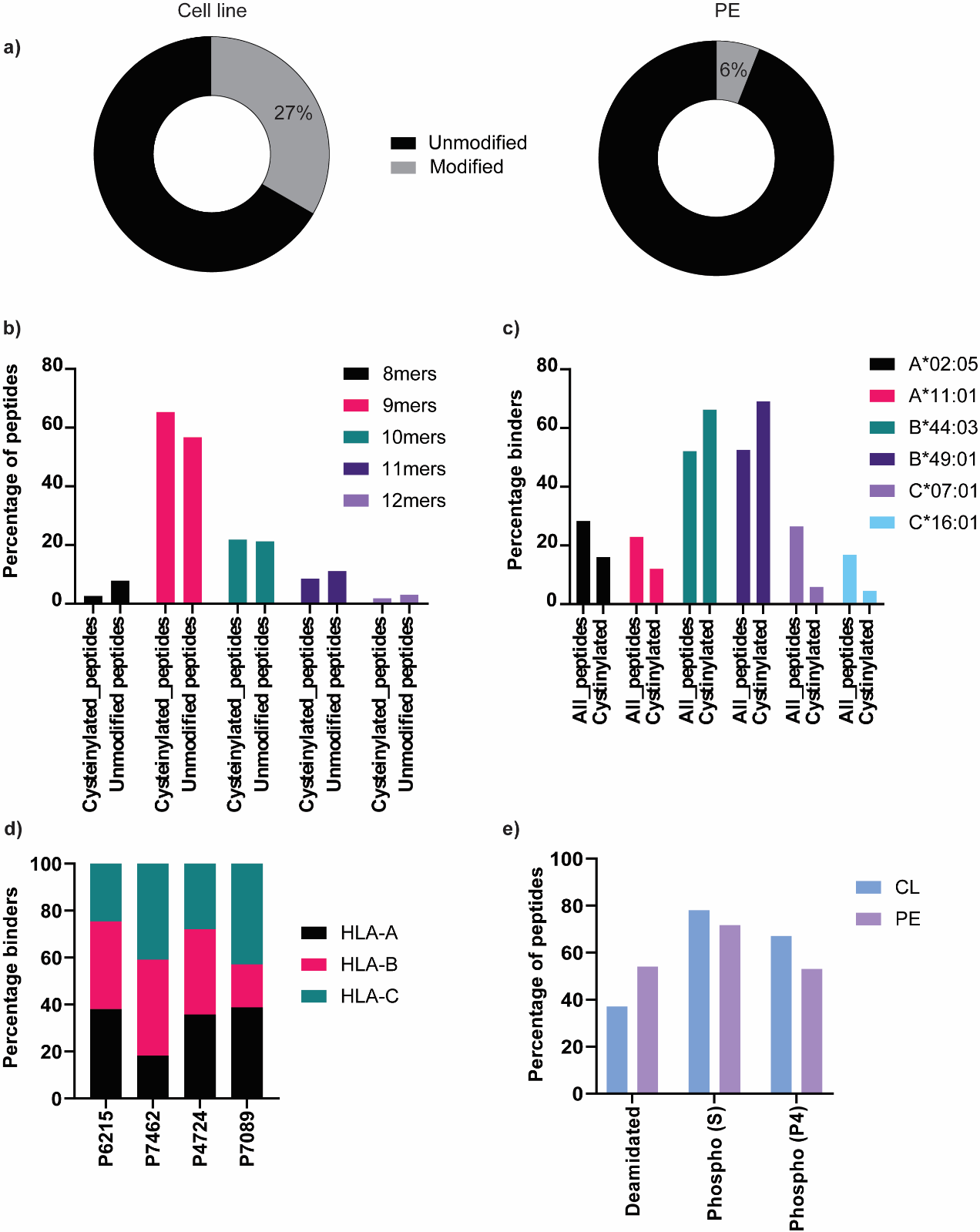
**

**Supplementary Figure 3 -** **PTM peptide repertoire of mesothelioma cell line and PE samples.**

Percentage of unmodified and modified peptides identified in **ai)** Cell line sample and **aii)** PE samples. **b)** Cysteinylated peptides identified in the study follow canonical class I length distribution similar to unmodified peptides. **c)** Majority of the cysteinylated peptides identified in cell line are restricted to HLA B alleles (B*44:03 and B*49:01) based on NetMHC prediction of unmodified peptides. **d)** Bar chart representing predicted binding affinity of unmodified 9mers restricted to HLA A, B and C alleles. across PE samples. P7462 was dominated by peptides that bind to either HLA B alleles. **e)** Bar chart representing the percentage of Asp (N) deamidated and Ser (S) phosphorylated peptides identified in cell line and PE samples.

**Figure S4**


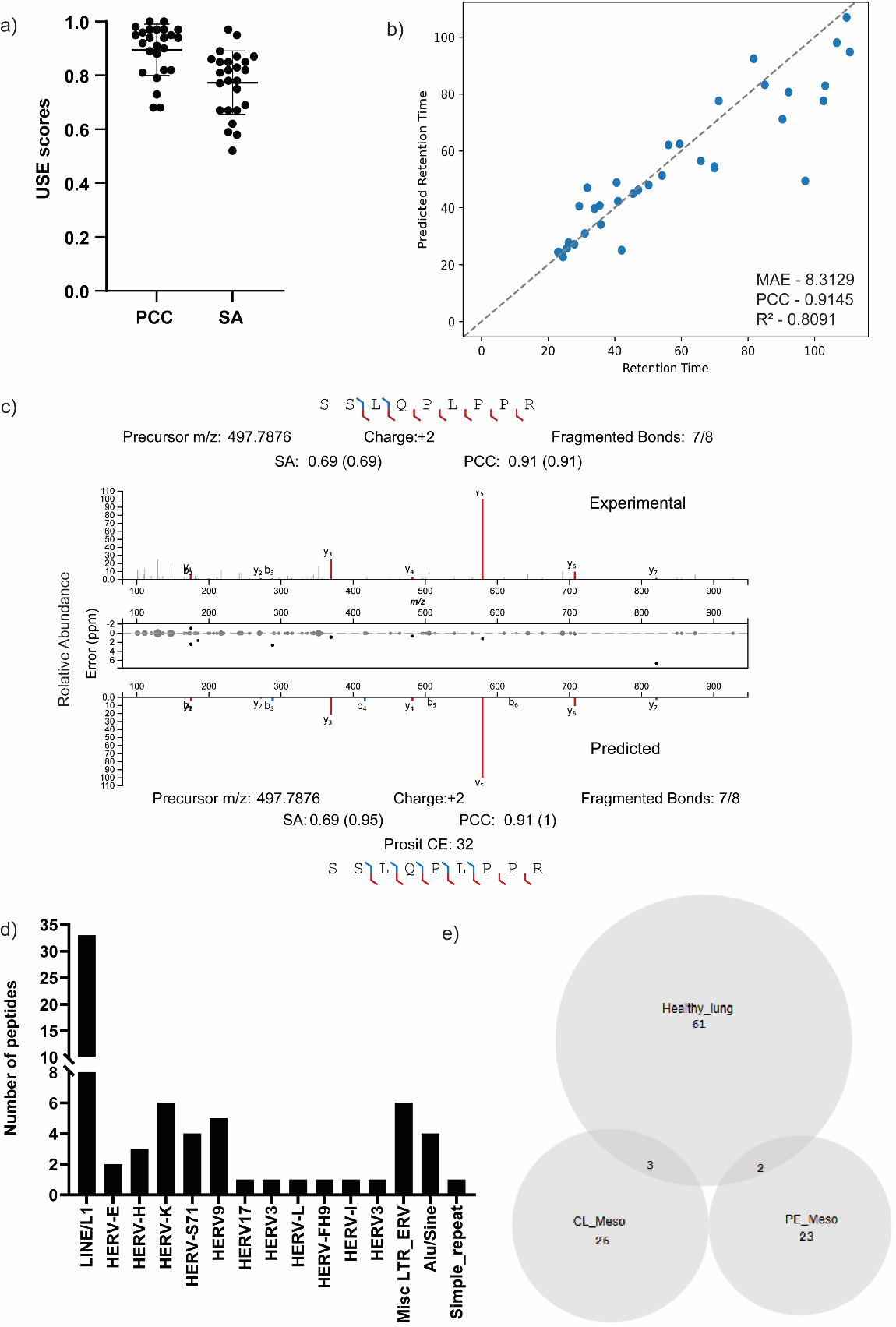


**Supplementary Figure 4 –** **Identification and validation of HERV peptides identified in cell line**

**a)** The similarity of 29 HERV peptides was assessed by USE. The MS/MS of experimental peptide was compared against predicted MS/MS spectra, generated by PROSIT (HCD 2020 model). The similarity between the two was calculated in the form of **a)** PCC and SA scores and 25/28 peptides weer validated **b)** Scatter plot depicting linear correlation model of predicted vs observed RT for the HERV peptides. RT prediction model was trained on canonical peptides. The MAE, PCC and R2 which show how well the correlation is, is mentioned at the bottom right.

**c)** An example of a HERV peptide with high correlation between experimental (top) and predicted (bottom) peptide. **d)** Source proteins for the HERV peptides identified in the healthy lung sample. Majority of the peptides in both samples come from LTR/HERV1 and LINE L1 family of proteins. **e)** Venn diagram depicting overlap between HERV peptides identified in cell line (CL_meso), PE samples (PE_Meso) and healthy lung.

**Figre S5 Mirror plots for HERV peptides identified in PE samples.**


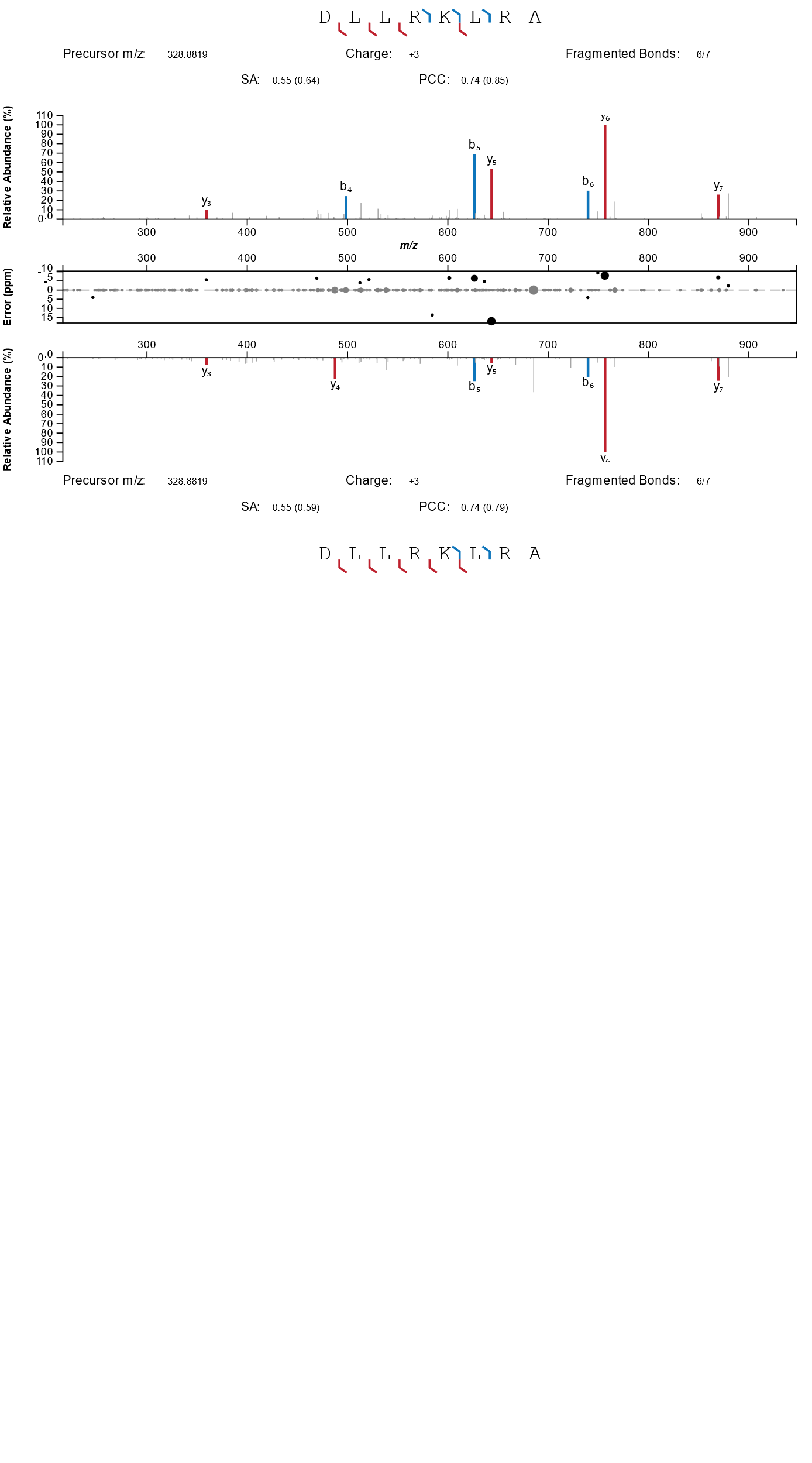

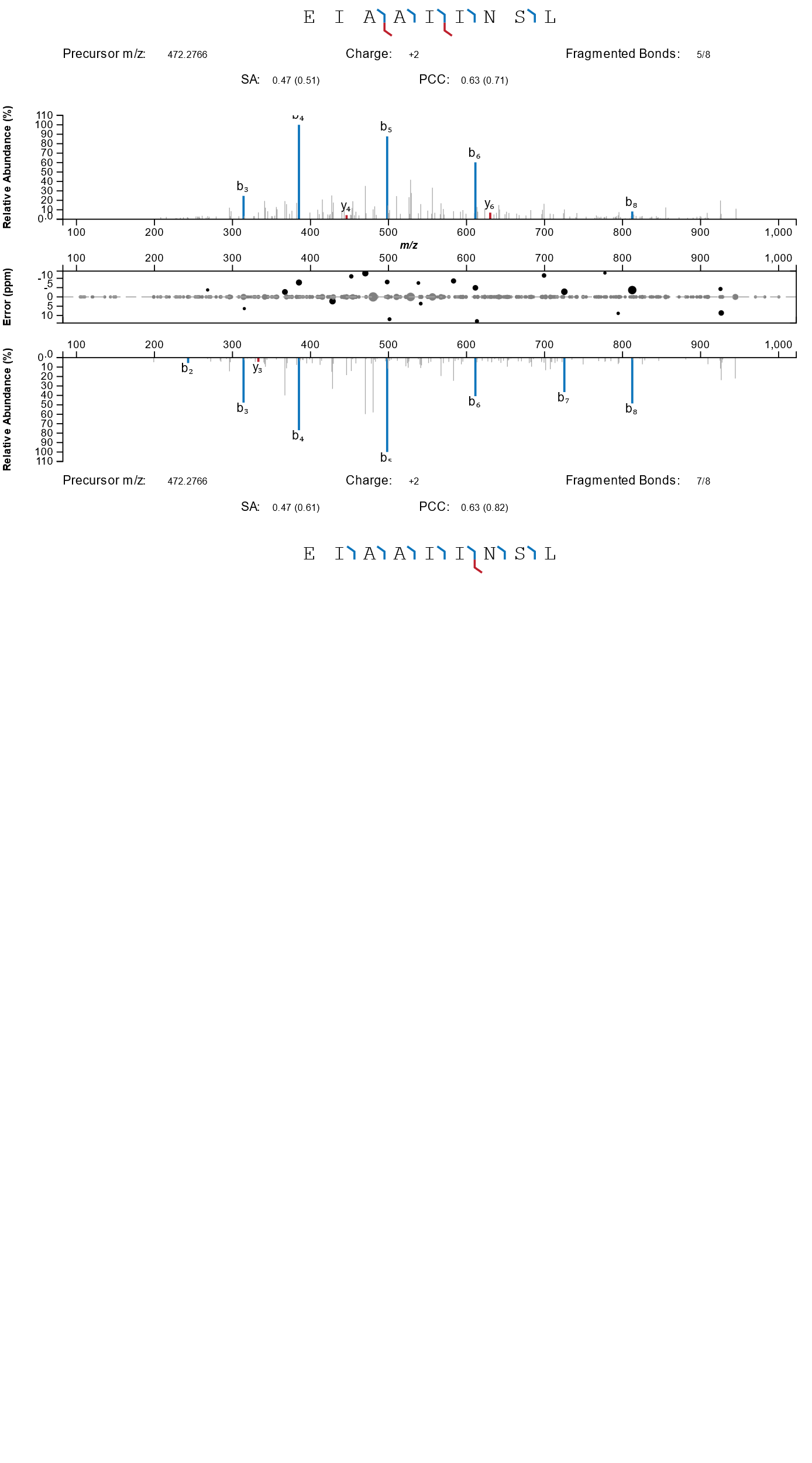

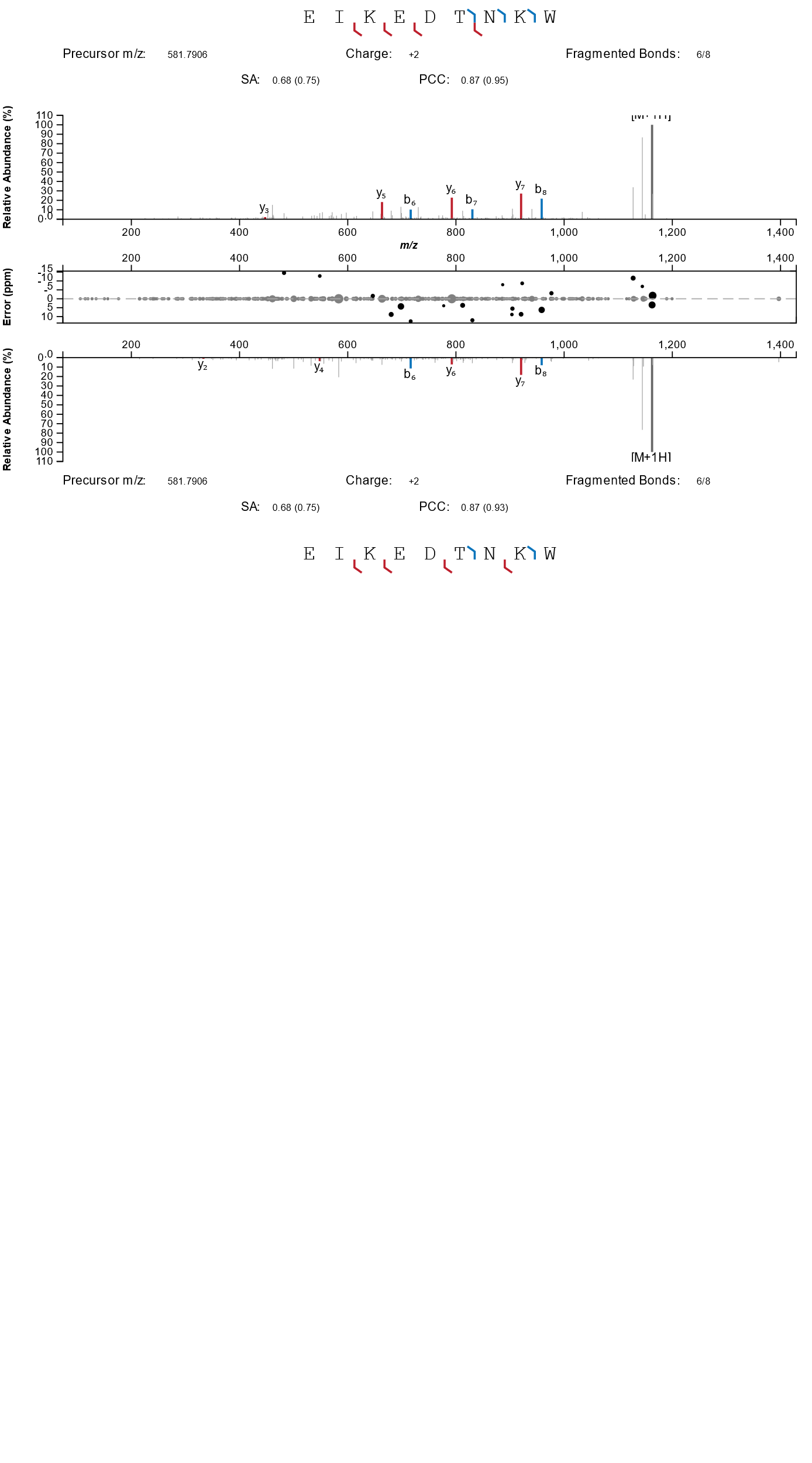

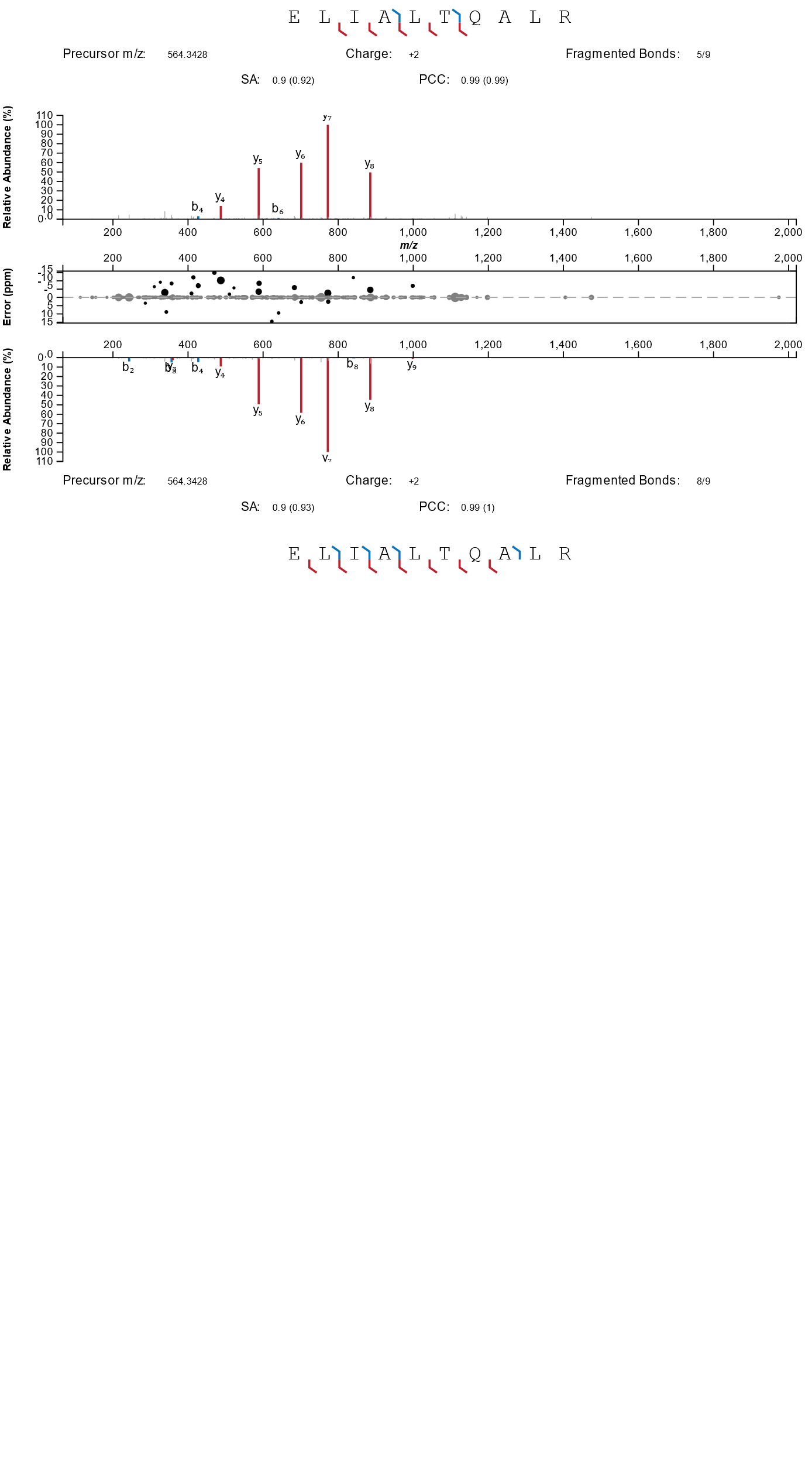

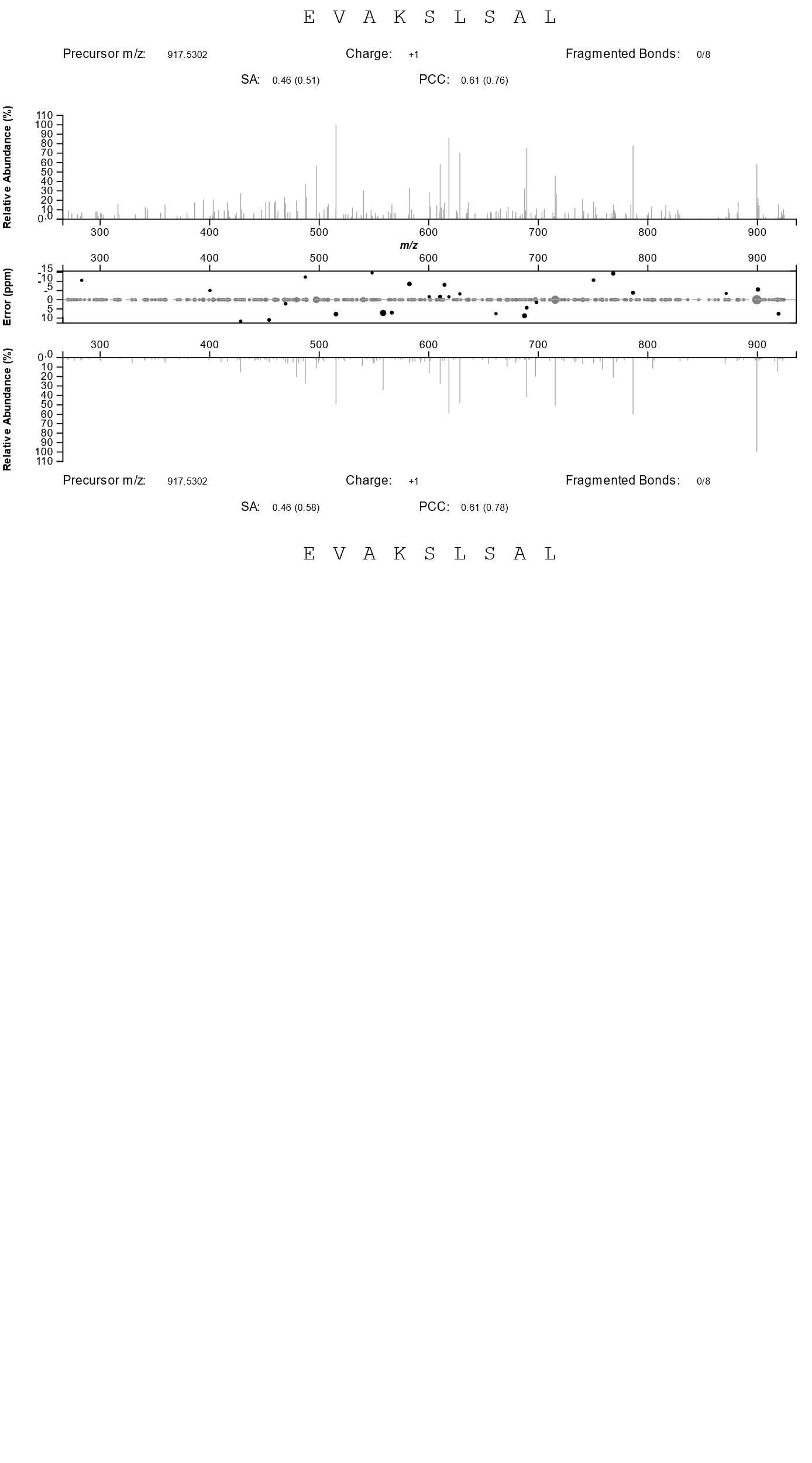

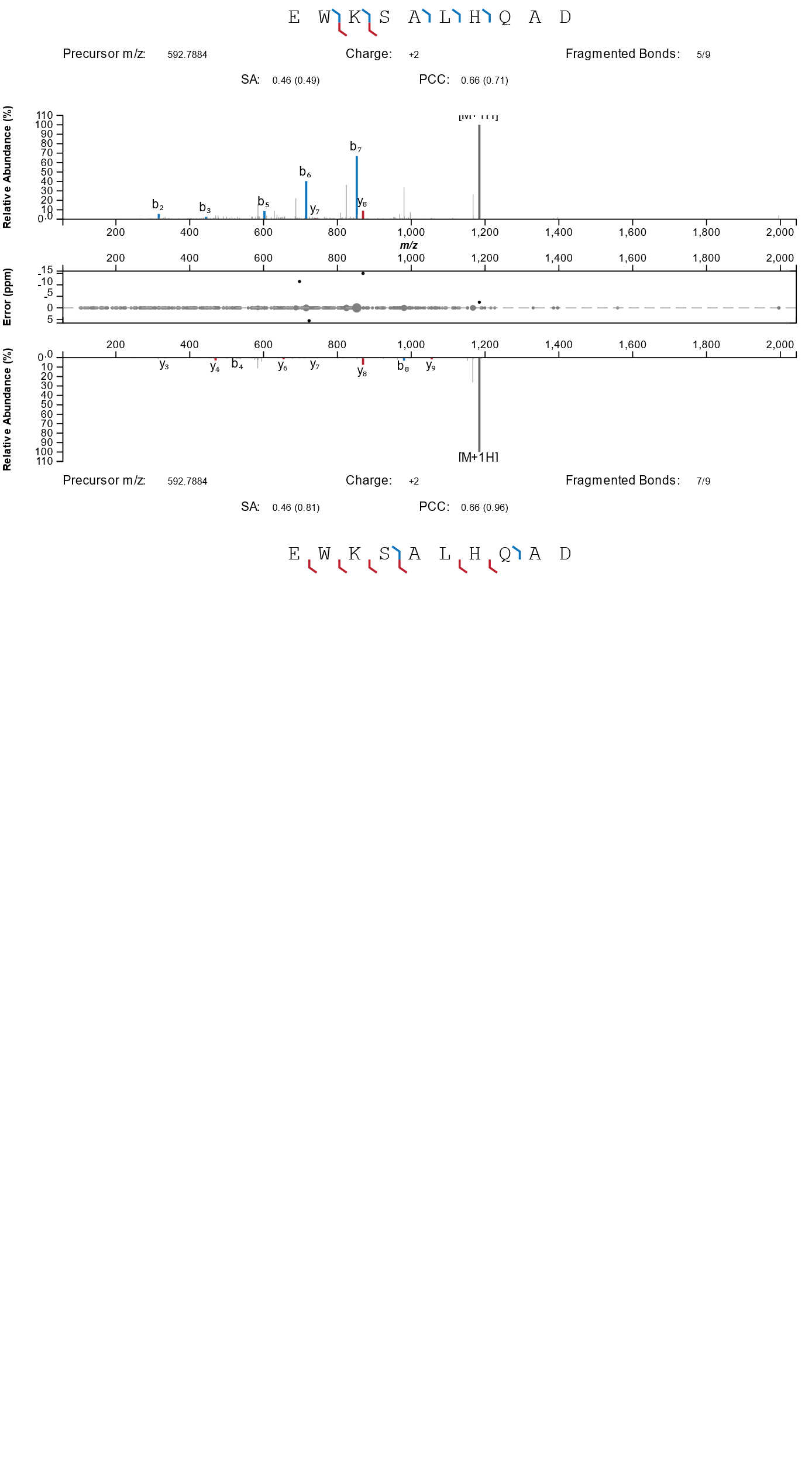

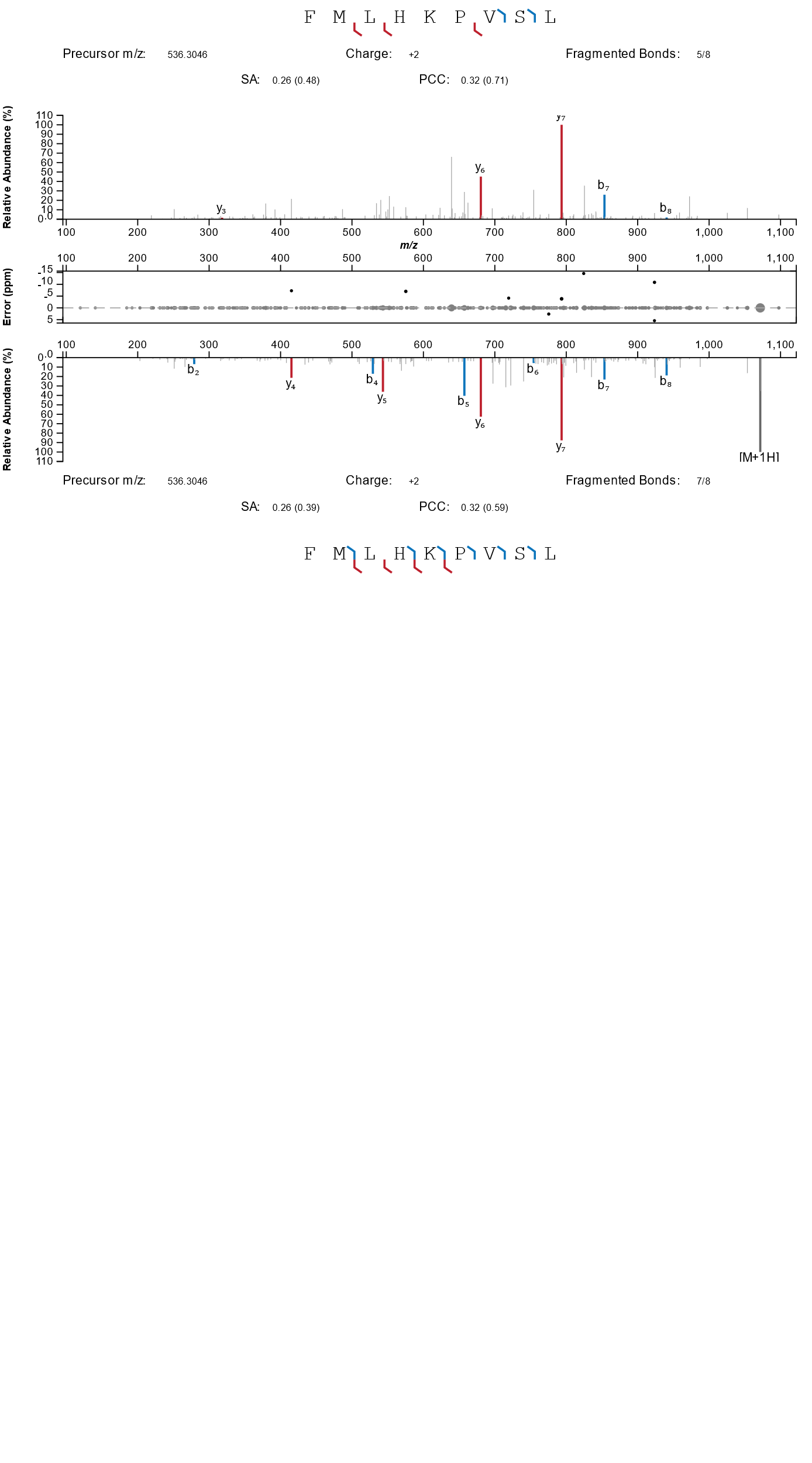

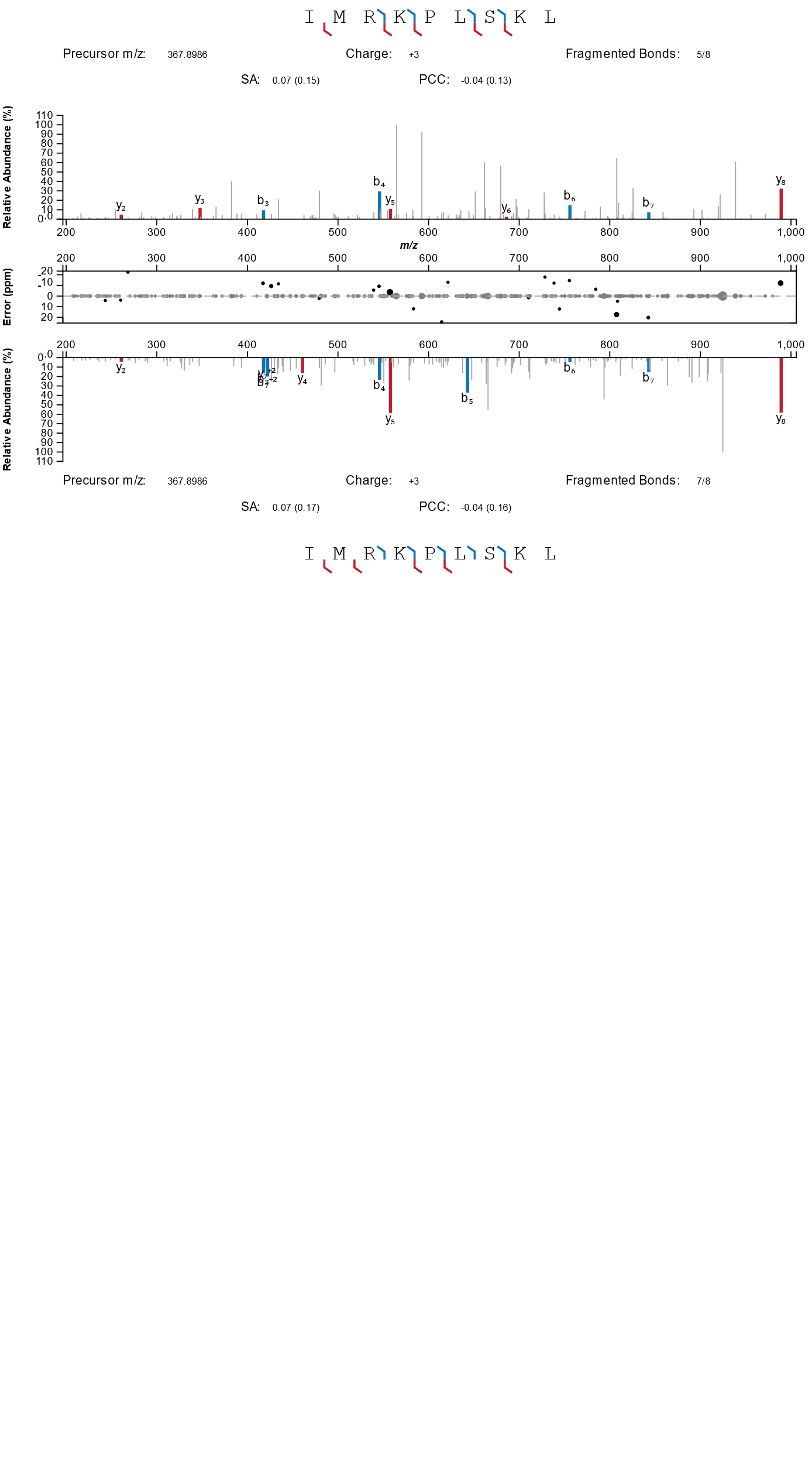

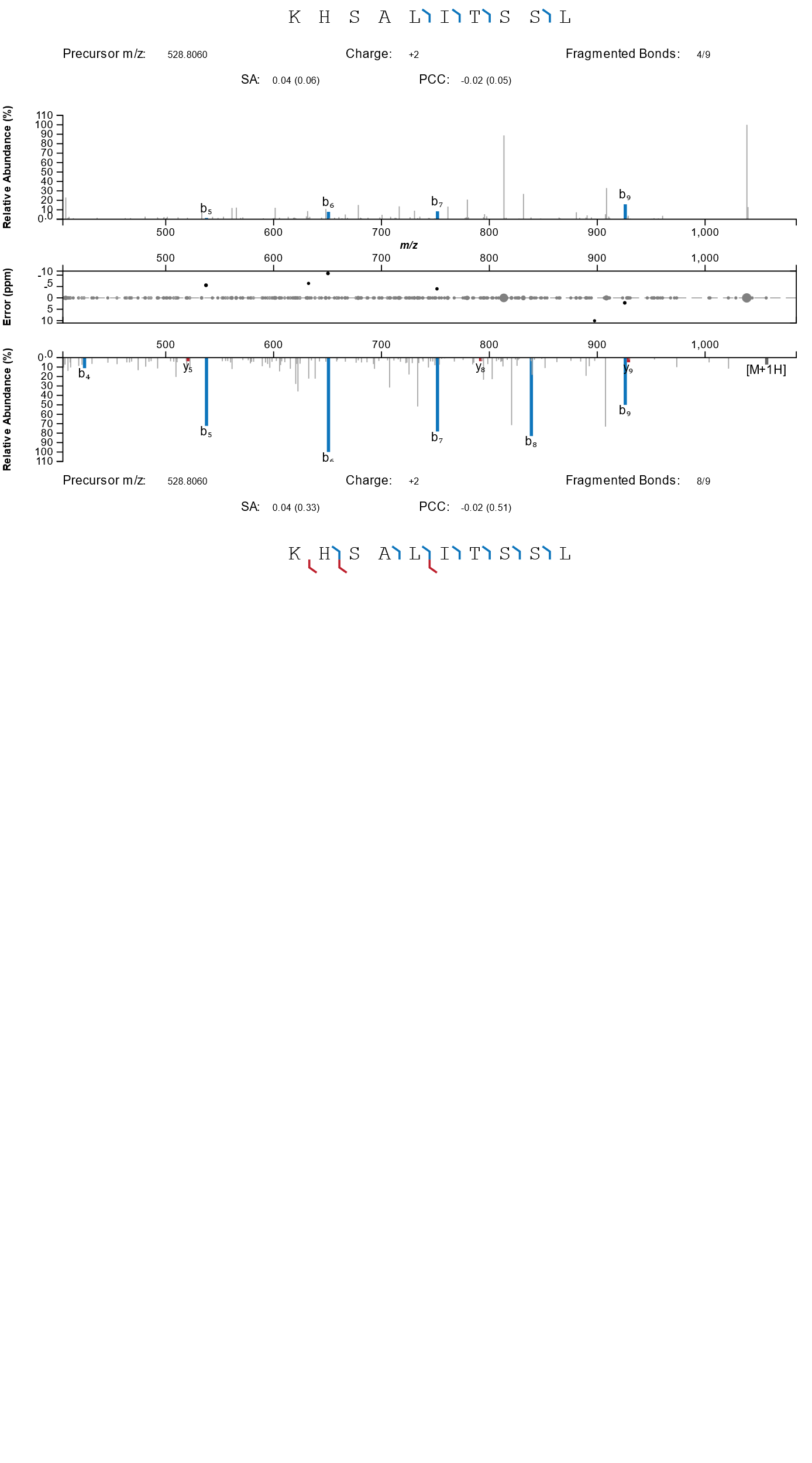

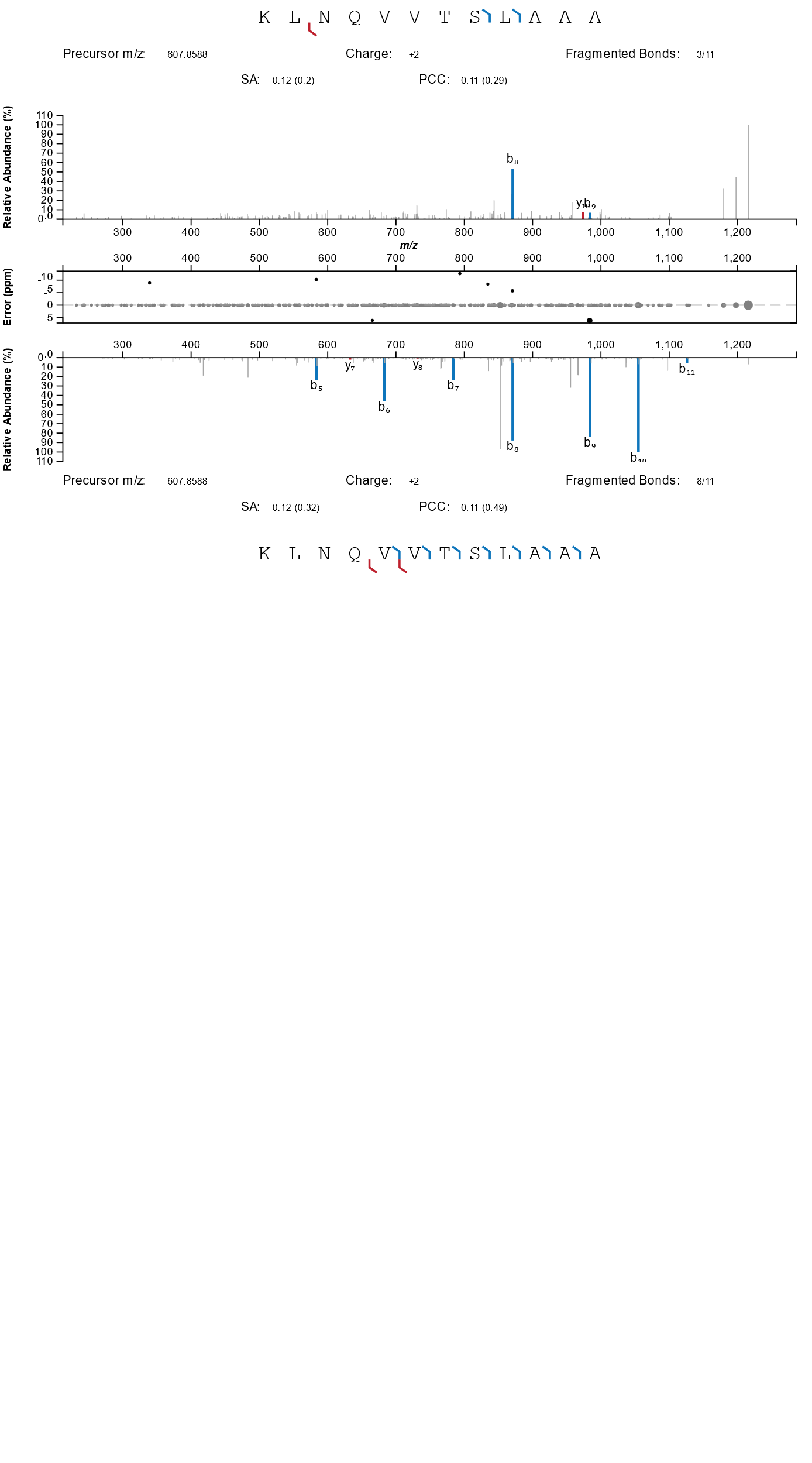

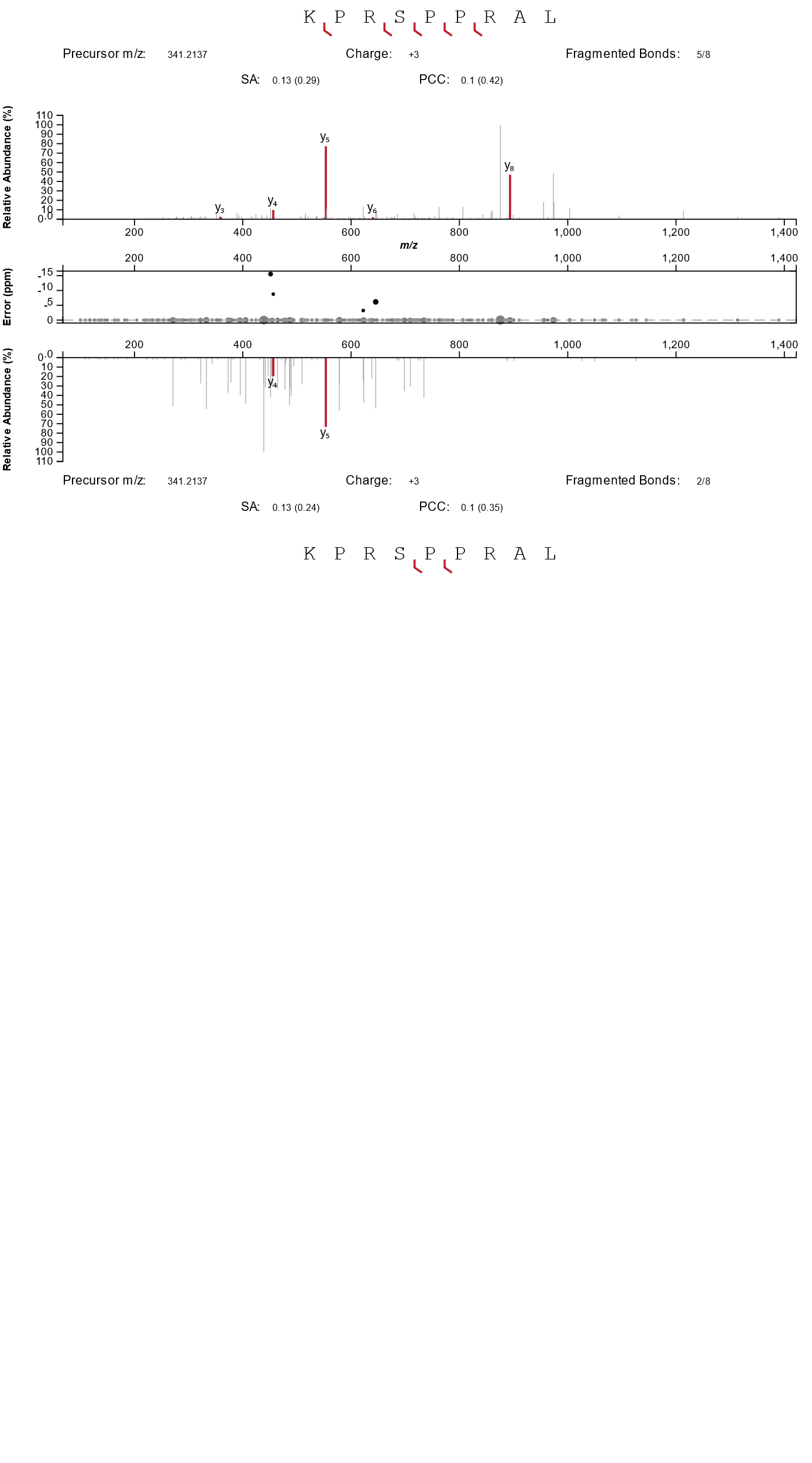

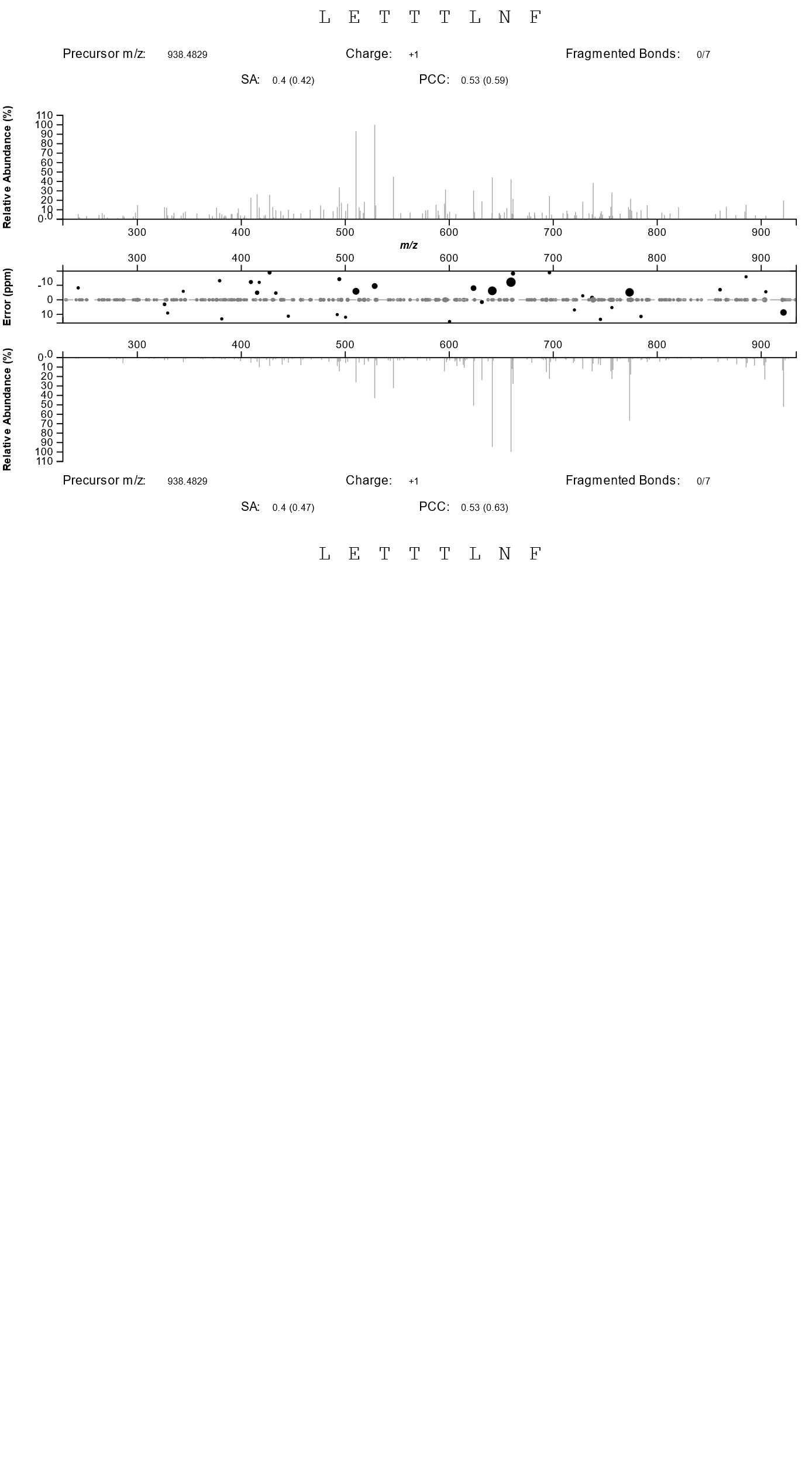

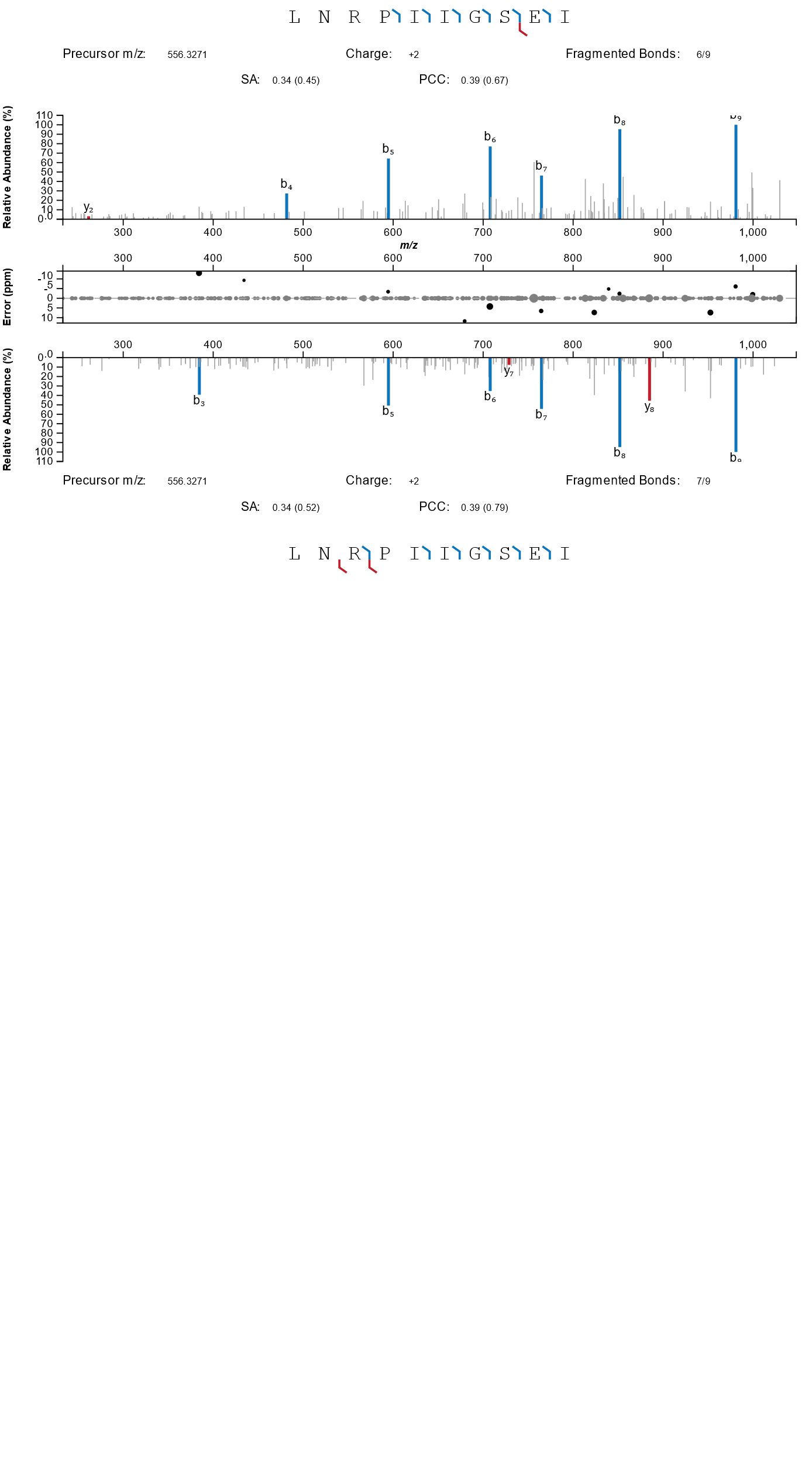

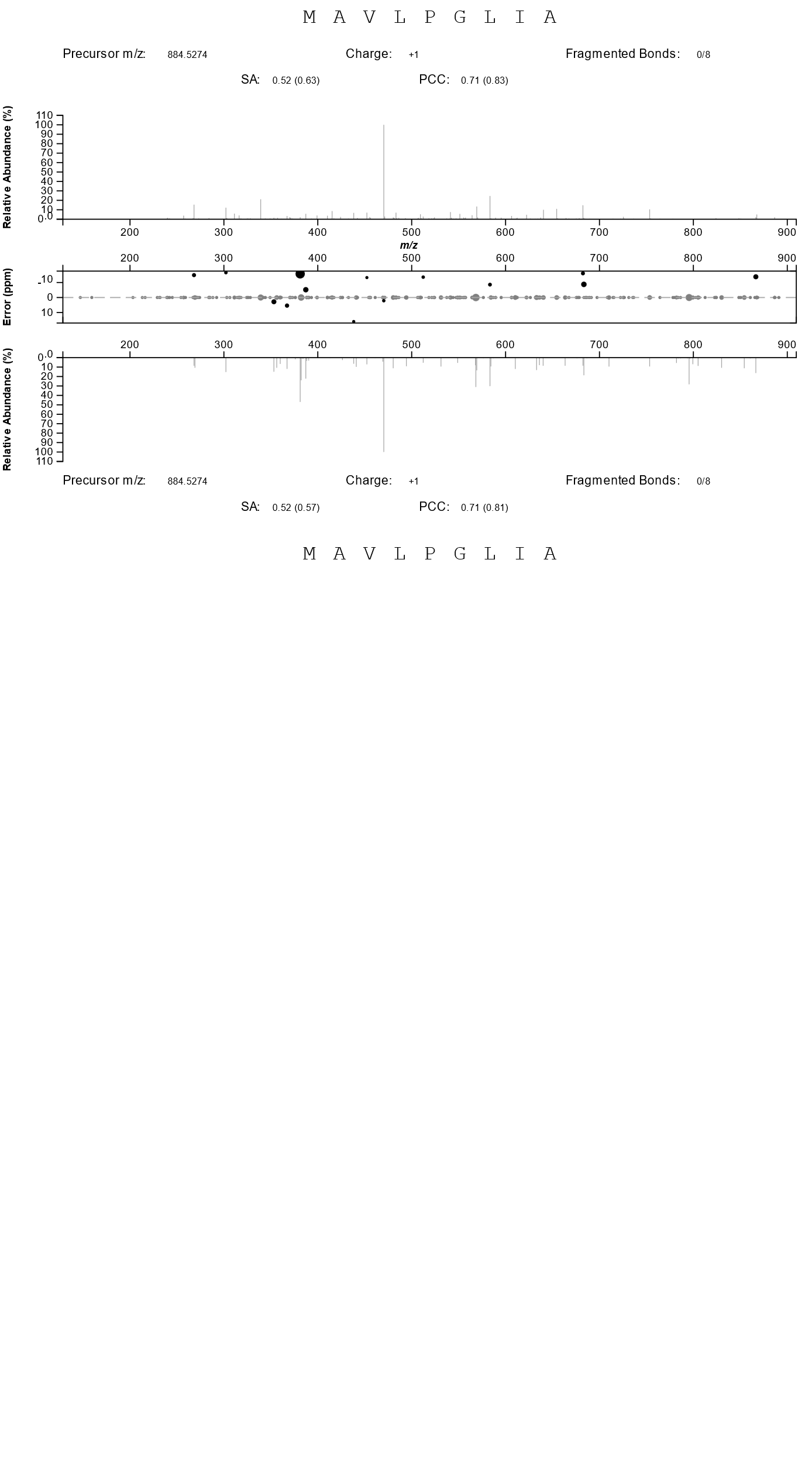

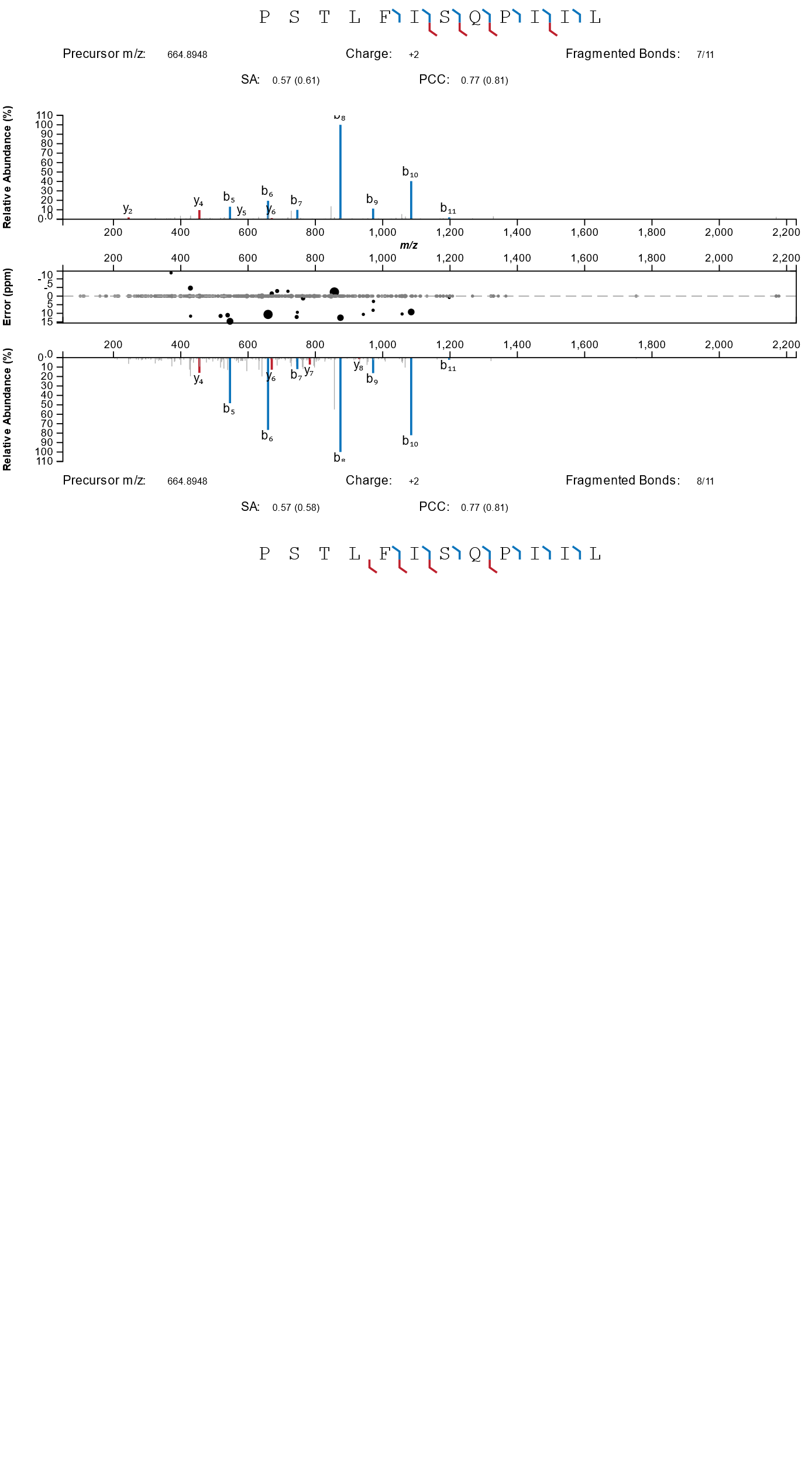

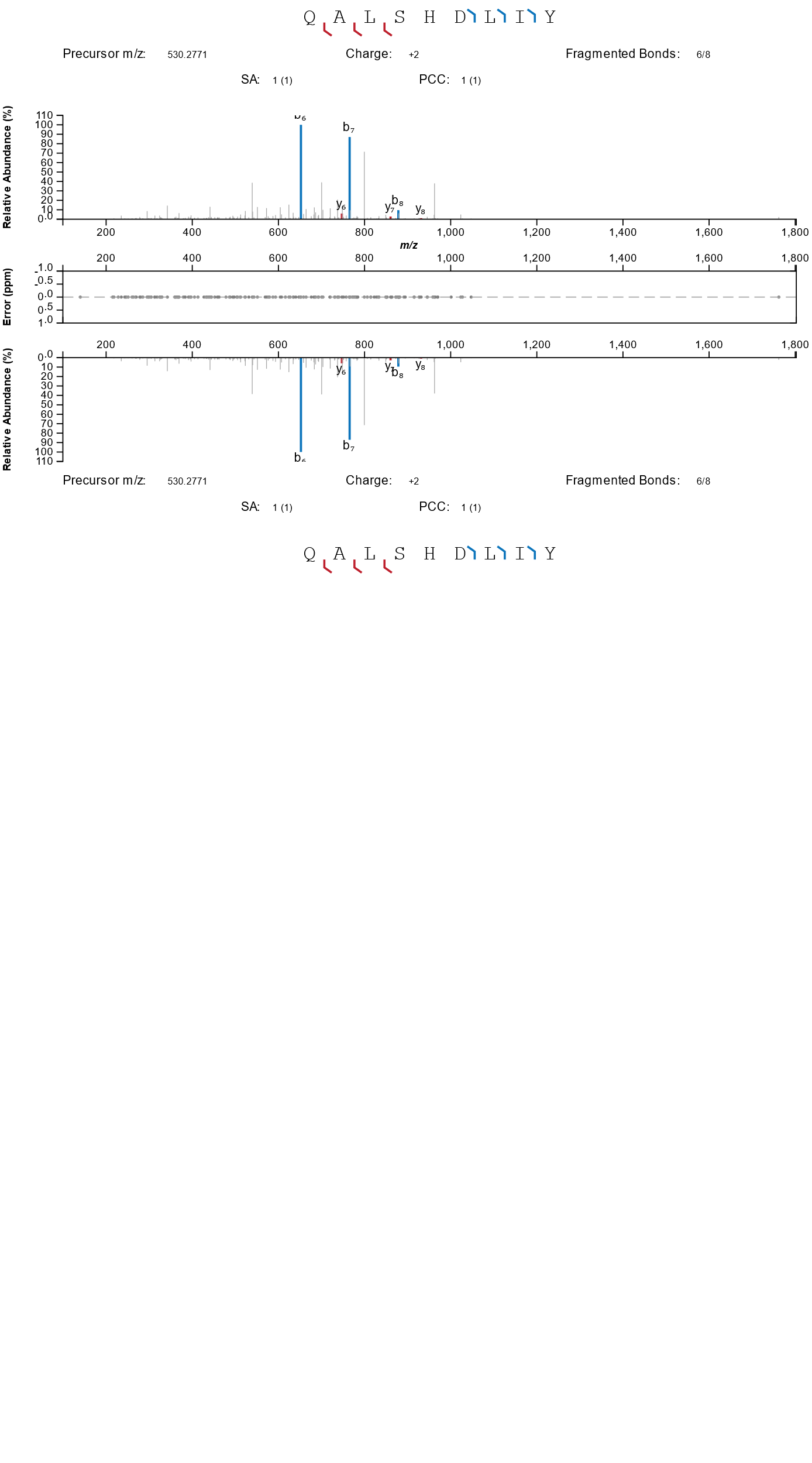

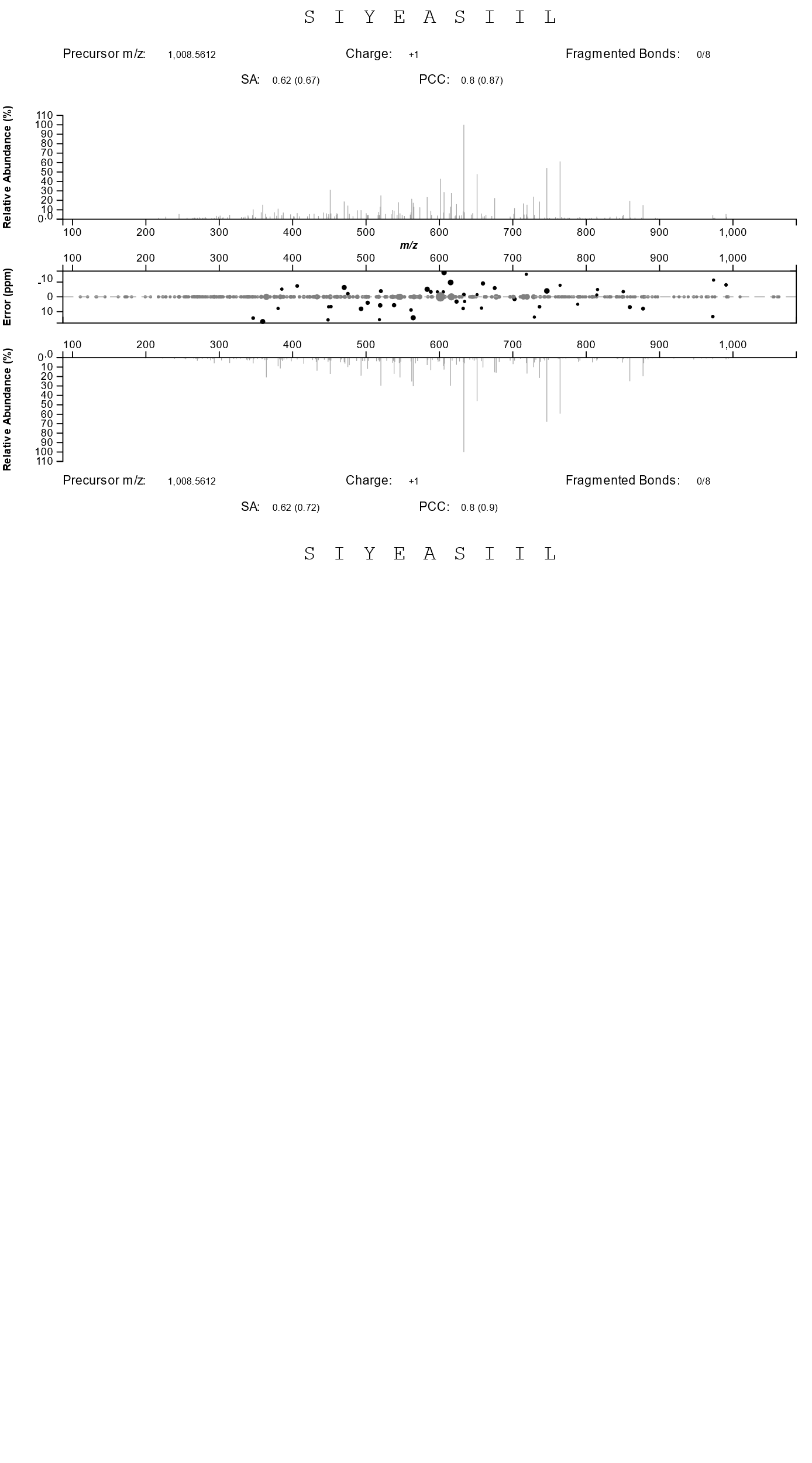

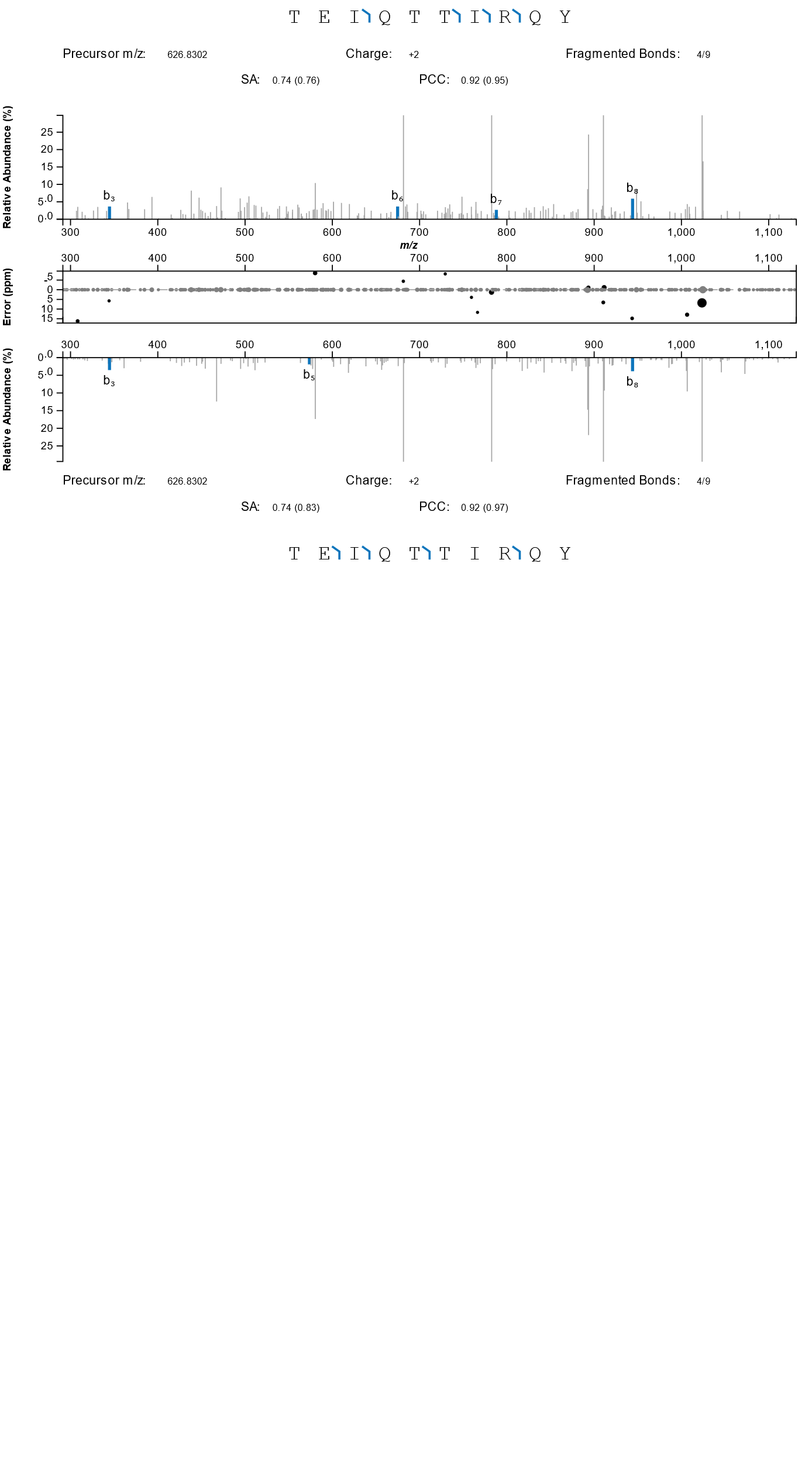

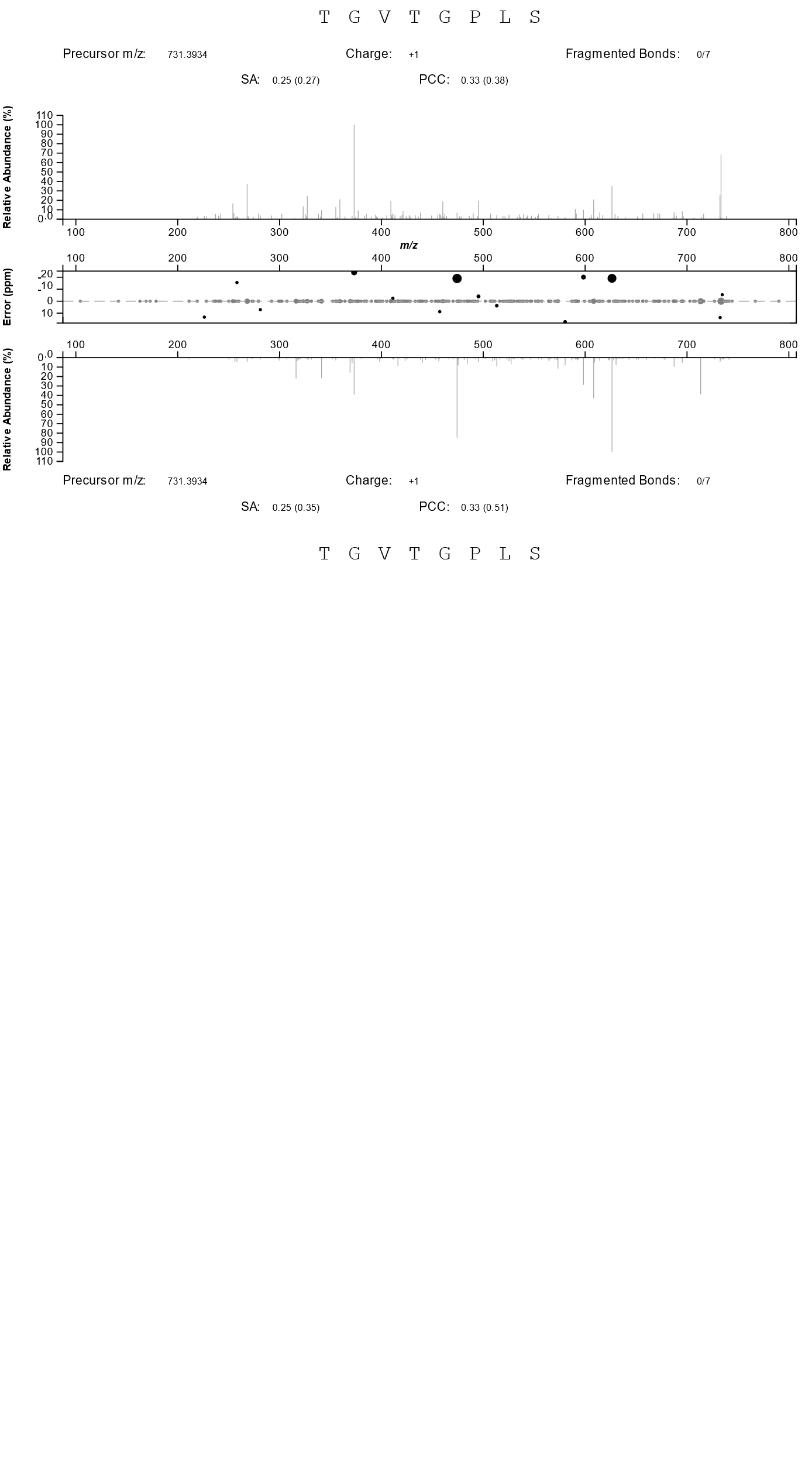

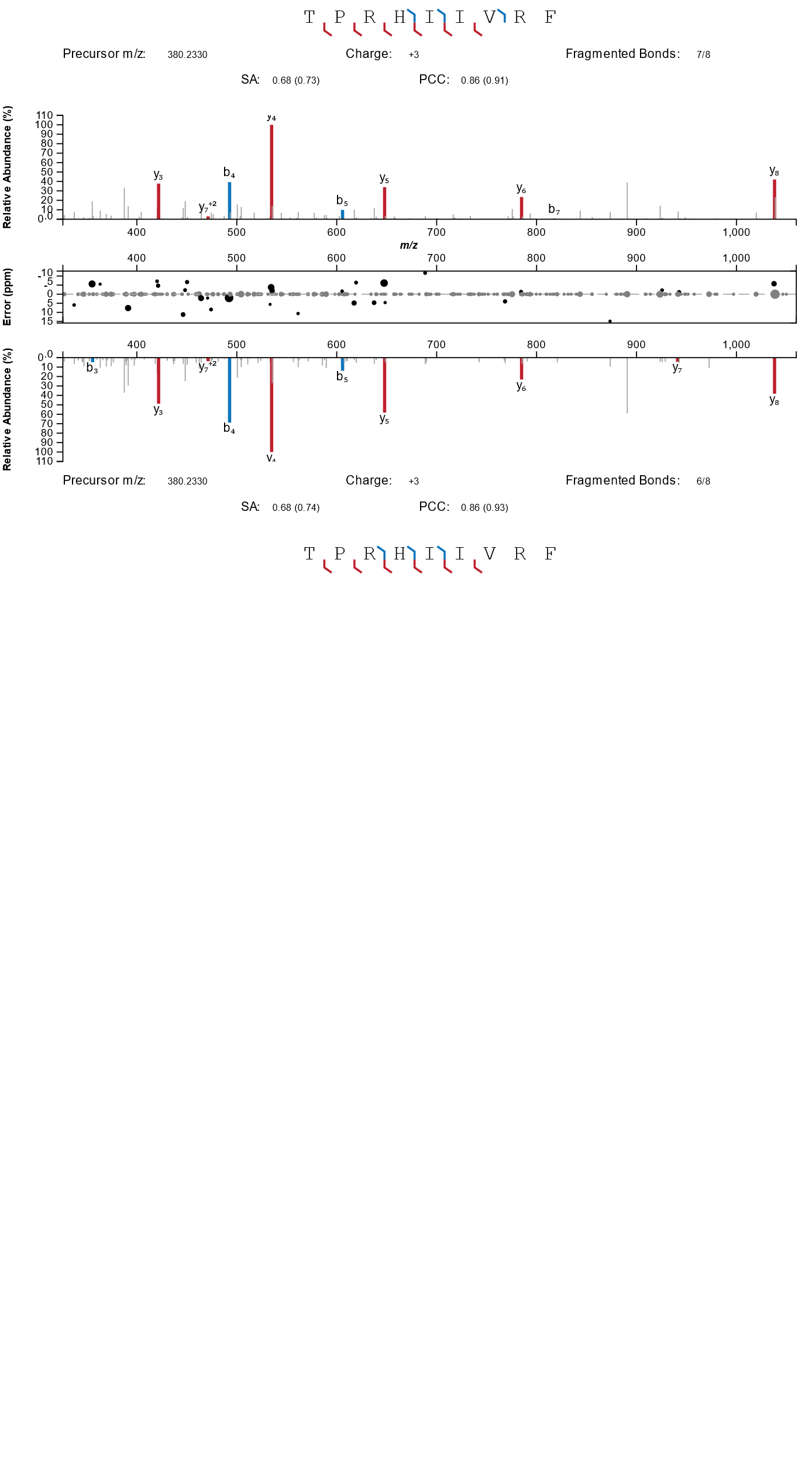

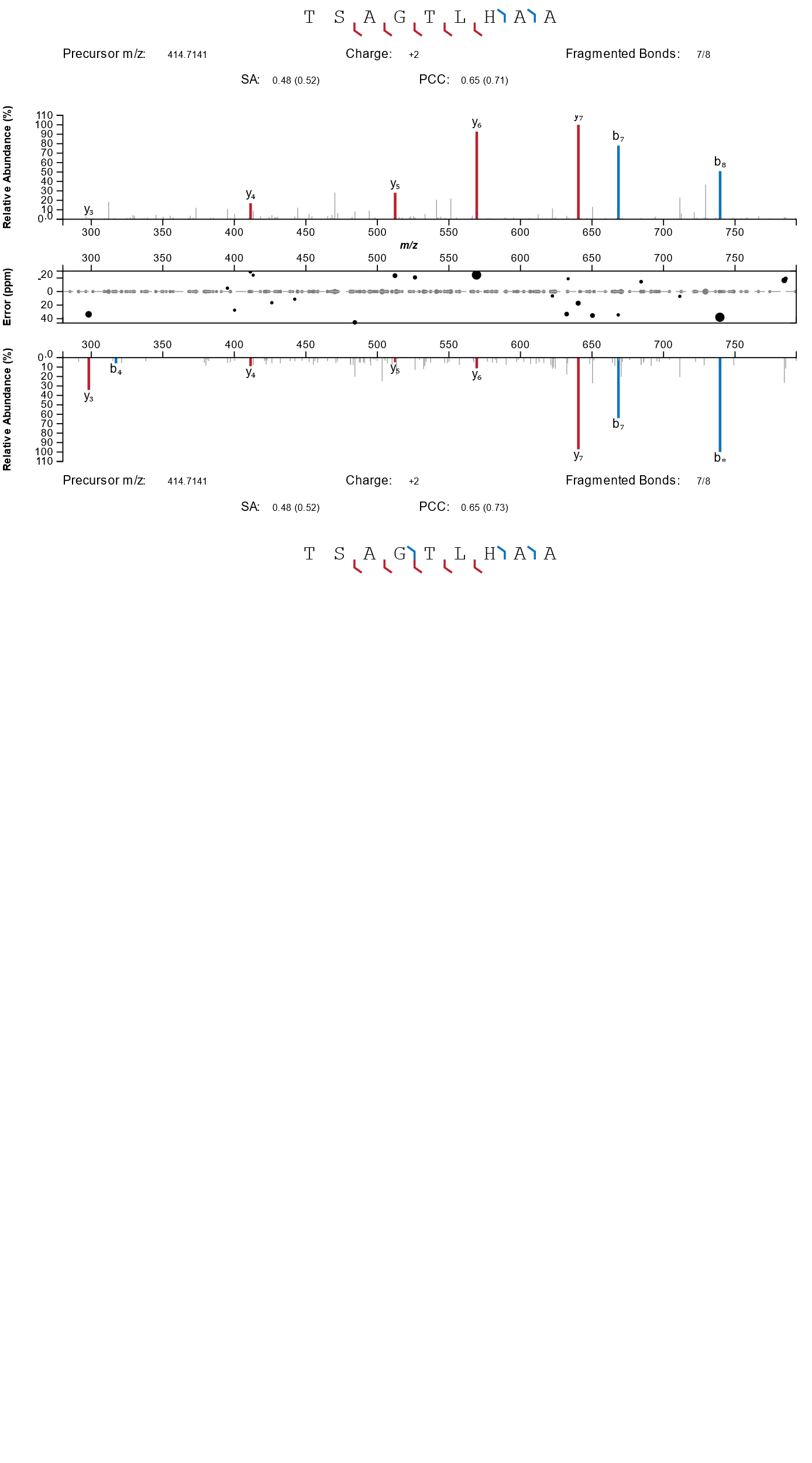

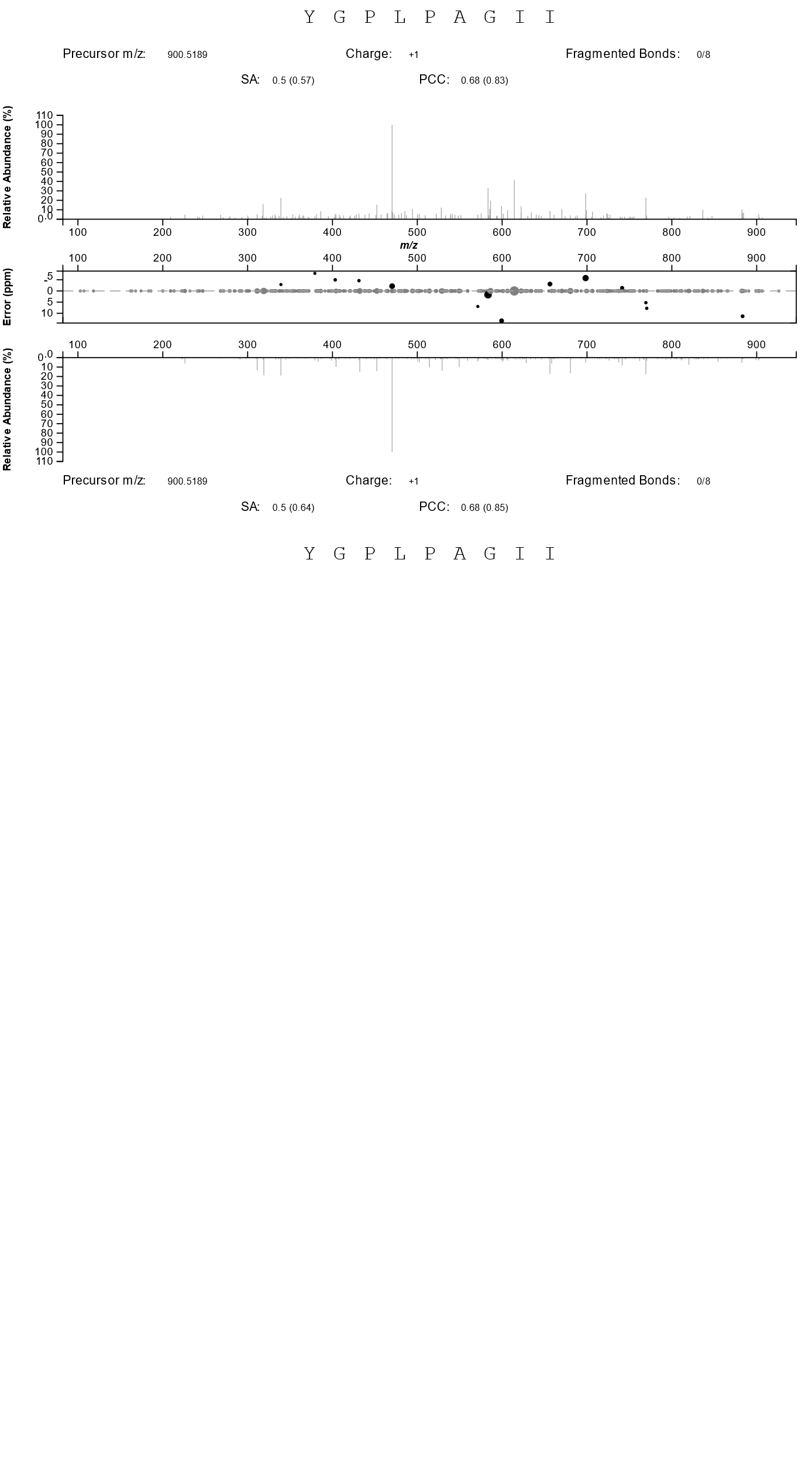


**Figure S6 – Mirror plots for HERV peptides identified in cell line**


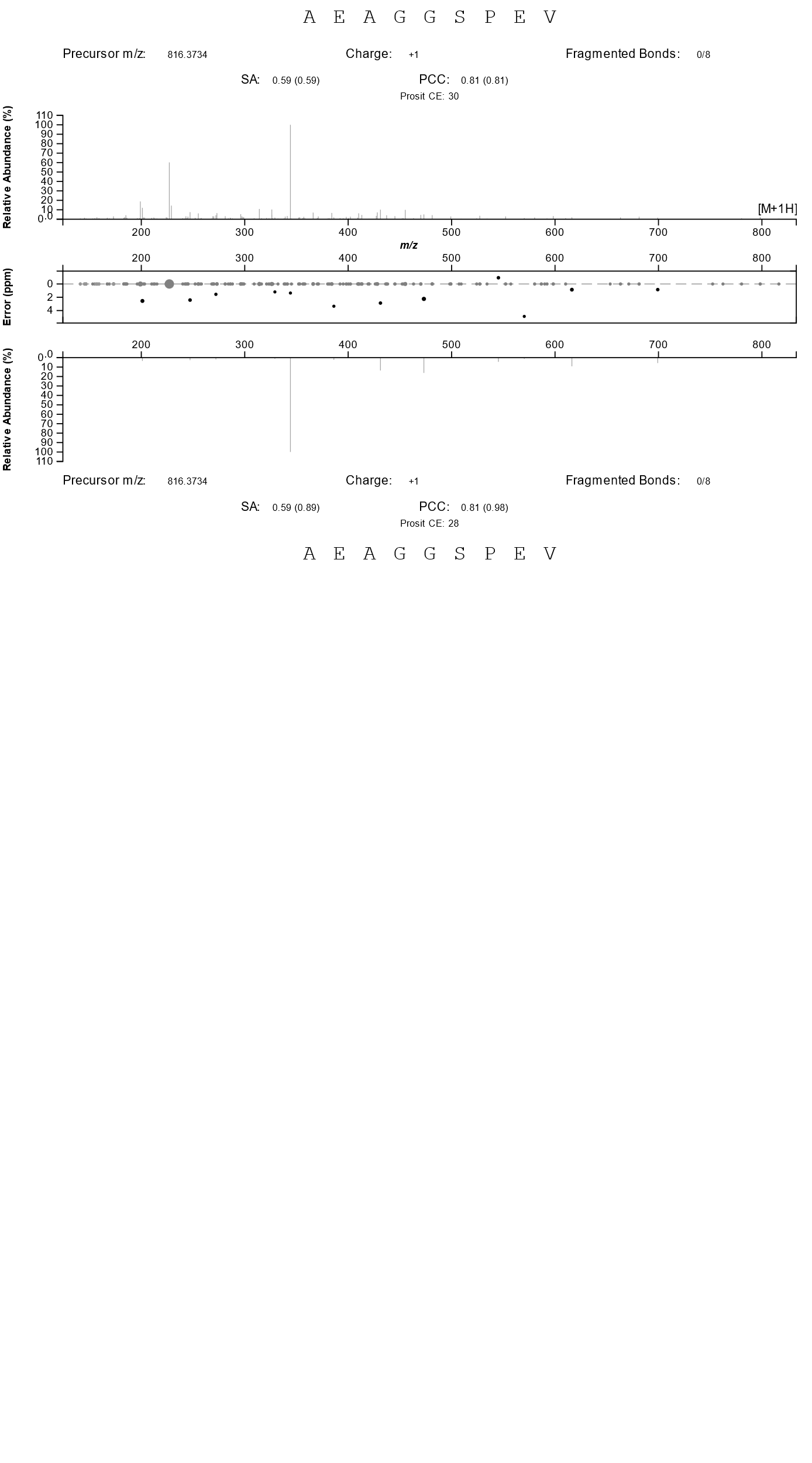

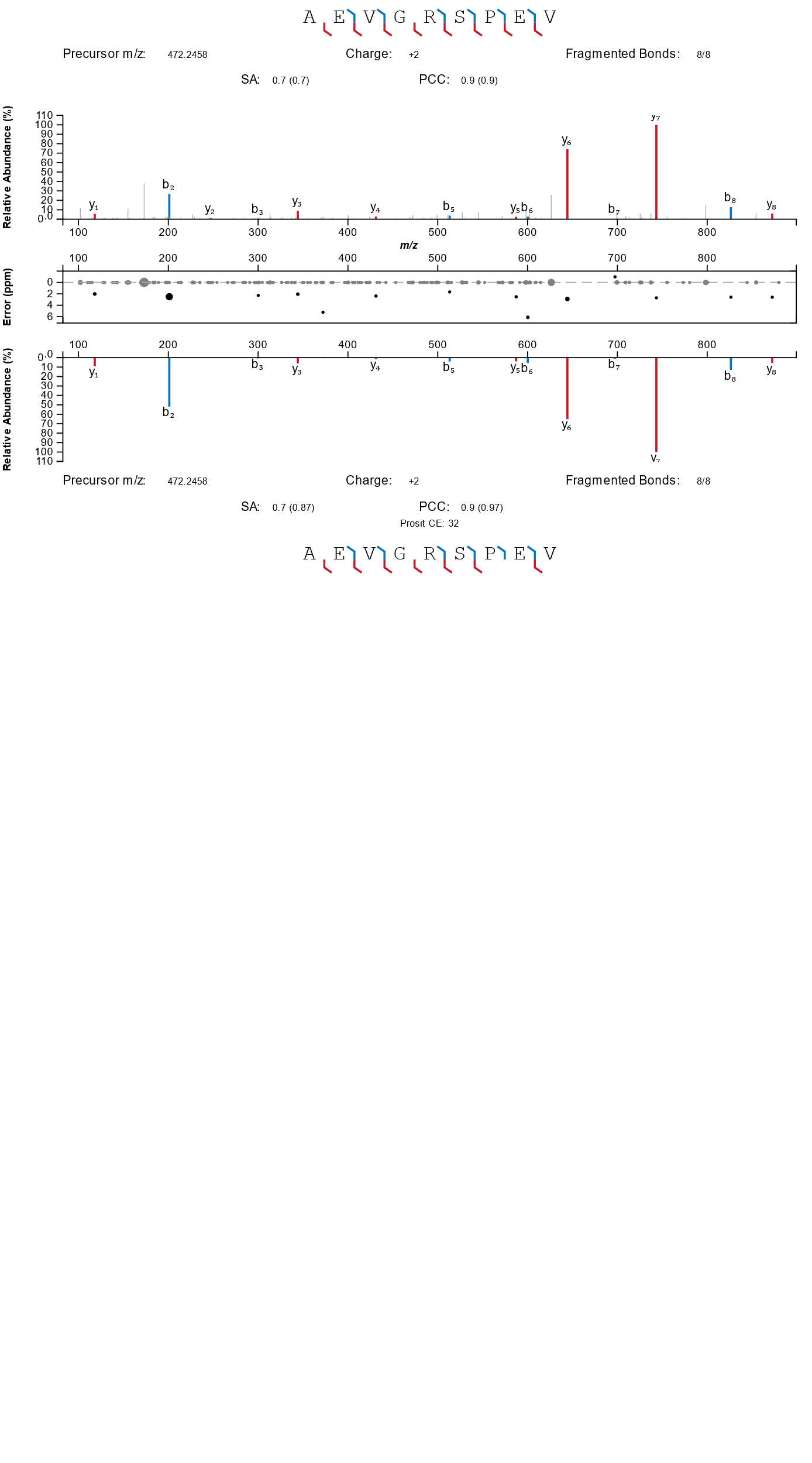

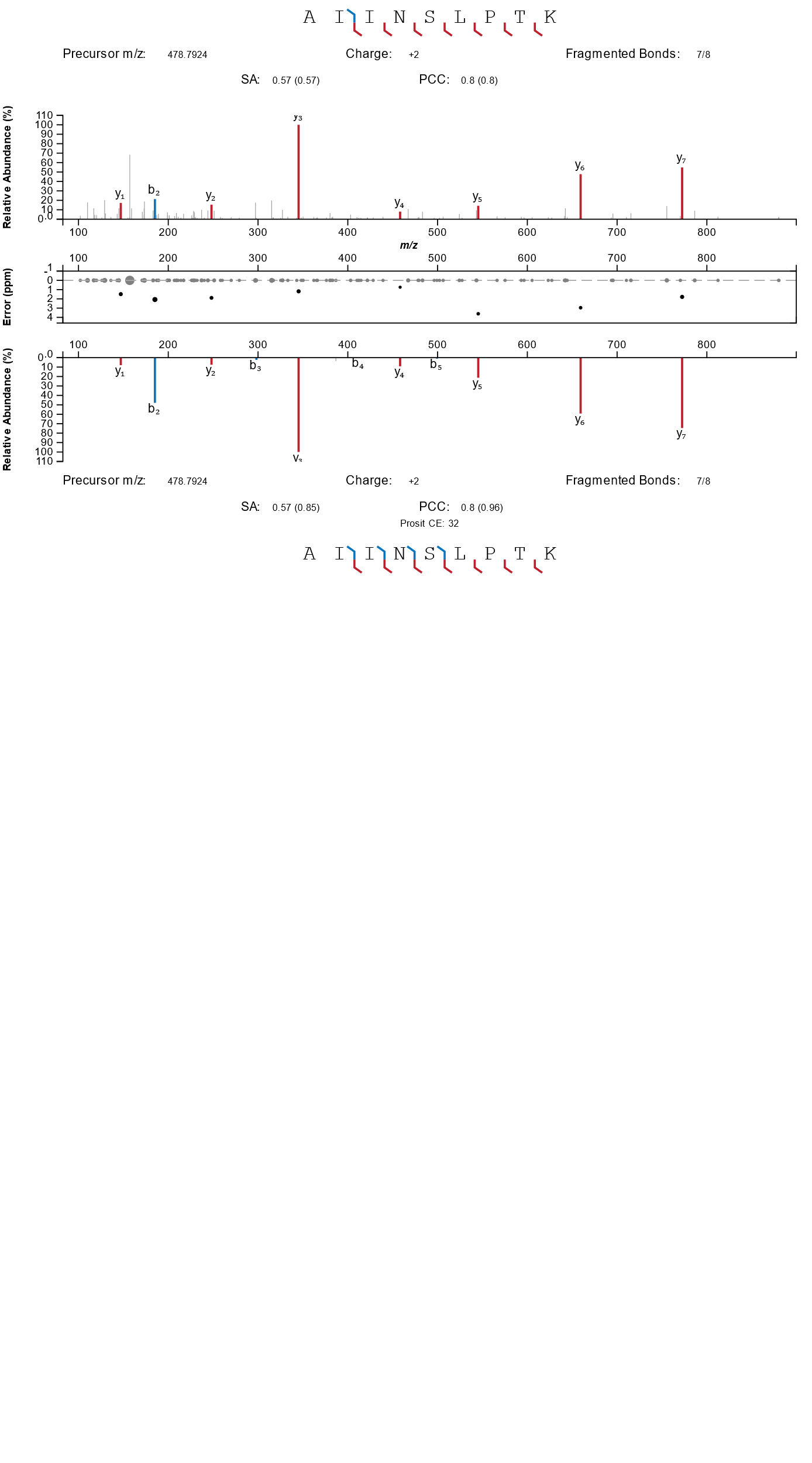

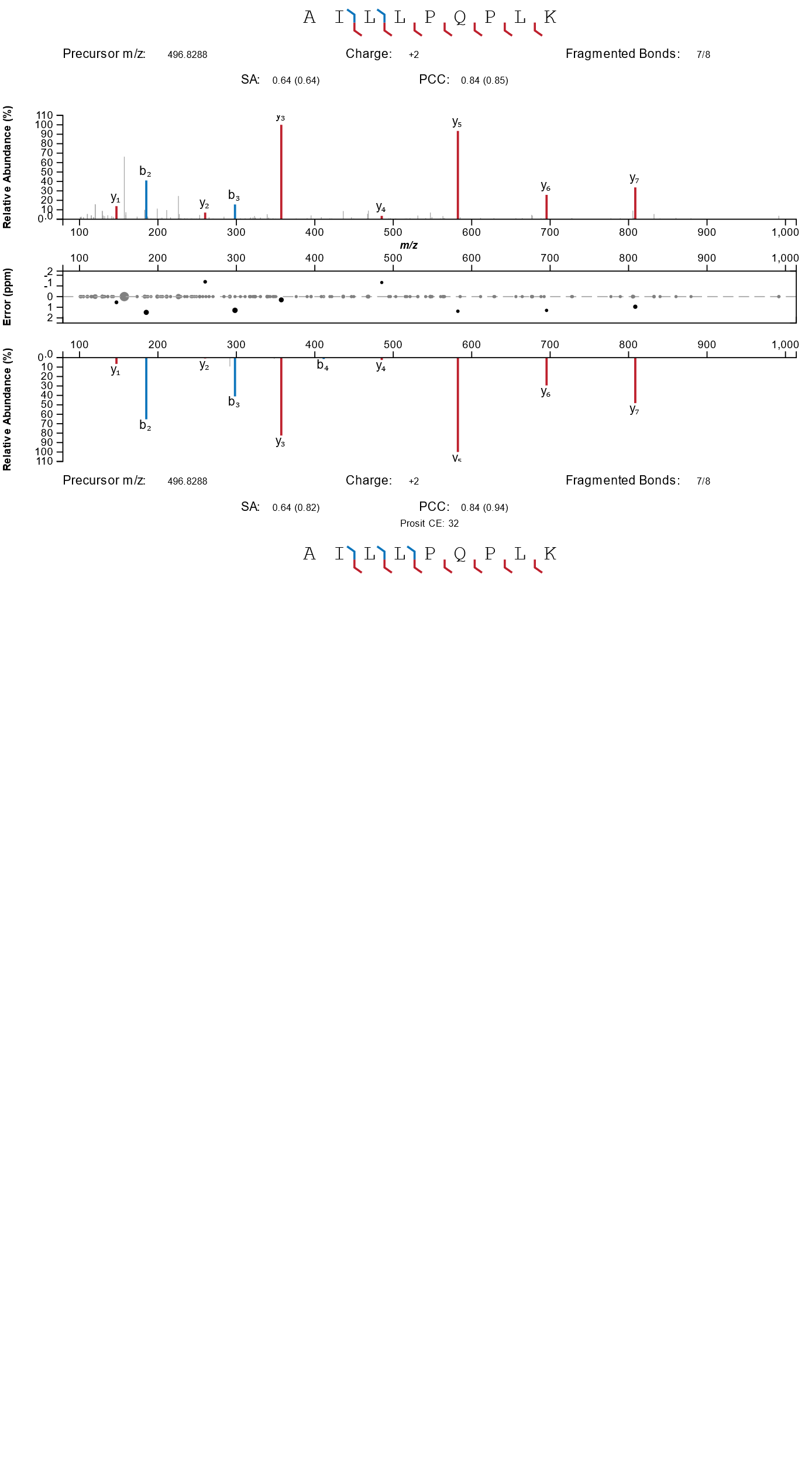

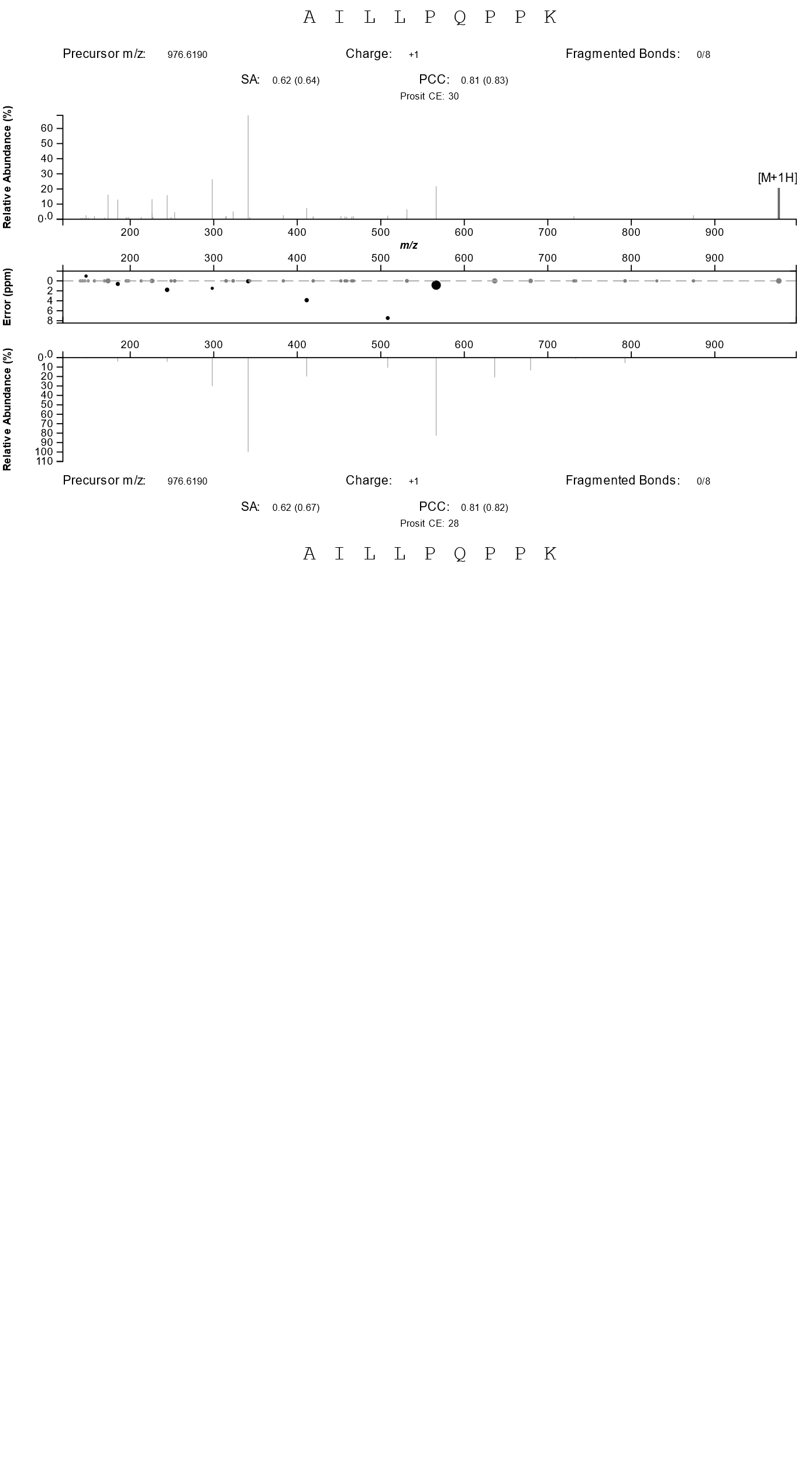

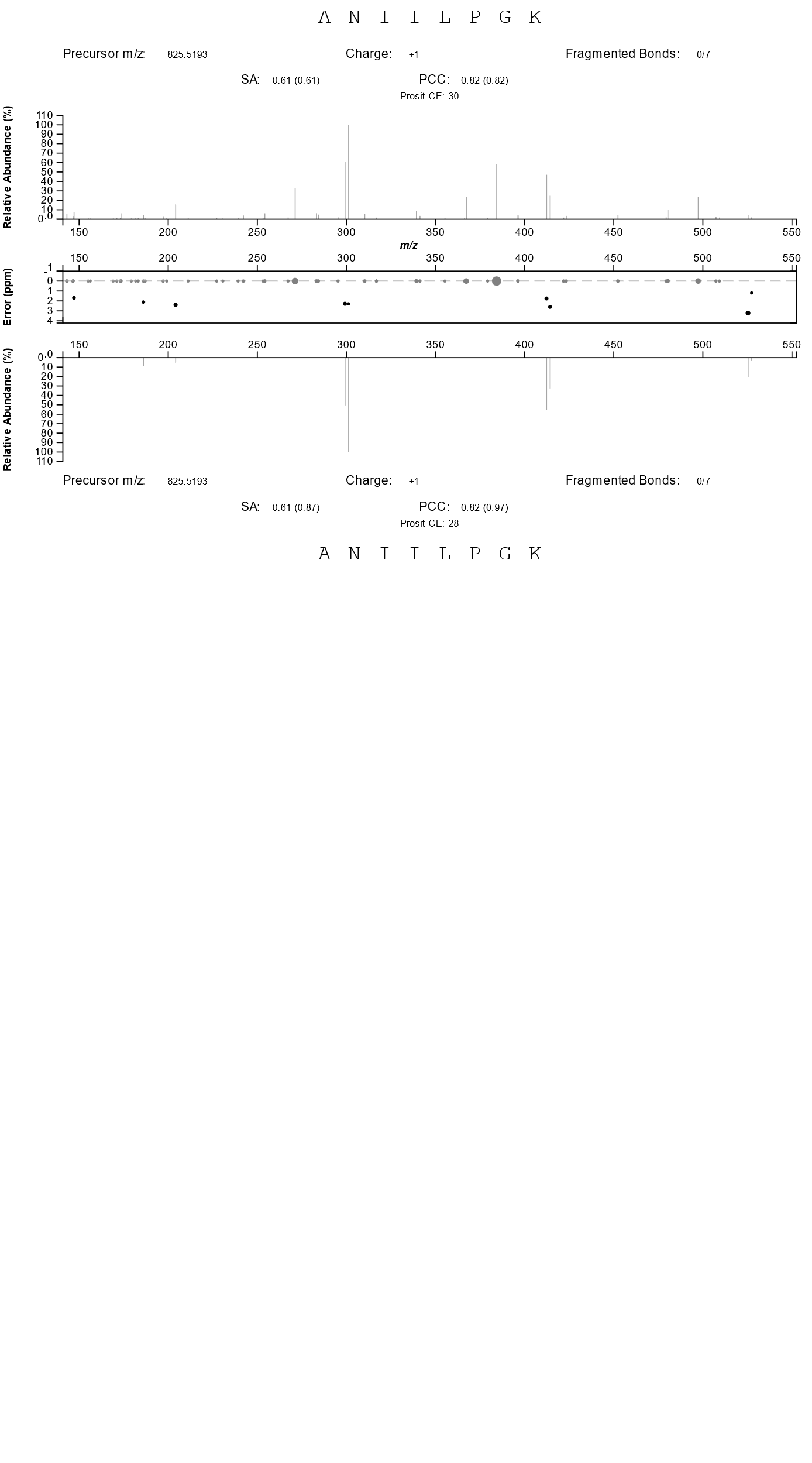

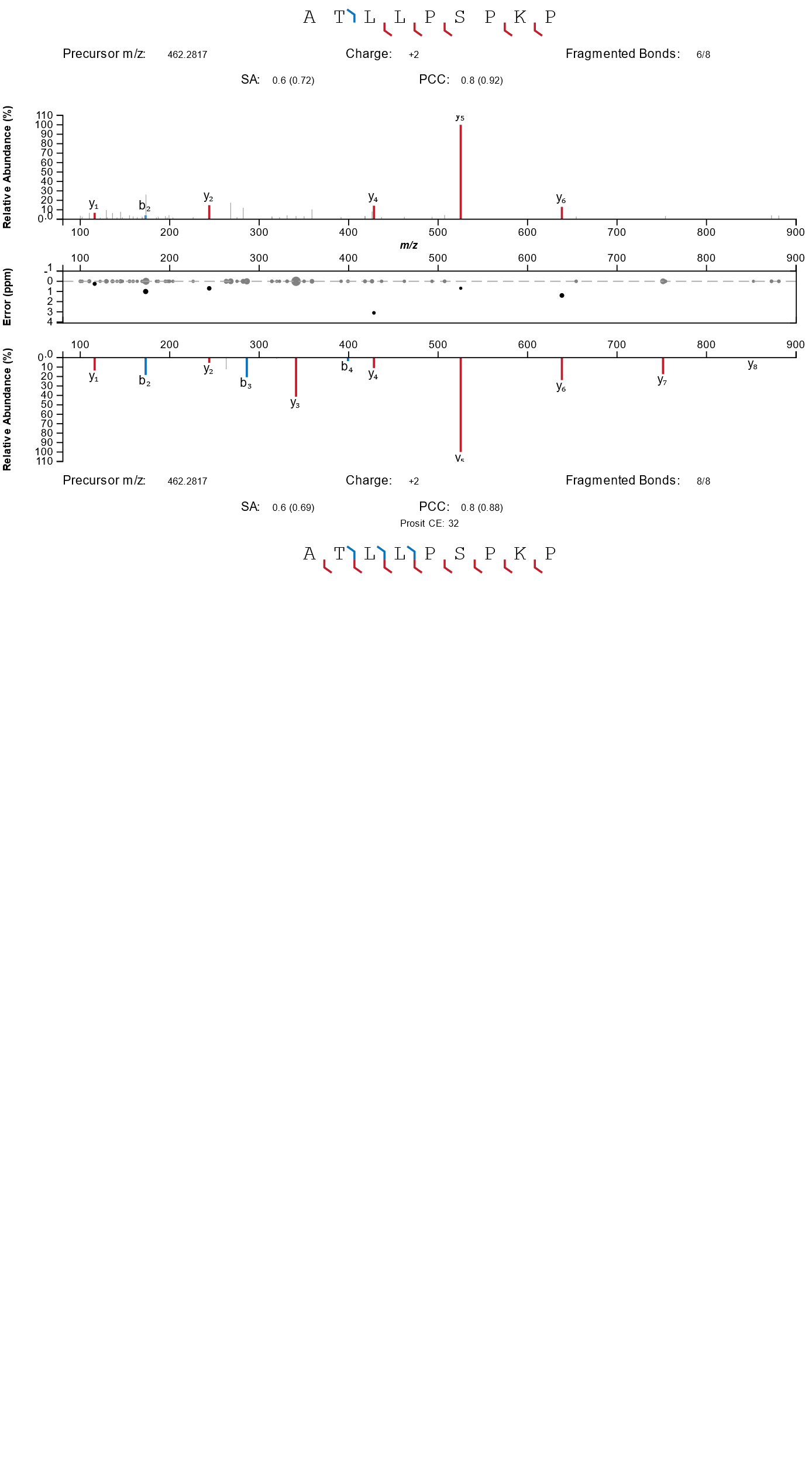

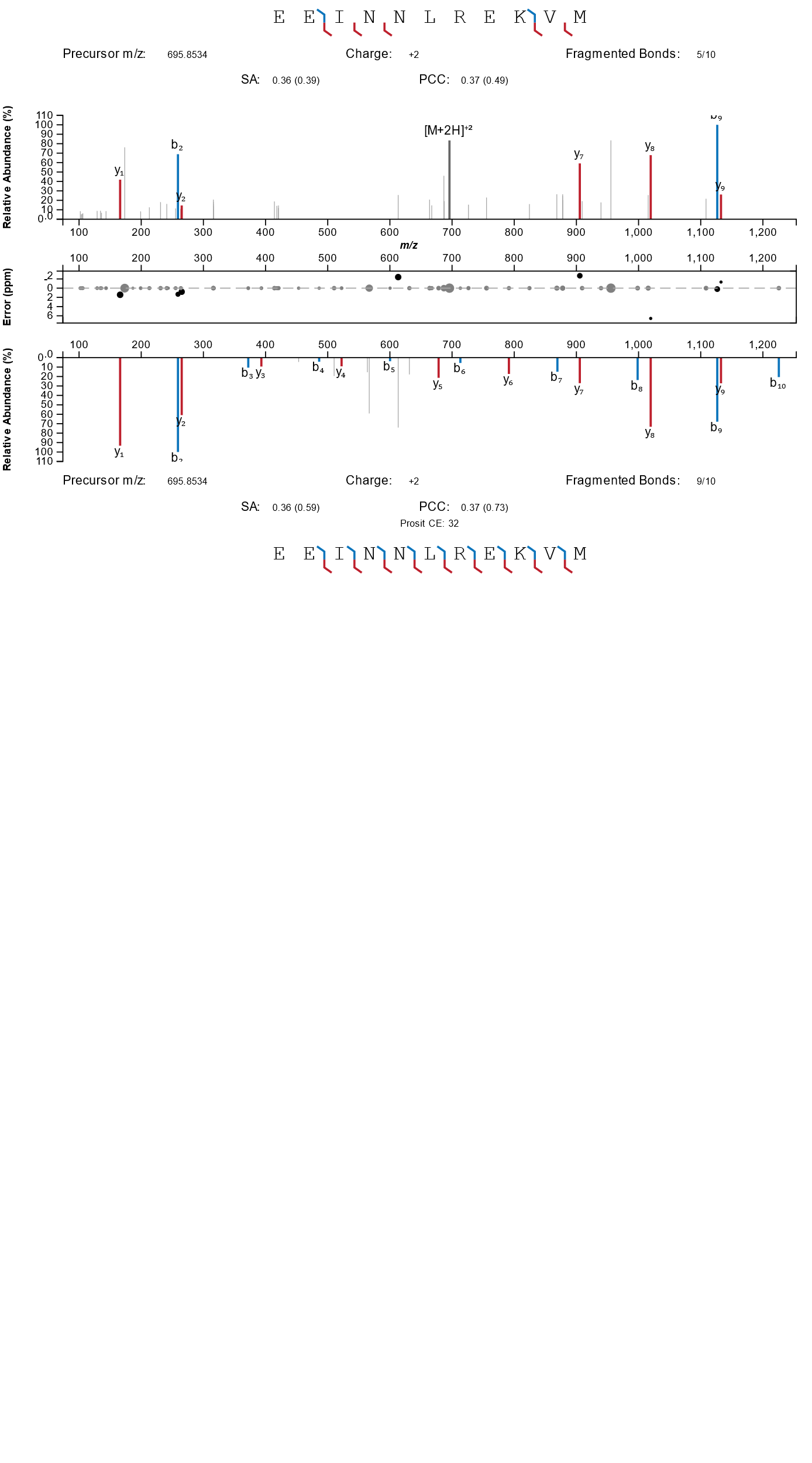

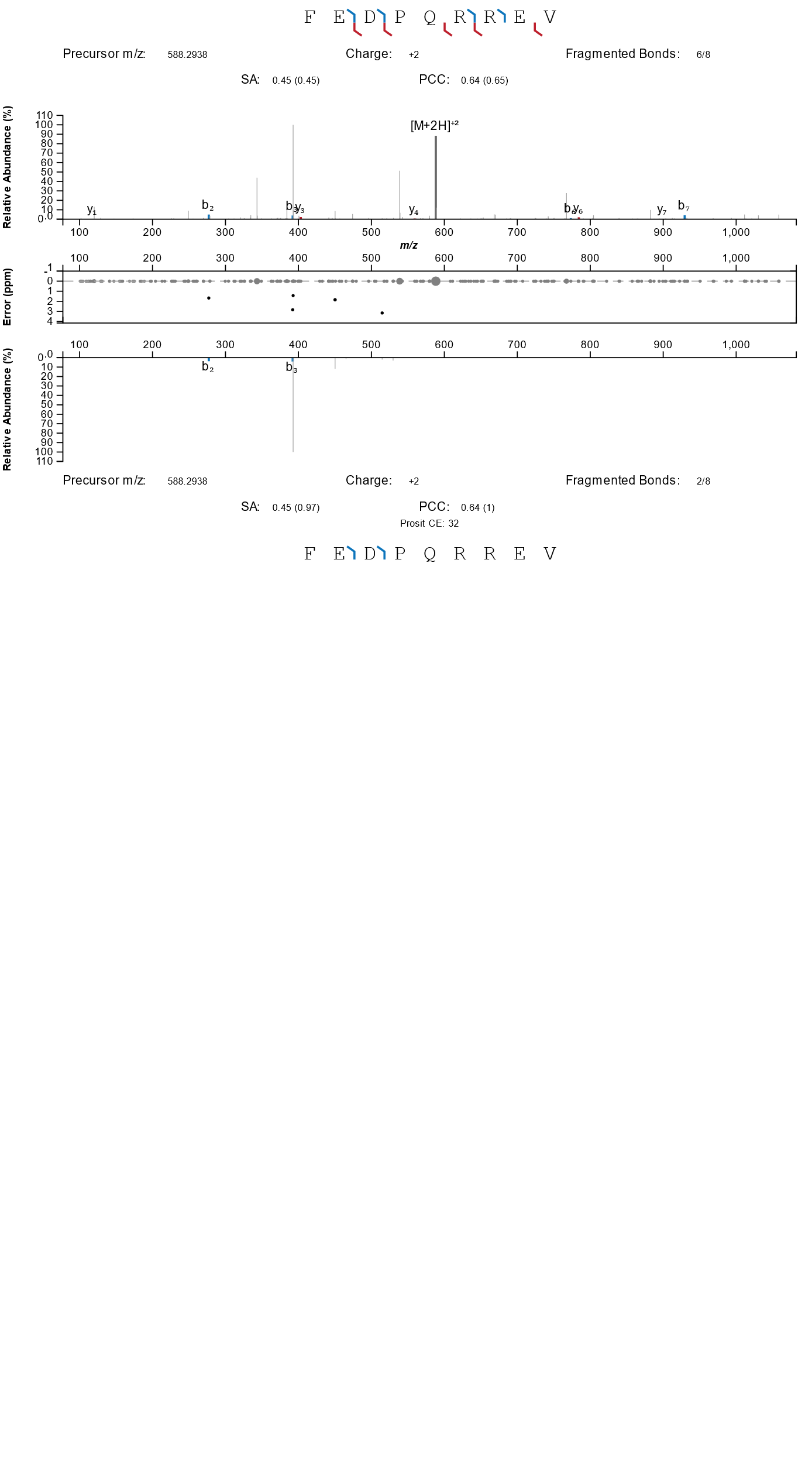

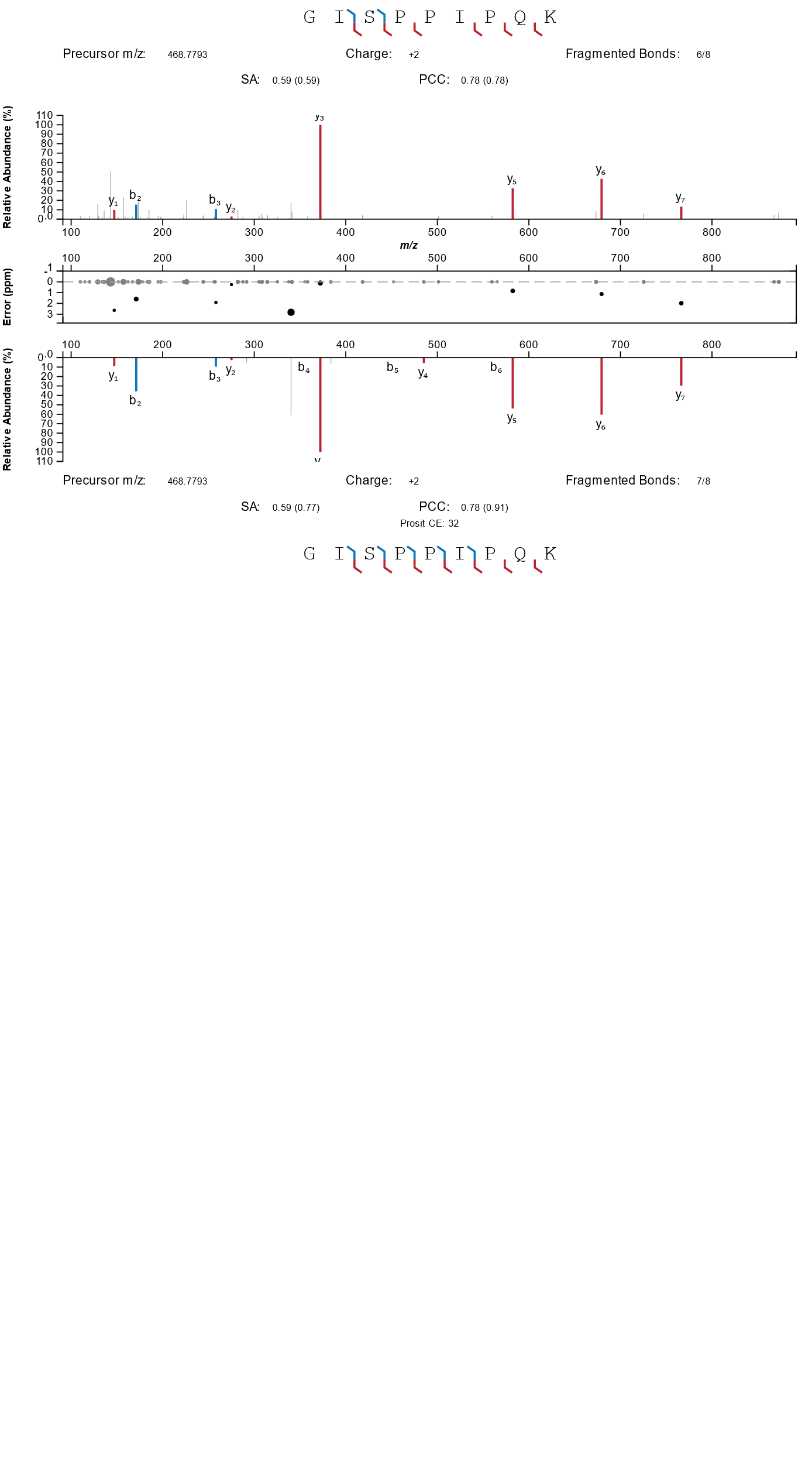

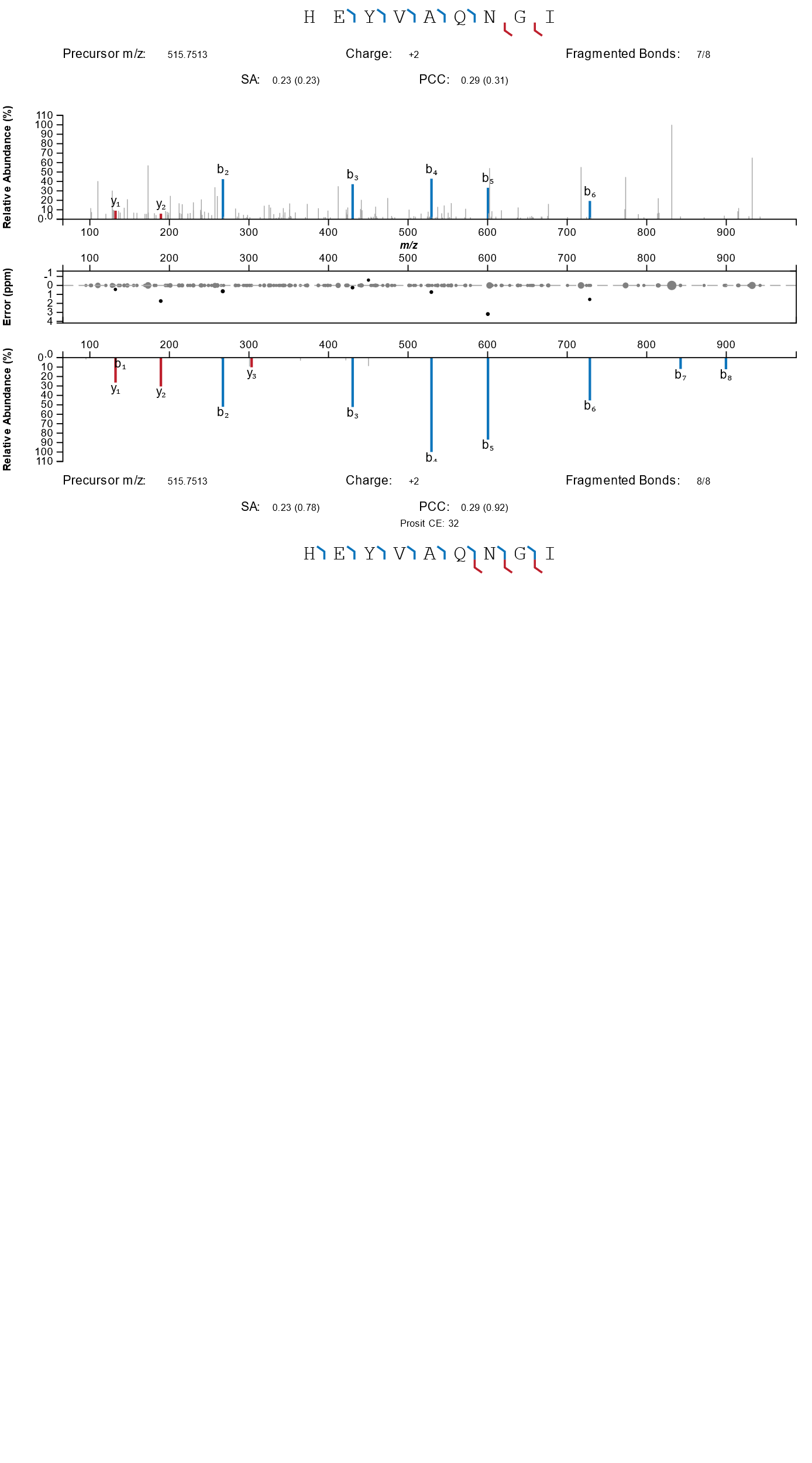

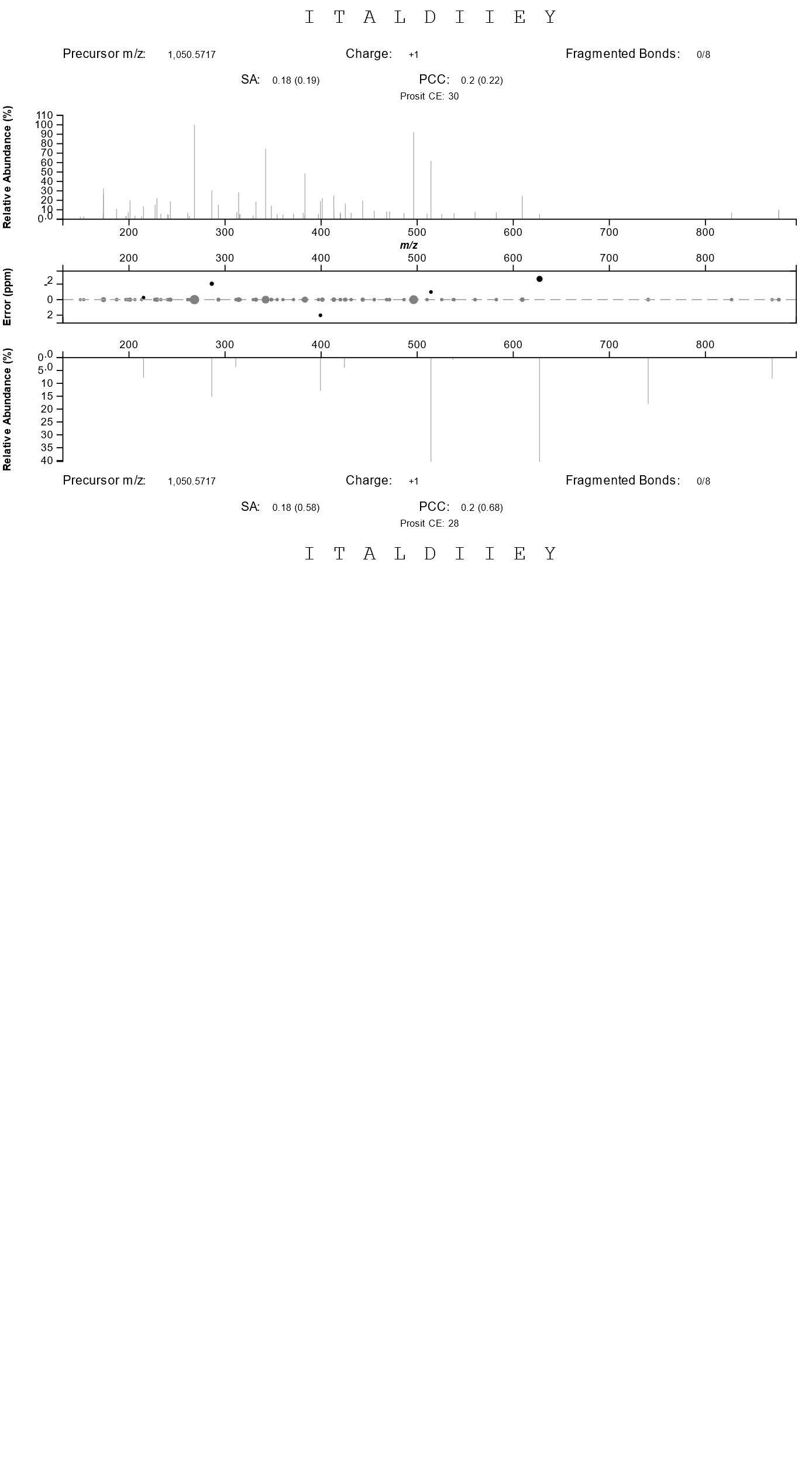

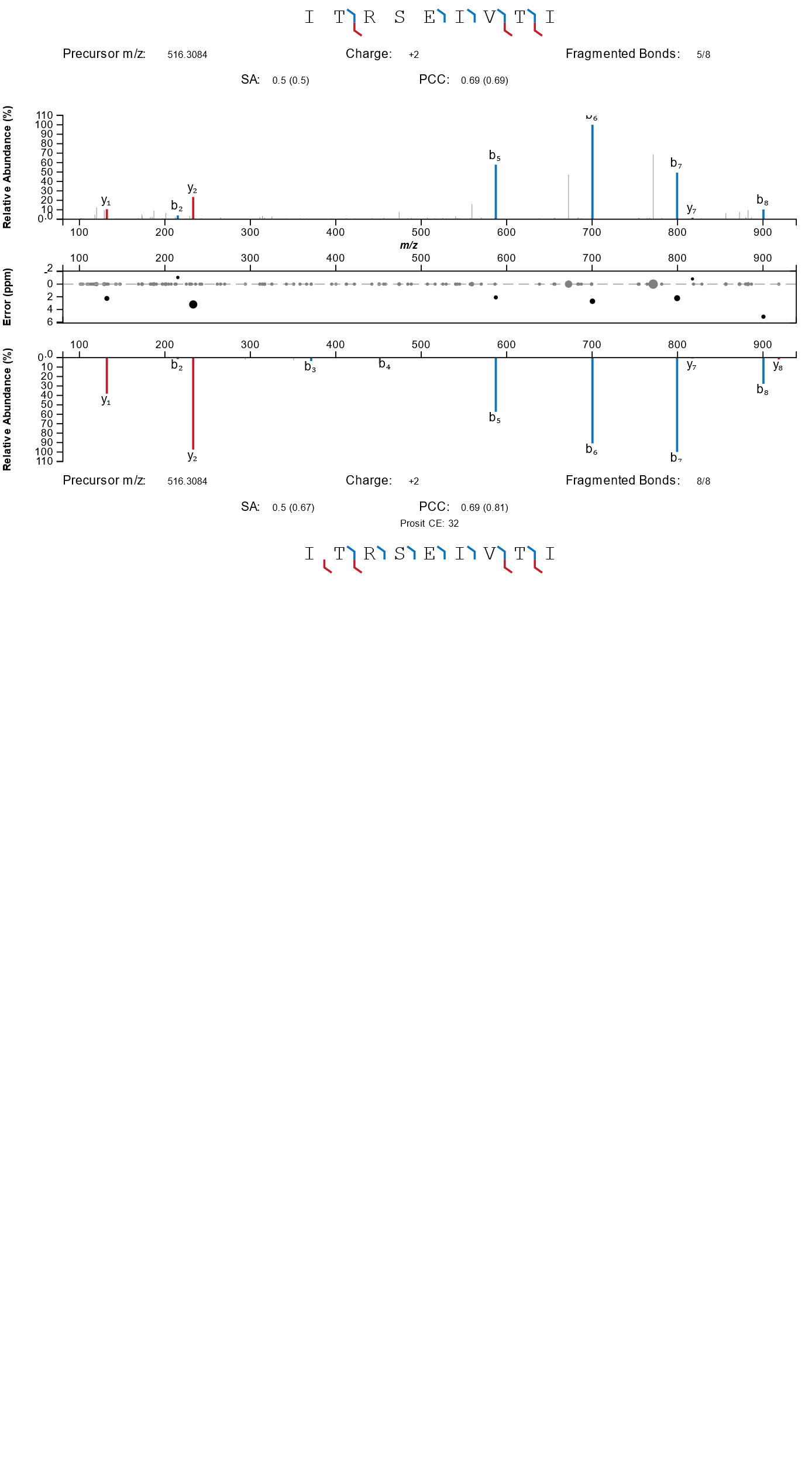

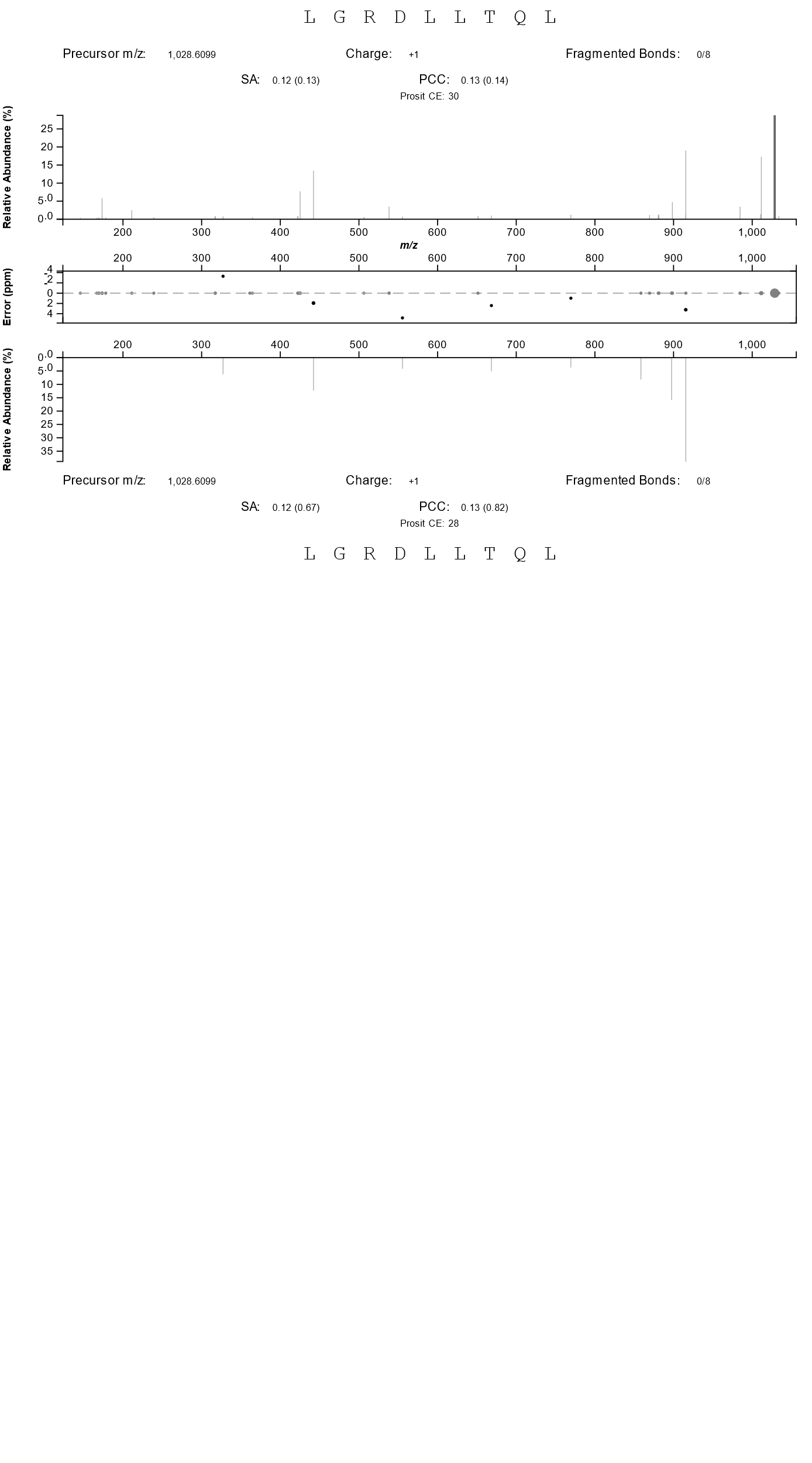

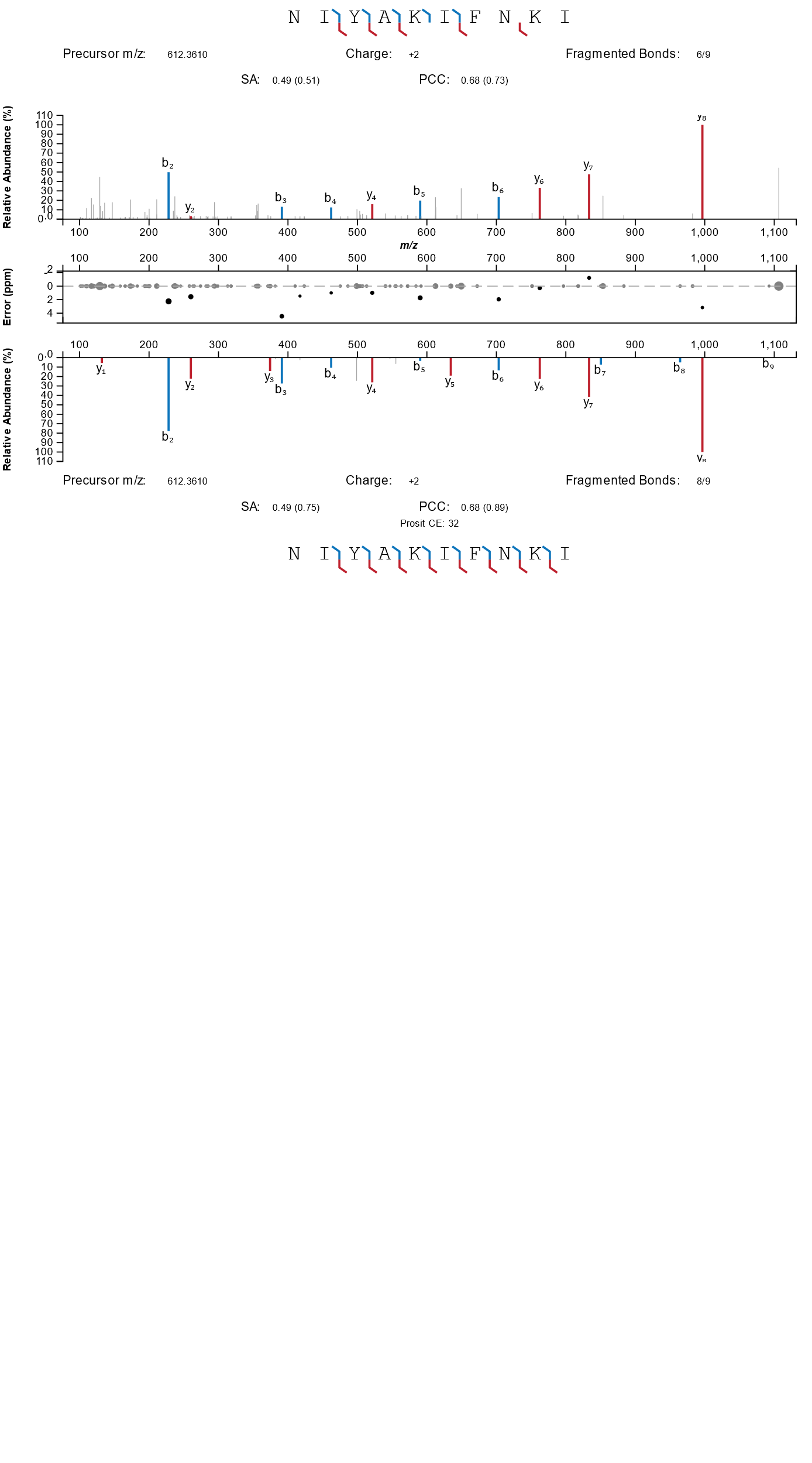

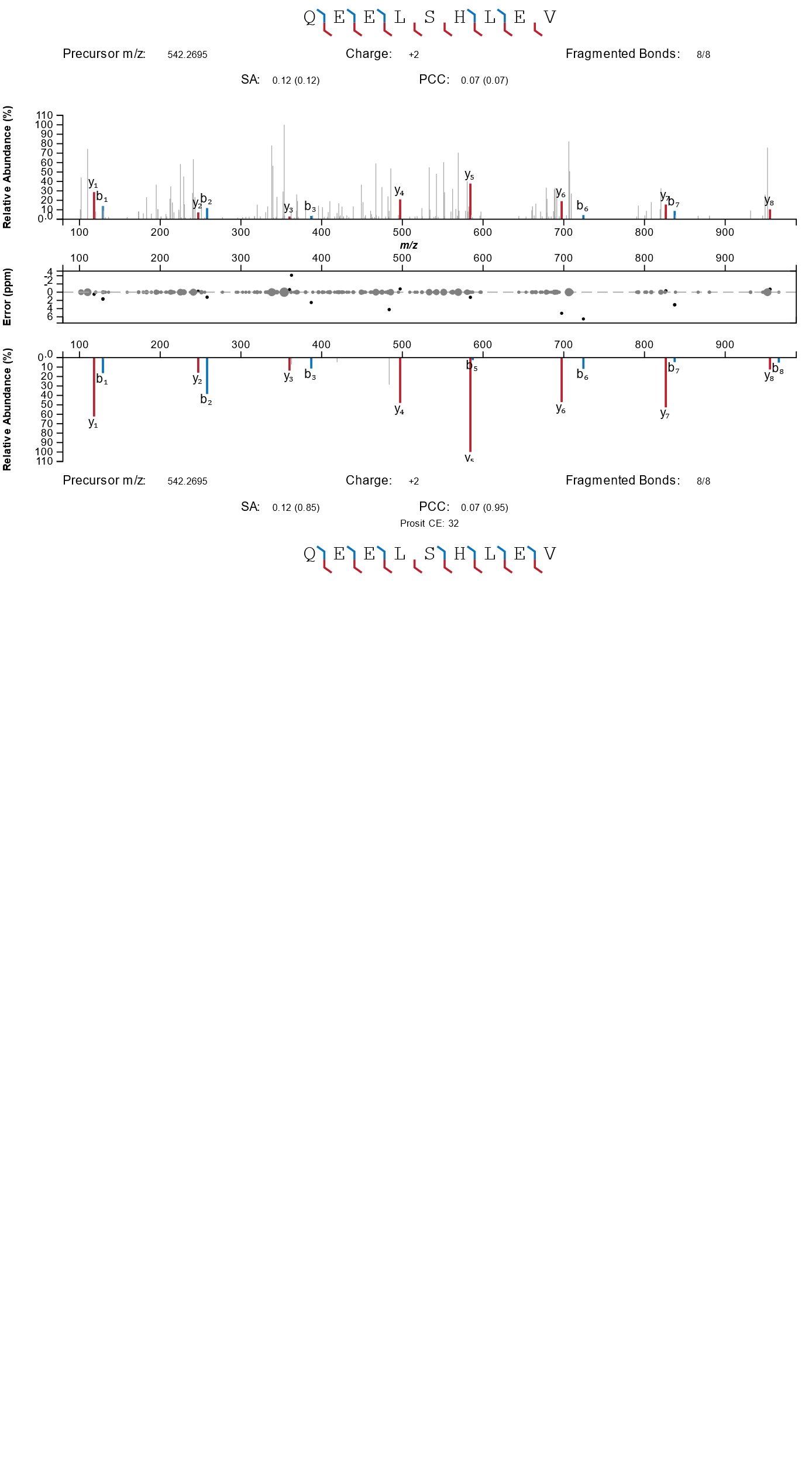

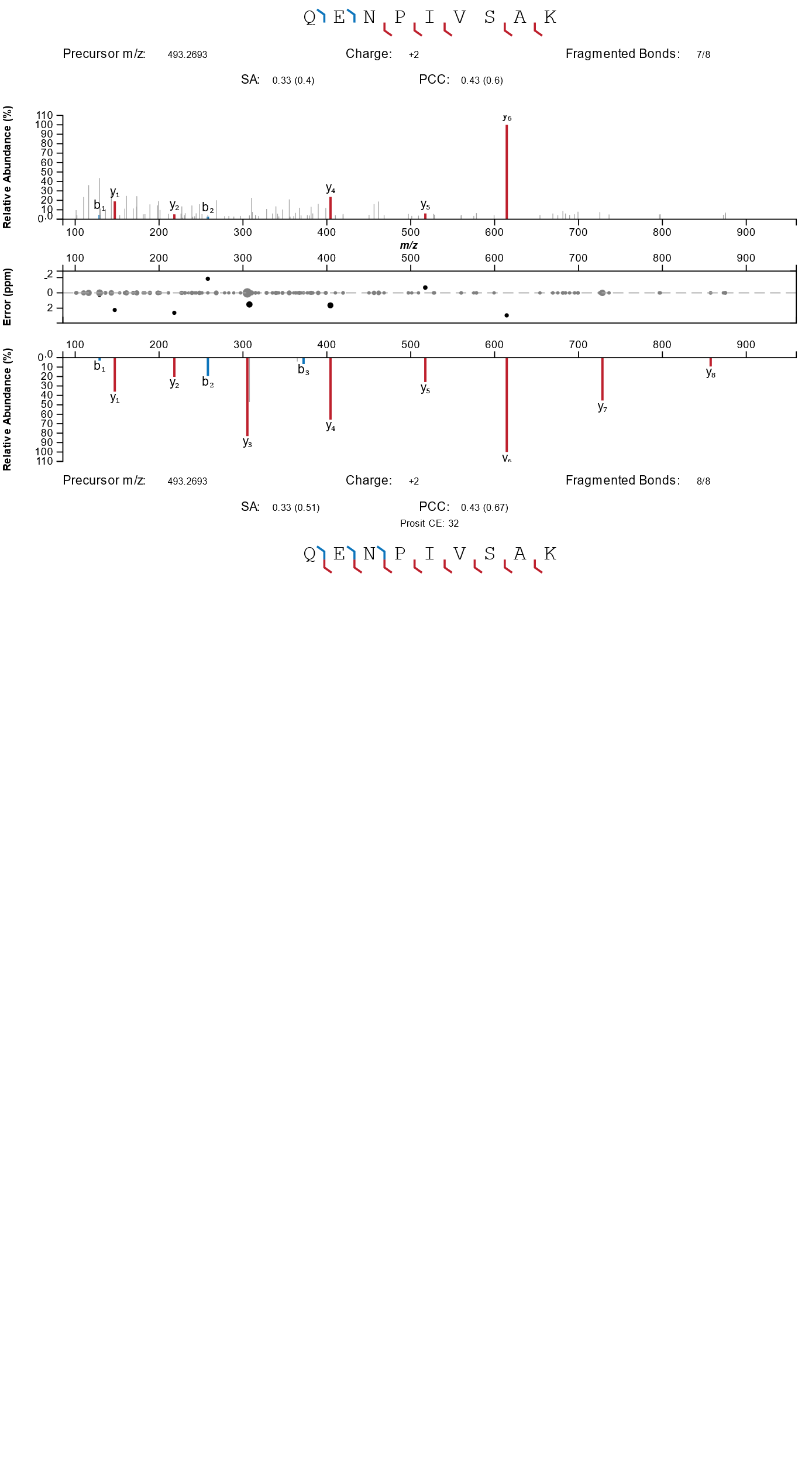

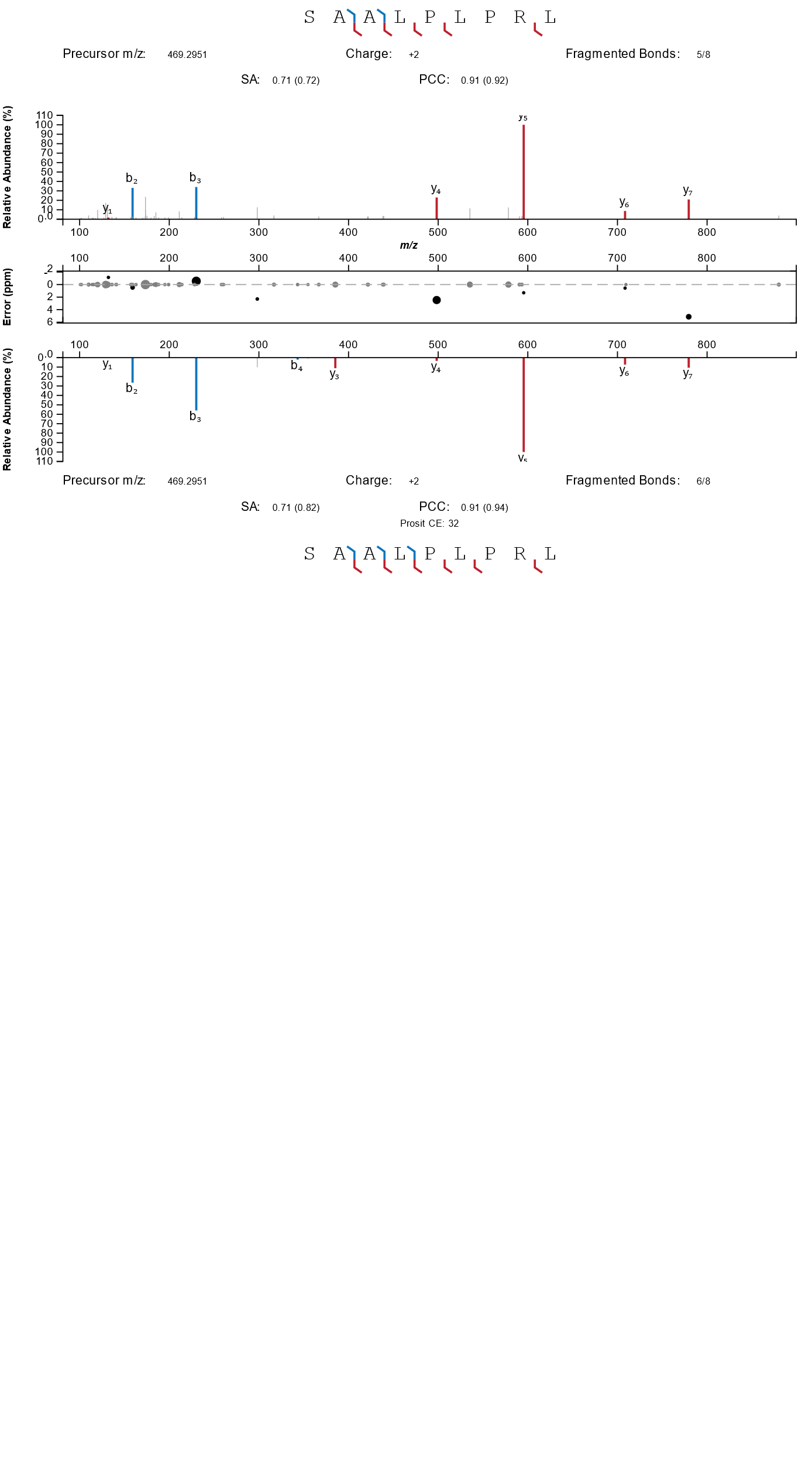

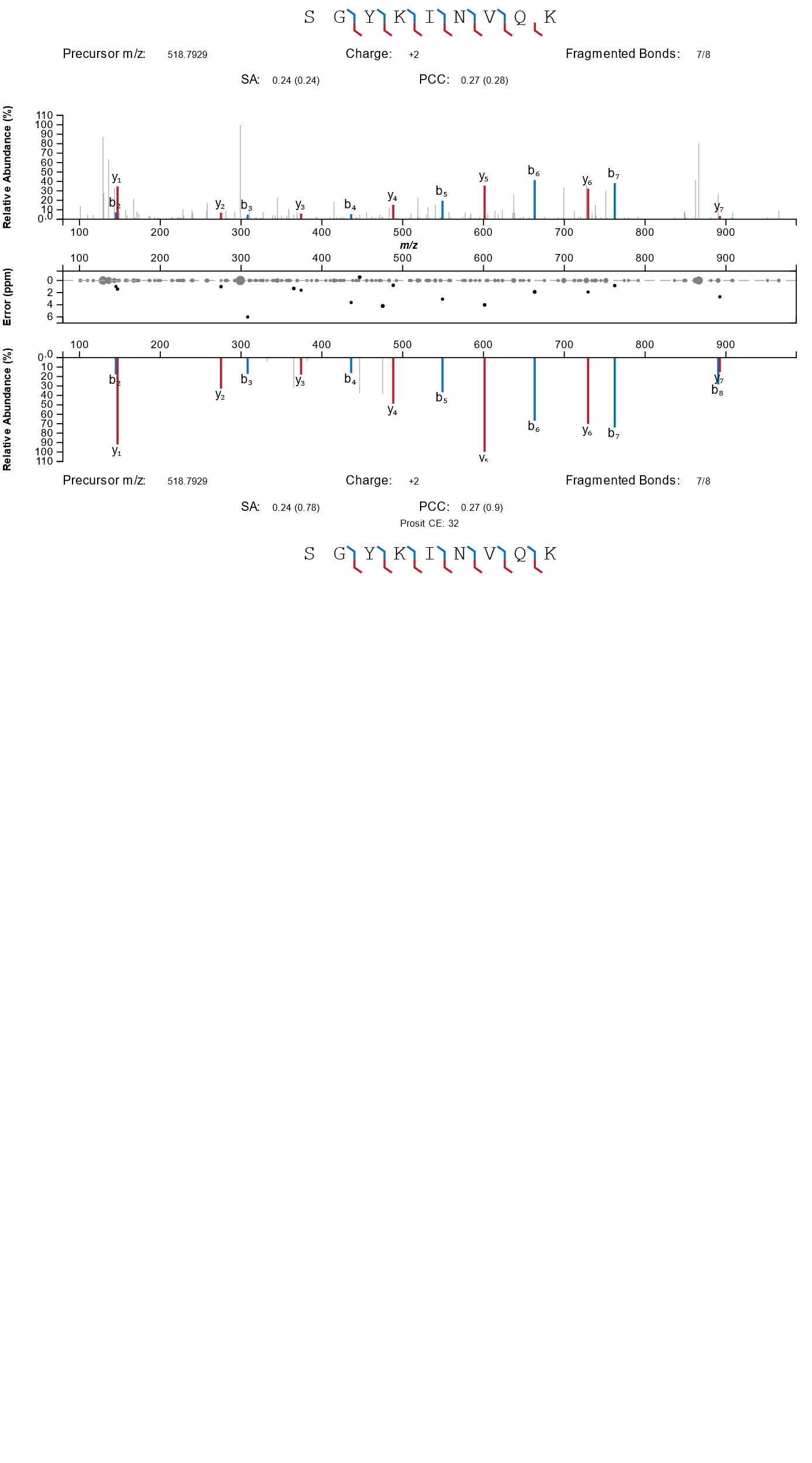

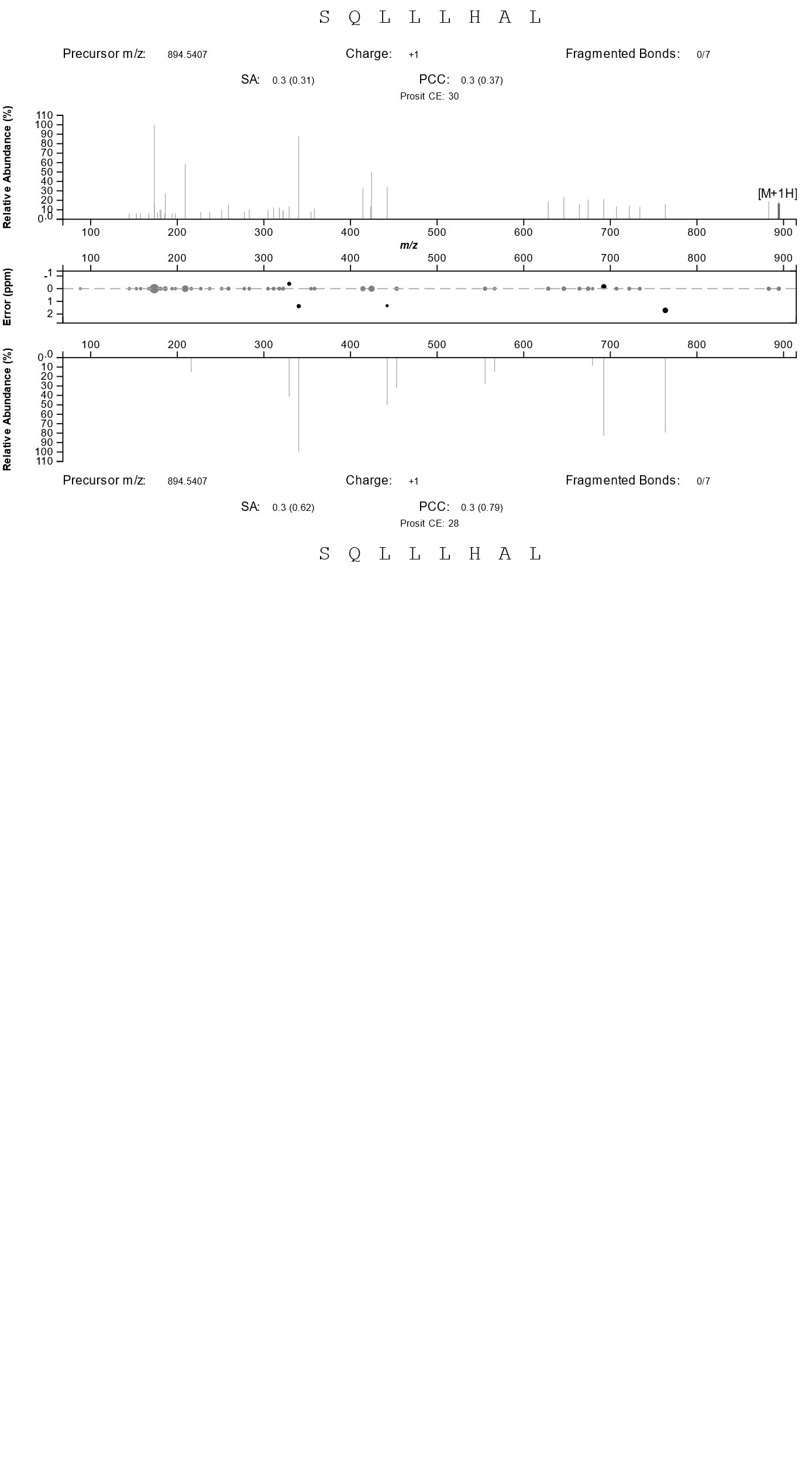

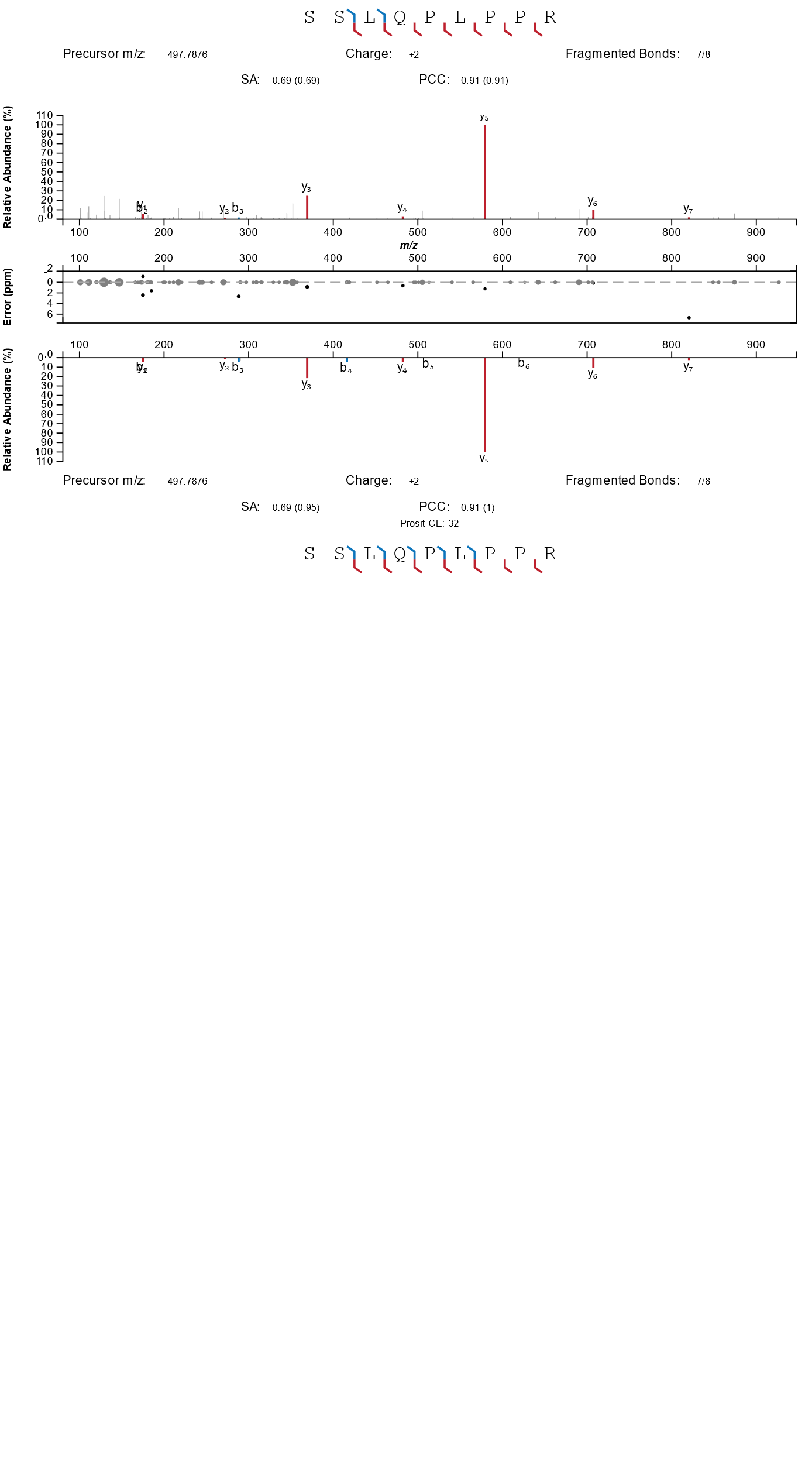

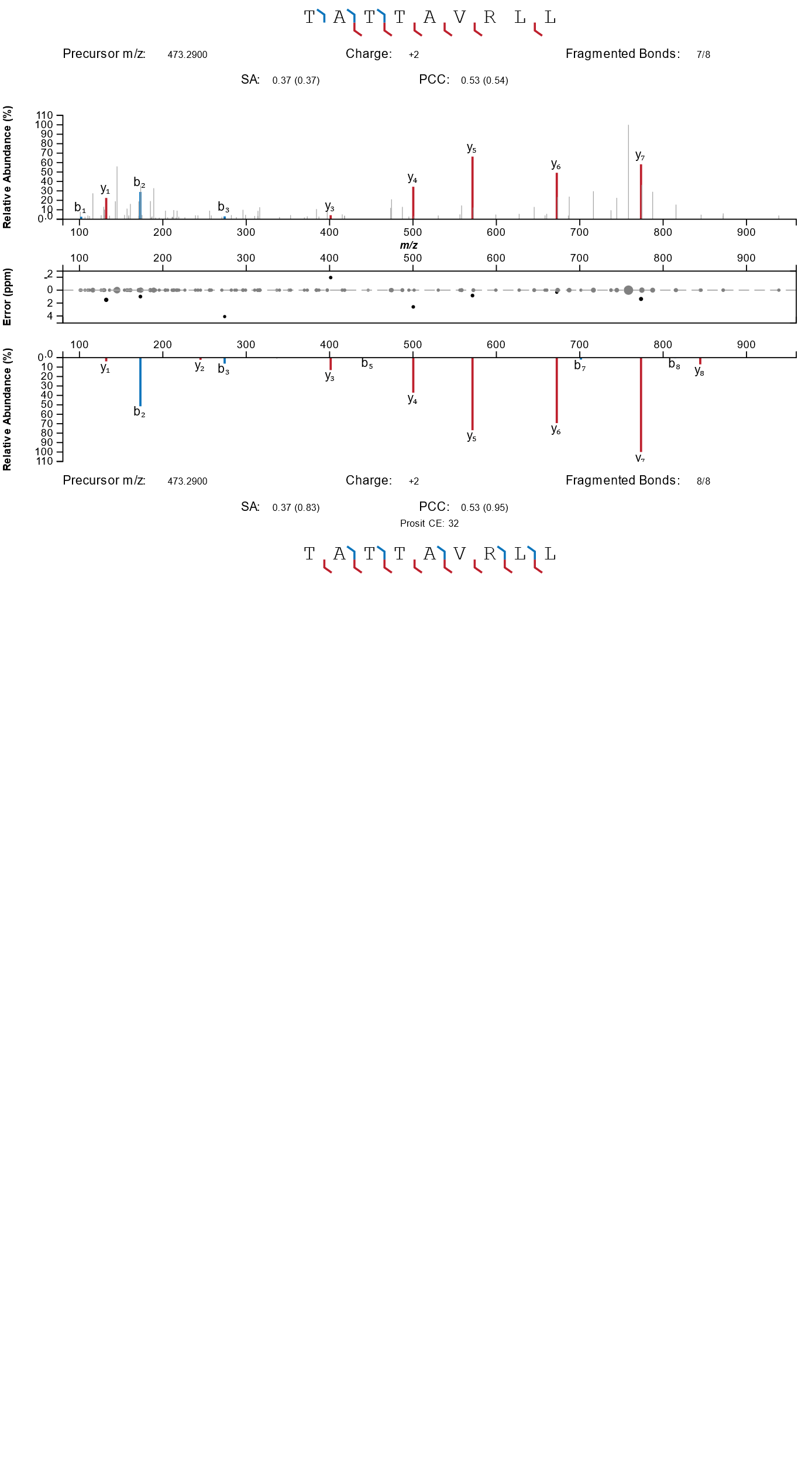

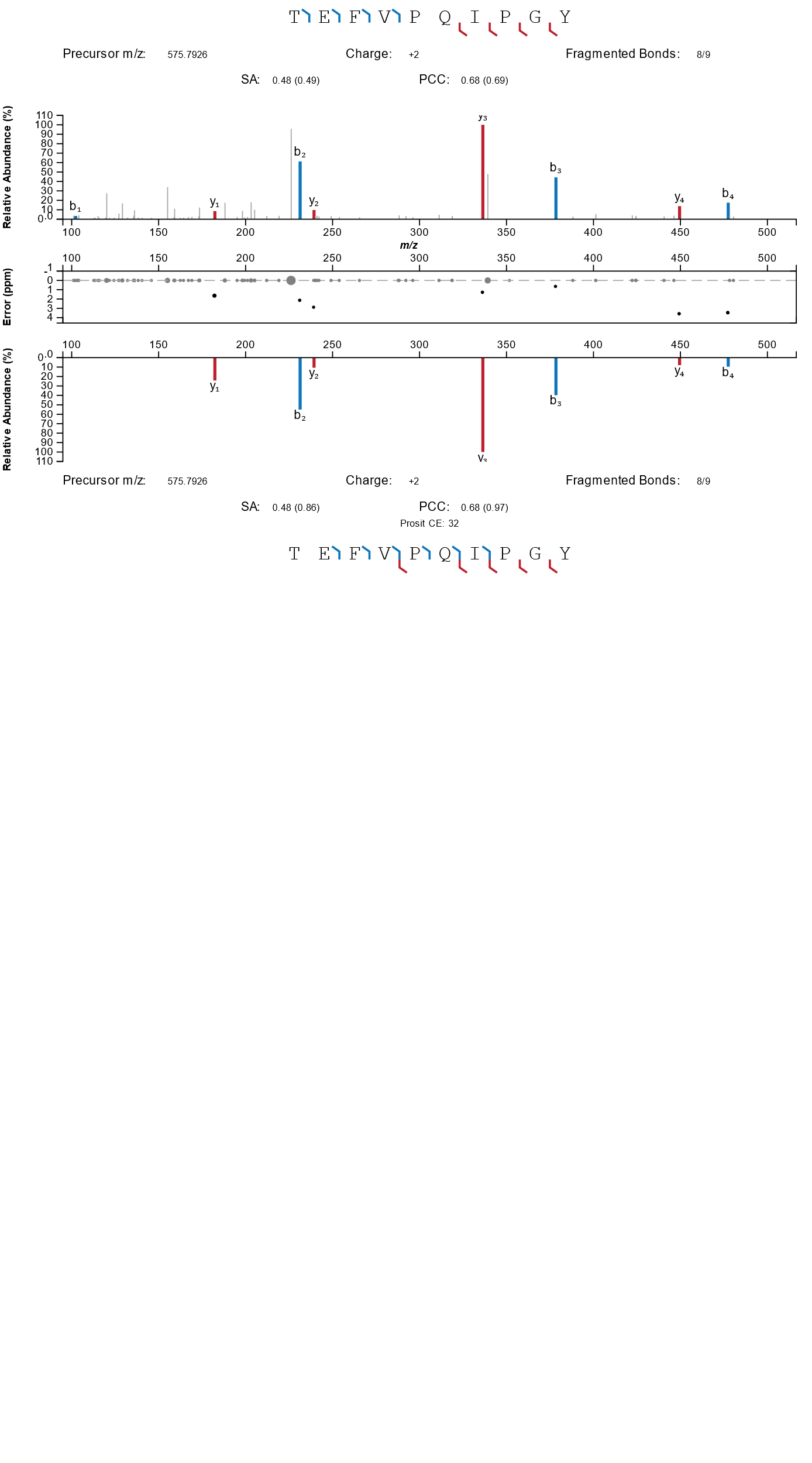

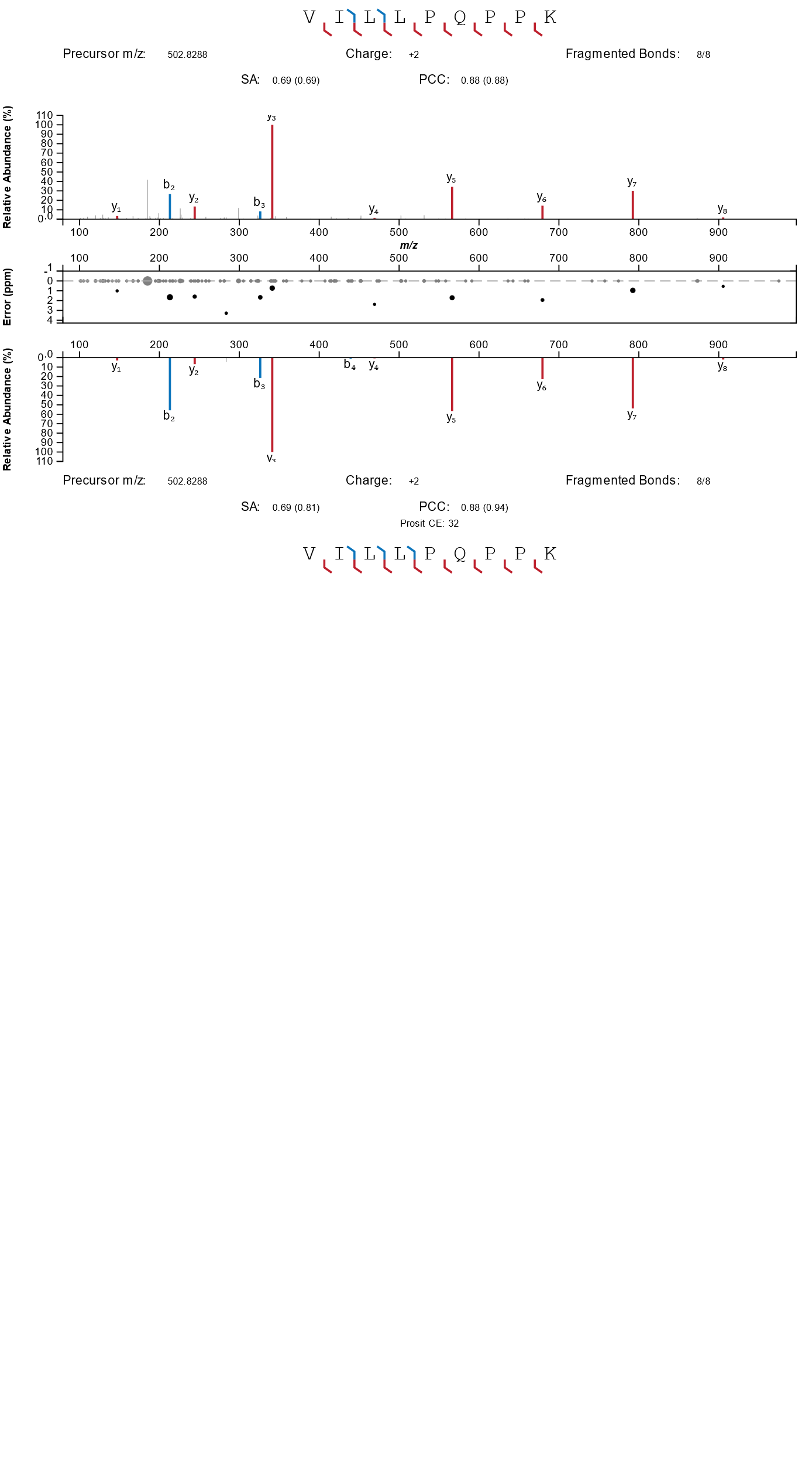

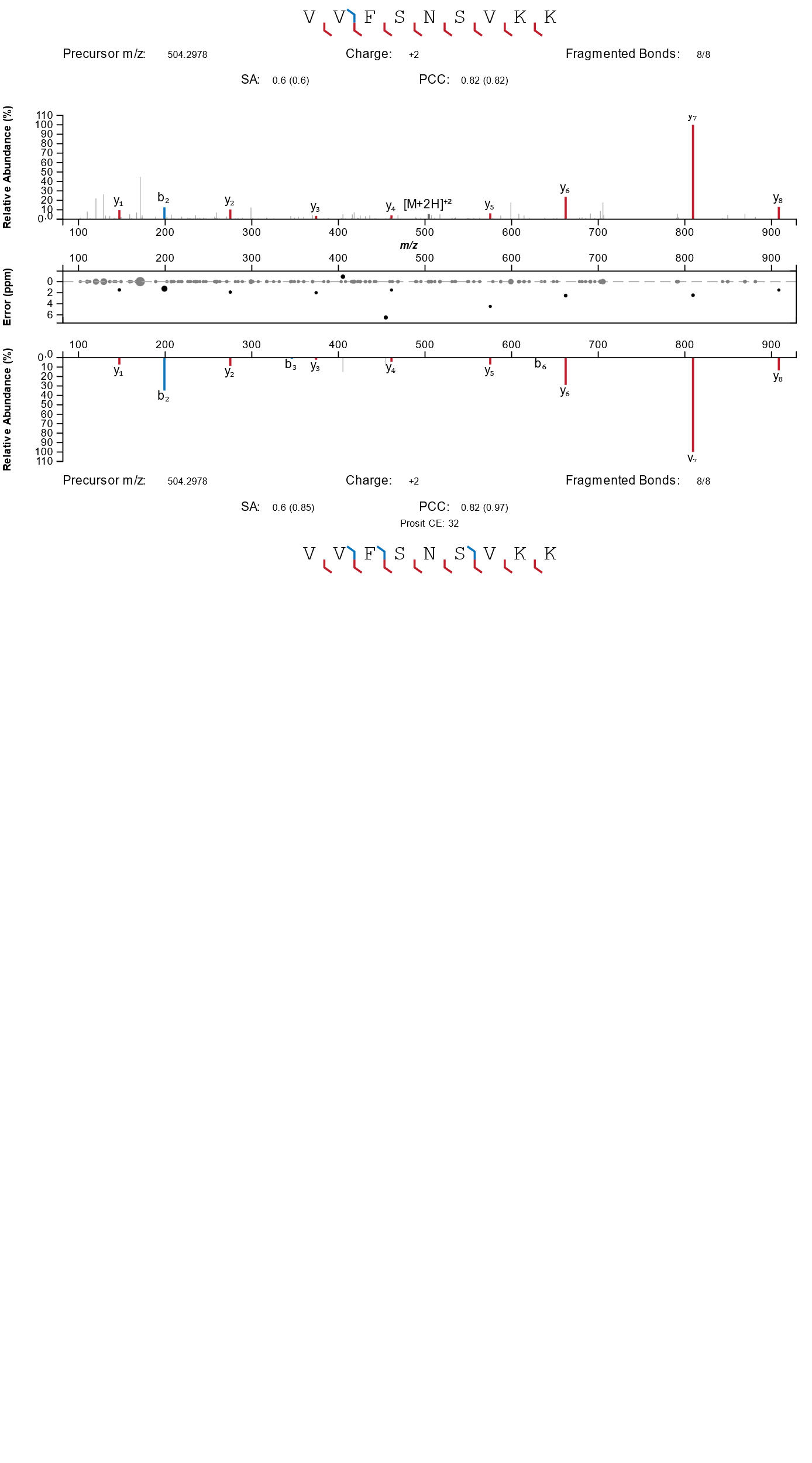

Supplement: Supplementary Figure [file mmc6.docx]
